# Design of Closed-Loop Control Schemes Based on the GA-PID and GA-RBF-PID Algorithms for Brain Dynamic Modulation

**DOI:** 10.3390/e25111544

**Published:** 2023-11-15

**Authors:** Chengxia Sun, Lijun Geng, Xian Liu, Qing Gao

**Affiliations:** 1 Mechanical and Electrical Engineering College, Hebei Normal University of Science and Technology, Qinhuangdao 066004, China; sunchengxia2021@126.com (C.S.); li_jungeng@163.com (L.G.); 2 State Key Laboratory of Intelligent Rehabilitation and Neuromodulation of Hebei Province, Institute of Electrical Engineering, Yanshan University, Qinhuangdao 066004, China; ysu_gq@ysu.edu.cn

**Keywords:** GA-PID algorithm, GA-RBF-PID algorithm, modulation of brain dynamics, Wendling-type coupled neural mass model, control accuracy, dynamic performance and stability

## Abstract

Neurostimulation can be used to modulate brain dynamics of patients with neuropsychiatric disorders to make abnormal neural oscillations restore to normal. The control schemes proposed on the bases of neural computational models can predict the mechanism of neural oscillations induced by neurostimulation, and then make clinical decisions that are suitable for the patient’s condition to ensure better treatment outcomes. The present work proposes two closed-loop control schemes based on the improved incremental proportional integral derivative (PID) algorithms to modulate brain dynamics simulated by Wendling-type coupled neural mass models. The introduction of the genetic algorithm (GA) in traditional incremental PID algorithm aims to overcome the disadvantage that the selection of control parameters depends on the designer’s experience, so as to ensure control accuracy. The introduction of the radial basis function (RBF) neural network aims to improve the dynamic performance and stability of the control scheme by adaptively adjusting control parameters. The simulation results show the high accuracy of the closed-loop control schemes based on GA-PID and GA-RBF-PID algorithms for modulation of brain dynamics, and also confirm the superiority of the scheme based on the GA-RBF-PID algorithm in terms of the dynamic performance and stability. This research of making hypotheses and predictions according to model data is expected to improve and perfect the equipment of early intervention and rehabilitation treatment for neuropsychiatric disorders in the biomedical engineering field.

## 1. Introduction

As early as the 1970s, Wiener and Schadé emphasized the importance of establishing mathematical models that are isomorphic to biological systems from the perspective of information processing for the development of biomedical engineering [1]. The well-established mathematical models for simulating different neural oscillations recorded by electroencephalogram (EEG) help to better explain the brain functional states and the evolutionary mechanisms of neuropsychiatric disorders [2,3], and then provide theoretical bases for seeking modulation methods of abnormal neural oscillations [4,5]. Non-linear neural mass models are commonly used models for simulating neural oscillations [6], in which the changes of the excitatory gains have a significant influence on the dynamical characteristics of the models, and these changes are usually used to simulate the generation of abnormal neural oscillations. Research on the modulation of brain dynamics based on neural mass models plays an important role in the field of biomedical engineering, which can provide ideas and scientific bases for the optimization and innovation of early intervention and rehabilitation treatment equipment for neuropsychiatric patients with abnormal neural oscillations [7]. In addition to mathematical models, control algorithms also directly affect the performance of control schemes.

To ensure the performance of the brain dynamics modulation scheme, it is necessary to comprehensively consider factors such as modulation target, energy consumption and external interference to introduce different control algorithms. The algorithms commonly used in the modern engineering control theory, such as PID [8], unscented Kalman filter (UKF) [9], particle swarm optimization (PSO) [10], etc., have been extended to the field of computational neuroscience. Su et al. proposed an adaptive feedback linearization algorithm that enhanced the robustness to uncertain factors of system and outside world for the highly non-linear computational model of Parkinson’s disease with unknown parameters [11]. The generalized predictive closed-loop control strategy put forward by Liu et al. could generate adaptive stimulation waveforms with low energy expenditure without requiring any particular knowledge of model’s physiological properties, which had potential to improve the therapeutic effect for Parkinson’s disease [12]. From the point of filtering, a detection and closed-loop control strategy based on the UKF algorithm was proposed for the neural mass model used to simulate epileptic rhythms [13]. On this foundation, a fuzzy adaptive closed-loop control method based on the UKF algorithm was put forward, which was used in the case of noise uncertainty [14]. Further, the UKF-based closed-loop iterative learning control accelerated the convergence rate of control errors [15]. Shan et al. developed a closed-loop control strategy based on the PSO algorithm for the neural mass model [16], which significantly improved the estimation effect of unmeasurable parameters and the efficiency of control compared with the UKF. For systems that do not consider external interference factors, if there is a high demand for control accuracy, the PID-related algorithms with the longest history, the widest application, and the strongest adaptability are the optimal algorithms for designing brain dynamics modulation schemes. Gorzelic et al. have introduced the PID algorithm into the closed-loop control of the computational model of Parkinson’s disease dynamics because the algorithm has a simple control structure and remarkable tracking performance for repetitive processes in nature [17]. Similarly, proportional-integral and proportional-derivative algorithms are also commonly used for the closed-loop control of computational models for neuropsychiatric disorders [18,19,20]. The traditional PID algorithm has two main drawbacks. First, the quality of PID parameters directly determines the performance of the algorithm, however, the choice of PID parameters largely depends on the designers’ experience. Second, the adaptability of the algorithm is an important indicator to measure its performance, but online adaptive adjustment of PID parameters is a difficult problem for the conventional PID control. For the PID control in neuroscience, these problems also exist.

To overcome the above shortcomings, we introduce the RBF neural network [21] and GA [22] to the incremental PID closed-loop feedback control scheme to modulate the dynamics of a class of neural mass models. The purpose of introducing GA is to determine (initial) values of PID parameters to ensure the control accuracy. For the sake of reducing dependence of the incremental PID control on controlled model and improving the dynamic performance and stability of the control method, a GA-RBF-PID algorithm based control scheme is further proposed. This method reconstructs outputs of the controlled Wendling-type coupled neural mass model by applying RBF neural network’s ability of approximating arbitrary non-linear functions while automatically tuning and modifying PID parameters online by applying RBF neural network’s self-learning ability. The present work is expected to establish a bridge between clinical and fundamental theoretical research on neuropsychiatric disorders.

## 2. Materials and Methods

### 2.1. Wendling-Type Coupled Neural Mass Model

Various mathematical models have been developed to simulate neural oscillation activities to meet the theoretical research needs of the biomedical field. The increasingly well-established neural mass models not only provide valuable means to explore mechanisms of rhythms underlying certain brain activities or phenomena, so as to describe generation processes of neuropsychiatric disorders (such as epilepsy), but also use to verify the effectiveness of various EEG signal processing methods that are conducive to the analysis and understanding of real EEG data. In short, the neural mass model seems to be a good compromise between the tractability and realizability of the modulation research of brain dynamics [23].

**Figure 1:**
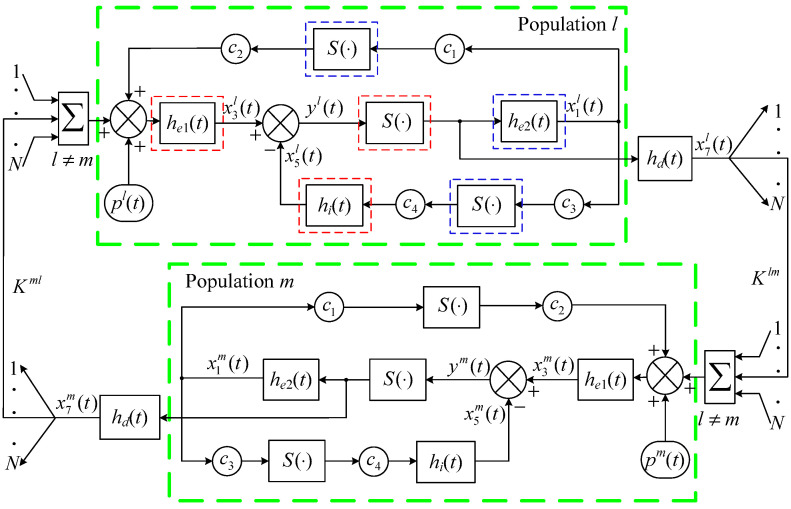
Structure block diagram of the Wendling-type coupled neural mass model. (The part within the red dashed box represents the subset of main cells. The part within the blue dashed box represents the subset of interneurons).

Figure 1 shows the structure diagram of the Wendling-type coupled neural mass model, where the integer *N* (*N* > 2) indicates the quantity of neural populations under consideration, and superscripts *l* and *m* indicate the *l*-th and *m*-th populations, respectively. Since the populations *l* and *m* are similar, only the population *l* is described in detail here. The block diagram in Figure 1 describes the information flow among two subsets of population *l*, and among coupled populations with population *l*. The subset of main cells (the part within the red dashed box) consists of pyramidal cells, which receives both excitatory and inhibitory feedback from the subset of interneurons (the part within the blue dashed box) and a noise input $p^{l}$(*t*). Here $p^{l}$(*t*) globally denotes the average density of afferent action potentials from neighboring or distant populations. It can be arbitrary function including white noise. The subset of interneurons consists of a few pyramidal cells and stellate or basket cells, which receives excitatory input only from the subset of main cells. There are three main variables in population *l*, denoted by $x_{1}^{l}$(*t*), $x_{3}^{l}$(*t*) and $x_{5}^{l}$(*t*), where $x_{1}^{l}$(*t*) is the output of the excitatory postsynaptic potential transfer function $h_{e2}$(*t*); $x_{3}^{l}$(*t*) and $x_{5}^{l}$(*t*) are the outputs of the excitatory postsynaptic potential and inhibitory postsynaptic potential transfer functions $h_{e1}$(*t*) and $h_{i}$(*t*), respectively. In addition, a variable is closely related to the population *l*, that is, the output of the excitatory postsynaptic potential transfer function $h_{d}$(*t*) denoted by $x_{7}^{l}$(*t*), where $h_{d}$(*t*) is used to model delays related to connections from population *l*. The output $y^{l}\left(t\right)$ is used to simulate neural oscillations recorded by EEG. The static non-linear function denoted by the symbol *S*(·) associates the membrane potential level of neurons with the average pulse density of the potential fired by these neurons. *S*(·) has a sigmoid form

(1) $$\begin{array}{c} S\left(v\right)=\frac{2e_{0}}{1+e^{r(v_{0}-v)}}, \end{array}$$

where *v*, $e_{0}$, $v_{0}$, and *r* are parameters that determine its voltage sensitivity. The average numbers of synaptic connections between main cells and interneurons are represented by the connectivity constants $c_{1}$–$c_{4}$. The coupling strength $K^{ml}$ characterizes the degree of dependence of population *l* on population *m*, and $K^{ml}>0$. The mathematical expression of the *l*-th population in the Wendling-type coupled neural mass model has the form

(2) x˙l(t)=Axl(t)+BS(Hxl(t))+B1pl(t)+D∑m=1,m≠lNKmlxm(t),yl(t)=Cxl(t),

where $x^{l}\left(t\right)={\left[x_{1}^{l}\left(t\right)\;x_{2}^{l}\left(t\right)\;\cdots \;x_{8}^{l}\left(t\right)\right]}^{T}$ is the state vector of the population under consideration and $x_{2}^{l}$(*t*), $x_{4}^{l}$(*t*), $x_{6}^{l}$(*t*), $x_{8}^{l}$(*t*) are time derivatives of $x_{1}^{l}$(*t*), $x_{3}^{l}$(*t*), $x_{5}^{l}$(*t*), $x_{7}^{l}$(*t*), respectively; $x^{m}\left(t\right)$ represents the state vector of the *m*-th population, and since the form of $x^{m}\left(t\right)$ is similar to $x^{l}\left(t\right)$, it will not be repeated; constant matrices *A*, *B*, $B_{1}$, *H*, *D*, and *C* with the form of

$$\begin{array}{cc} \; & A=\mathrm{diag}\left(A_{1},\ldots ,A_{4}\right),A_{j}=\left[\begin{array}{cc} 0 & 1\\ -\mu_{j}^{2} & -2\mu_{j} \end{array}\right],j=1,2,3,4,\\ \; & \mu_{1}=\mu_{2}=a,\mu_{3}=b,\mu_{4}=a_{d},\\ \; & B={\left[\begin{array}{cccccccc} 0 & \theta_{A}a & 0 & 0 & 0 & 0 & 0 & \theta_{A}a_{d}\\ 0 & 0 & 0 & \theta_{A}ac_{2} & 0 & 0 & 0 & 0\\ 0 & 0 & 0 & 0 & 0 & \theta_{B}bc_{4} & 0 & 0 \end{array}\right]}^{T},\\ \; & B_{1}={\left[\begin{array}{cccccccc} 0 & 0 & 0 & \theta_{A}a & 0 & 0 & 0 & 0 \end{array}\right]}^{T},\\ \; & H=\left[\begin{array}{cccccccc} 0 & 0 & 1 & 0 & -1 & 0 & 0 & 0\\ c_{1} & 0 & 0 & 0 & 0 & 0 & 0 & 0\\ c_{3} & 0 & 0 & 0 & 0 & 0 & 0 & 0 \end{array}\right],\\ \; & D=\left[\begin{array}{cccccccc} 0 & 0 & 0 & 1 & 0 & 0 & 0 & 0 \end{array}\right],\\ \; & C=\left[\begin{array}{cccccccc} 0 & 0 & 1 & 0 & -1 & 0 & 0 & 0 \end{array}\right]. \end{array}$$

For simplicity of writing, the superscript *l* is not add to parameters $\theta_{A}$, $\theta_{B}$, *a*, *b*, $a_{d}$, $c_{1}$–$c_{4}$, but these parameters may vary from one population to another. It has been confirmed that certain kinds of neural mass models can generate neural oscillations reflecting normal brain activities and neuropsychiatric disorders (such as temporal lobe seizure) [17,19]. Table 1 presents physiological meanings and standard values of all internal parameters in the model [4]. The model can produce well-defined α-like oscillation with these standard values. The α oscillation is considered as the EEG signal in resting state. Previous studies have shown that increasing the excitatory gains of certain neural populations in the model to deviate them from the standard value is a commonly used method for the neural mass model to simulate abnormal neural oscillations under pathological conditions, and then spikes may propagate from these populations to others under the effect of coupling, which will make the situation worse. We introduce the Wendling-type coupled neural mass model consisting of *N* identical neural populations on the Formula (2) base, which mathematical expression is as follows

(3) $$\begin{array}{c} \dot{x}\left(t\right)=f\left(x(t),p(t)\right),\\ y\left(t\right)=g\left(x(t)\right), \end{array}$$

where $x\left(t\right)={\left[x^{1}\left(t\right)\;x^{2}\left(t\right)\;\cdots \;x^{N}\left(t\right)\right]}^{T}$, $y\left(t\right)=[y^{1}\left(t\right)$$y^{2}\left(t\right)\;\cdots \;y^{N}{\left(t\right)]}^{T}$, $p\left(t\right)=[p^{1}\left(t\right)\;p^{2}\left(t\right)$$\cdots \;p^{N}{\left(t\right)]}^{T}$, f(·) and g(·) are the input and output functions respectively.

### 2.2. Design of Closed-Loop Control Schemes for Brain Dynamic Modulation

#### 2.2.1. Preliminary Knowledge

In the research of modern control system theory, in order to facilitate computer implementation, it is usually necessary to discretize continuous systems and algorithms. A typical class of continuous controlled non-linear systems, including the Wendling-type coupled neural mass model (3) aforementioned, can be discretized into

(4) $$\begin{array}{c} x\left(k\right)=f_{d}\left(x(k-1),p(k-1)\right)+Fu\left(k-1\right),\\ y_{c}\left(k\right)=g_{d}\left(x(k)\right), \end{array}$$

where *k* indicates the sampling time point; $x\left(k\right)\in R^{N_{x}}$ is the state vector of the controlled system, $R^{N_{x}}$ indicates $N_{x}$—dimensional real column vector; $y_{c}\left(k\right)\in R^{N_{y}}$ is the controlled output vector; $p\left(k\right)\in R^{N_{p}}$ is the known internal input of the model; $u\left(k\right)\in R^{N_{u}}$ represents the external control input; the control input matrix $F\in R^{N_{x}\times N_{u}}$, in which $R^{N_{x}\times N_{u}}$ represents $N_{x}\times N_{u}$—dimensional real matrix; $f_{d}\left(\cdot \right):R^{N_{x}\times N_{p}}\to R^{N_{x}}$ and $g_{d}\left(\cdot \right):R^{N_{x}}\to R^{N_{y}}$ can be derived from the known continuous functions.

The control method based on the incremental PID algorithm commonly used for regulating the dynamics of non-linear system has been widely used in the field of neuroengineering for modulating brain dynamics. The traditional incremental PID algorithm performs proportional, integral, and differential operations on control errors, and linearly combines them into changes in external control inputs. The control law is as follows:

(5) $$\begin{array}{c} u(k)=u(k-1)+\Delta u(k),\\ \Delta u\left(k\right)=K_{p}e_{2}\left(k\right)+K_{i}e_{1}\left(k\right)+K_{d}e_{3}\left(k\right), \end{array}$$

where Δu(k) represents the variation of the external control input u(k); $e_{1}\left(k\right)=e\left(k\right)$, $e_{2}\left(k\right)=e\left(k\right)-e\left(k-1\right)$, $e_{3}\left(k\right)=e\left(k\right)-2e\left(k-1\right)+e\left(k-2\right)$, in which e(k) indicates the control error; $K_{p}$, $K_{i}$, $K_{d}$ are the PID parameters to be set, $K_{p}$ indicates the proportional coefficient, $K_{i}$ indicates the integral coefficient, and $K_{d}$ is the differential coefficient. This kind of PID control has two obvious shortcomings: the selection of PID parameters largely depends on the designers’ experience, and the adaptive ability is poor. Therefore, this section proposes two closed-loop control schemes based on improved incremental PID algorithms, namely the GA-PID algorithm and the GA-RBF-PID algorithm, to modulate the brain dynamics simulated by the Wendling-type coupled neural mass model.

#### 2.2.2. GA-PID Algorithm Based Closed-Loop Control Scheme

**Figure 2:**
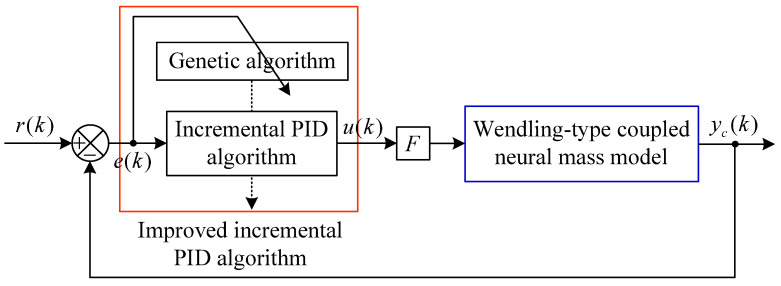
Structure diagram of the brain dynamic modulation system based on the GA-PID algorithm.

The GA-PID algorithm mainly utilizes the GA to determine control parameters of the incremental PID algorithm in the brain dynamic modulation system, thereby ensuring control accuracy. The basic structural framework of the whole control scheme is shown in Figure 2, where r(k) indicates the expected output vector of the controlled Wendling-type coupled neural mass model; $y_{c}\left(k\right)$ indicates the controlled output vector; the control error vector $e\left(k\right)=r\left(k\right)-y_{c}\left(k\right)$; u(k) is the external control input; *F* is the control input matrix composed only of 0 and 1, and its form determines the controlled neural populations.

The basic steps of GA to solve the control parameters of the incremental PID algorithm are as follows:

(1) Determine approximate ranges of the control parameters $K_{p}$, $K_{i}$ and $K_{d}$ respectively, and then fix the coding length *L* of the individual chromosome to prepare for binary coding;

(2) Randomly generate binary coding chromosomes of $Q_{m}\times 3$ individuals, forming the initial community P(1), where $Q_{m}$ denotes the population number, and each population contains three individuals, namely $K_{p}$, $K_{i}$ and $K_{d}$;

(3) The chromosome of each individual in the population is decoded into the corresponding PID parameter. Here is an example to illustrate the decoding process of the PID parameter: if the proportional coefficient decoded from the *m*-th $\left(m=1,2,\cdots ,Q_{m}\right)$ population is represented by $K_{p}^{m}$, and its value varies within the preset range $\left[K_{p\min},K_{p\max}\right]$. Assuming that the chromosome of this individual is coded as a binary string $\beta_{L}\beta_{L-1}\beta_{L-2}\cdots$$\beta_{2}\beta_{1}$, then

(6) $$\begin{array}{c} K_{p}^{m}=K_{p\min}+\frac{\sum_{j=1}^{L}2^{j-1}\beta_{j}}{2^{L}-1}\left(K_{p\max}-K_{p\min}\right); \end{array}$$

(4) Compute the objective function

(7) $$\begin{array}{c} J_{1}\left(i\right)=\sum_{k=1}^{n}\left|e(k)\right|, \end{array}$$

where *k* is the sampling time point, *n* is the number of sampling times, $i=1,2,\cdots ,Q_{i}$ and $Q_{i}$ represents the genetic algebra, and $\left|\cdot \right|$ is the absolute value symbol;

(5) The population P(i) is genetically manipulated using selection, crossover, and mutation operators to generate the next generation population P(i+1);

(6) Repeat steps (3) to (5) until the objective function converges or the GA parameters reach the preset indexes. Select $K_{p}$, $K_{i}$ and $K_{d}$ from the *n* generation results that can achieve or approach the minimum control error as the parameters of the incremental PID algorithm.

#### 2.2.3. GA-RBF-PID Algorithm Based Closed-Loop Control Scheme

**Figure 3:**
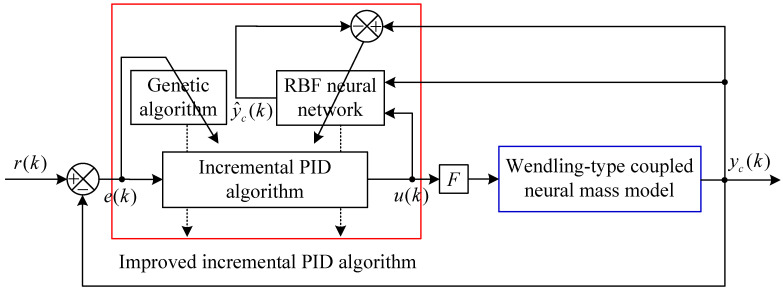
Structure diagram of the brain dynamic modulation system based on the GA-RBF-PID algorithm.

The GA-RBF-PID algorithm mainly utilizes the GA to determine the initial values of PID parameters in the brain dynamic modulation framework offline, thereby ensuring the control accuracy, and utilizes the RBF neural network to adaptively adjust and modify these three control parameters online, thereby improving the dynamic performance and stability of the control system. The basic structural framework of the whole control scheme is shown in Figure 3, where ${\hat{y}}_{c}\left(k\right)$ indicates the estimated controlled output vector obtained by the RBF neural network.

**Figure 4:**
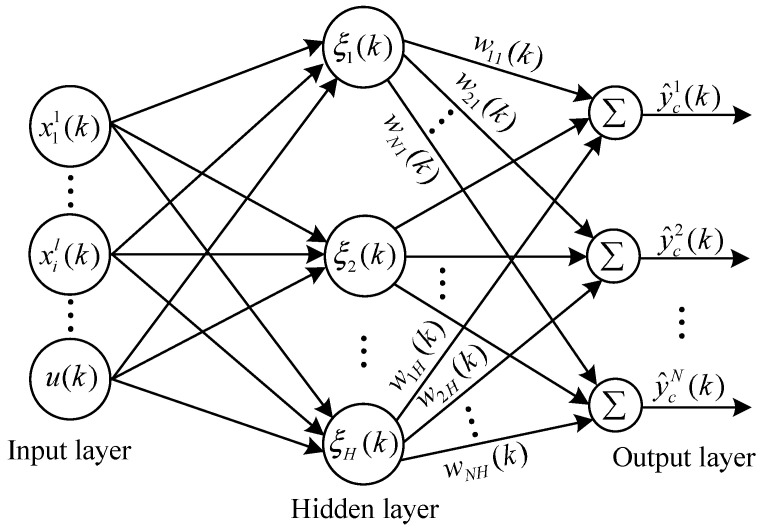
Structure diagram of the RBF neural network.

RBF-PID is a new control algorithm that integrates the control law of the incremental PID algorithm into the RBF neural network, that is, the control law at the sampling time point *k* shown in Equation (5) is integrated into the *k*-th group of sample data of the RBF neural network. The specific approach is to use the external control input u(k) as one of the input sample data in the *k*-th group of sample data of the RBF neural network, and then use the Jacobian information provided by the RBF estimator to adjust $K_{p}$, $K_{i}$ and $K_{d}$ in Equation (5) online, thereby achieving self-tuning of PID parameters. Figure 4 shows the basic structural diagram of the RBF neural network in Figure 3. It can be seen that the RBF neural network is a three-layer forward network with a single hidden layer, where each node of the input layer corresponds to an element of the input vector $x_{r}\left(k\right)={\left[x_{1}^{1}\left(k\right)\;x_{2}^{1}\left(k\right)\cdots \;x_{8}^{1}\left(k\right)\cdots \;x_{i}^{l}\left(k\right)\cdots \;u\left(k\right)\right]}^{T}$, $x_{i}^{l}\left(k\right)$ is the i(i=1,2,⋯,8)-th state variable of the l(l=1,2,⋯,N)-th population in the Wendling-type coupled neural mass model, *N* is the total number of neural populations, u(k) is the external control input, then the number of input nodes is M=8N+1. For the purpose of easy representation, let $x_{r}\left(k\right)={\left[x_{1}\left(k\right)\;x_{2}\left(k\right)\cdots \;x_{m}\left(k\right)\cdots \;x_{M}\left(k\right)\right]}^{T}$, in which m=1,2,⋯,M. Each hidden node of the hidden layer corresponds to an element of the radial vector $\xi \left(k\right)={\left[\xi_{1}\left(k\right)\;\xi_{2}\left(k\right)\;\cdots \;\xi_{H}\left(k\right)\right]}^{T}$, where subscript *H* is the number of hidden nodes, and the Gaussian kernel radial basis activation function [24] corresponding to the *j*-th hidden node can be expressed as

(8) $$\begin{array}{c} \xi_{j}\left(k\right)=\exp\left[-\frac{{∥x\left(k\right)-\varepsilon_{j}\left(k\right)∥}^{2}}{2{\left(\theta_{j}\left(k\right)\right)}^{2}}\right],j=1,2,\cdots ,H, \end{array}$$

where $\varepsilon_{j}={\left[\varepsilon_{j1}\;\varepsilon_{j2}\;\cdots \;\varepsilon_{jM}\right]}^{T}$ and $\theta_{j}$ represent the center parameter vector and width parameter used to determine the radial action range of the activation function; $∥\cdot ∥$ indicates the Euclidean norm on the input space; the weight vector $W\left(k\right)=[w_{1}\left(k\right)\;w_{2}\left(k\right)\;\cdots \;w_{H}\left(k\right)]$ indicates the weight matrix from the hidden layer to the output layer, and $w_{j}\left(k\right)={\left[w_{1j}\left(k\right)\;w_{2j}\left(k\right)\;\cdots \;w_{Nj}\left(k\right)\right]}^{T}$. The RBF neural network is used as an estimator to approximate the controlled output of the Wendling-type coupled neural mass model obtaining the estimated controlled output vector ${\hat{y}}_{c}\left(k\right)=W\left(k\right)\xi \left(k\right)={\left[{\hat{y}}_{c}^{1}\left(k\right)\;{\hat{y}}_{c}^{2}\left(k\right)\;\cdots \;{\hat{y}}_{c}^{N}\left(k\right)\right]}^{T}$, and ${\hat{y}}_{c}^{1}\left(k\right)$ corresponding to the *l*-th output node of the output layer is

(9) $${\hat{y}}_{c}^{l}\left(k\right)=\sum_{j=1}^{H}w_{lj}\left(k\right)\xi_{j}\left(k\right).$$

Make the performance index of the RBF neural network a quadratic function

(10) $$\begin{array}{c} J_{2}\left(k\right)=\frac{1}{2}{\left(y_{c}\left(k\right)-{\hat{y}}_{c}\left(k\right)\right)}^{2}. \end{array}$$

In order to reduce estimation errors, the gradient descent method is used to correct the weight coefficient $w_{lj}$, center parameter $\varepsilon_{jm}$, and width parameter $\theta_{j}$ of the RBF neural network. The specific correction formula is as follows

(11) $$\begin{array}{c} w_{lj}\left(k\right)=w_{lj}\left(k-1\right)+\Delta w_{lj}\left(k\right)+\alpha \left(w_{lj}\left(k-1\right)-w_{lj}\left(k-2\right)\right),\\ \Delta w_{lj}\left(k\right)=-\eta \frac{\partial J_{2}\left(k\right)}{\partial w_{lj}\left(k\right)}=-\eta \frac{\partial J_{2}\left(k\right)}{\partial {\hat{y}}_{c}^{l}\left(k\right)}\frac{{\hat{y}}_{c}^{l}\left(k\right)}{\partial w_{lj}\left(k\right)}=\eta \left(y_{c}^{l}\left(k\right)-{\hat{y}}_{c}^{l}\left(k\right)\right)\xi_{j}\left(k\right),\\ \theta_{j}\left(k\right)=\theta_{j}\left(k-1\right)+\Delta \theta_{j}\left(k\right)+\alpha \left(\theta_{j}\left(k-1\right)-\theta_{j}\left(k-2\right)\right),\\ \Delta \theta_{j}\left(k\right)=-\eta \frac{\partial J_{2}\left(k\right)}{\partial \theta_{j}\left(k\right)}=-\eta \frac{\partial J_{2}\left(k\right)}{\partial {\hat{y}}_{c}^{T}\left(k\right)}\frac{{\hat{y}}_{c}\left(k\right)}{\partial \theta_{j}\left(k\right)}=\eta {\left(y_{c}\left(k\right)-{\hat{y}}_{c}\left(k\right)\right)}^{T}w_{j}\left(k\right)\xi_{j}\left(k\right)\frac{{∥x\left(k\right)-\varepsilon_{j}\left(k\right)∥}^{2}}{{\left(\theta_{j}\left(k\right)\right)}^{3}},\\ \varepsilon_{jm}\left(k\right)=\varepsilon_{jm}\left(k-1\right)+\Delta \varepsilon_{jm}\left(k\right)+\alpha \left(\varepsilon_{jm}\left(k-1\right)-\varepsilon_{jm}\left(k-2\right)\right),\\ \Delta \varepsilon_{jm}\left(k\right)=-\eta \frac{\partial J_{2}\left(k\right)}{\partial \varepsilon_{jm}\left(k\right)}=-\eta \frac{\partial J_{2}\left(k\right)}{\partial {\hat{y}}_{c}^{T}\left(k\right)}\frac{{\hat{y}}_{c}\left(k\right)}{\partial \varepsilon_{jm}\left(k\right)}=\eta {\left(y_{c}\left(k\right)-{\hat{y}}_{c}\left(k\right)\right)}^{T}w_{j}\left(k\right)\xi_{j}\left(k\right)\frac{x_{m}\left(k\right)-\varepsilon_{jm}\left(k\right)}{{\left(\theta_{j}\left(k\right)\right)}^{2}}, \end{array}$$

where η represents the learning rate; α∈[0,1) represents the inertia coefficient that is conducive to accelerating the learning process.

The gradient descent method can also be used to correct the control parameters $K_{p}$, $K_{i}$ and $K_{d}$ of the incremental PID algorithm

(12) $$\begin{array}{c} K_{p}\left(k\right)=K_{p}\left(k-1\right)+\Delta K_{p}\left(k\right),\Delta K_{p}\left(k\right)=-\eta_{p}\frac{\partial J_{2}\left(k\right)}{\partial K_{p}\left(k\right)},\\ K_{i}\left(k\right)=K_{i}\left(k-1\right)+\Delta K_{i}\left(k\right),\Delta K_{i}\left(k\right)=-\eta_{i}\frac{\partial J_{2}\left(k\right)}{\partial K_{i}\left(k\right)},\\ K_{d}\left(k\right)=K_{d}\left(k-1\right)+\Delta K_{d}\left(k\right),\Delta K_{d}\left(k\right)=-\eta_{d}\frac{\partial J_{2}\left(k\right)}{\partial K_{d}\left(k\right)}, \end{array}$$

where $\eta_{p}$, $\eta_{i}$, and $\eta_{d}$ are all the learning rates; $K_{p}\left(k\right)$, $K_{i}\left(k\right)$ and $K_{d}\left(k\right)$ represent PID parameters at the sampling time point *k*. Since $y_{c}\left(k\right)$ is a function of u(k) and it is known from Equation (5) that u(k) is a function of the parameters $K_{p}\left(k\right)$, $K_{i}\left(k\right)$ and $K_{d}\left(k\right)$, then according to the chain derivative rule

(13) $$\begin{array}{c} \Delta K_{p}\left(k\right)=-\eta_{p}\frac{\partial J_{2}\left(k\right)}{\partial y_{c}^{T}\left(k\right)}\frac{\partial y_{c}\left(k\right)}{\partial u(k)}\frac{\partial u(k)}{\partial K_{p}\left(k\right)}=-\eta_{p}{\left(y_{c}\left(k\right)-{\hat{y}}_{c}\left(k\right)\right)}^{T}\frac{\partial y_{c}\left(k\right)}{\partial u(k)}e_{2}\left(k\right),\\ \Delta K_{i}\left(k\right)=-\eta_{i}\frac{\partial J_{2}\left(k\right)}{\partial y_{c}^{T}\left(k\right)}\frac{\partial y_{c}\left(k\right)}{\partial u(k)}\frac{\partial u(k)}{\partial K_{i}\left(k\right)}=-\eta_{i}{\left(y_{c}\left(k\right)-{\hat{y}}_{c}\left(k\right)\right)}^{T}\frac{\partial y_{c}\left(k\right)}{\partial u(k)}e_{1}\left(k\right),\\ \Delta K_{d}\left(k\right)=-\eta_{d}\frac{\partial J_{2}\left(k\right)}{\partial y_{c}^{T}\left(k\right)}\frac{\partial y_{c}\left(k\right)}{\partial u(k)}\frac{\partial u(k)}{\partial K_{d}\left(k\right)}=-\eta_{d}{\left(y_{c}\left(k\right)-{\hat{y}}_{c}\left(k\right)\right)}^{T}\frac{\partial y_{c}\left(k\right)}{\partial u(k)}e_{3}\left(k\right). \end{array}$$

According to Equations (8) and (9), when ${\hat{y}}_{c}\left(k\right)\approx y_{c}\left(k\right)$ after finite iterations of learning, there exists

(14) $$\begin{array}{c} \frac{\partial y_{c}\left(k\right)}{\partial u(k)}\approx \frac{\partial {\hat{y}}_{c}\left(k\right)}{\partial u(k)}=-\sum_{j=1}^{H}w_{j}\left(k\right)\zeta_{j}\left(k\right)\frac{u\left(k\right)-\varepsilon_{jM}\left(k\right)}{{\left(\sigma_{j}\left(k\right)\right)}^{2}}. \end{array}$$

At this point, it means that the RBF neural network has completed the task of tuning control parameters for the incremental PID algorithm [25].

The process of determining the initial values of control parameters in the RBF-PID algorithm using GA is similar to the process of determining $K_{p}$, $K_{i}$ and $K_{d}$ in the incremental PID algorithm using GA, and will not be repeated here.

### 2.3. Performance Metrics

We introduce the root mean square error (RMSE) [26] as a performance indicator to compare the performance of different schemes in terms of control accuracy. The formula for calculating the RMSE value of the controlled output is:

(15) $$\begin{array}{c} R\left(y_{c}\right)=\sqrt{\frac{1}{n}\sum_{k=1}^{n}{\left(r\left(k\right)-y_{c}\left(k\right)\right)}^{T}\left(r\left(k\right)-y_{c}\left(k\right)\right)}, \end{array}$$

where *k* indicates the sampling sequence number, and *n* indicates the number of sampling times. The value of $R(y_{c})$ enables us to weigh the error between the expected value and controlled value from a numerical perspective, thus allowing us to more intuitively evaluate the performance in terms of control accuracy of various control algorithms. The smaller the value of RMSE, the higher the control accuracy, and $R(y_{c})=0$ is an ideal case.

## 3. Results

The Wendling-type coupled neural mass model containing three populations is selected as the controlled object to illustrate the effectiveness of the proposed control schemes, and its mathematical expression is shown as Formula (3), where N=3. We assume that all the intrinsic parameters of the model maintain the standard values (as Table 1) except for the excitatory gain $\theta_{A}$. It should be noted here that if $\theta_{A}$ increases from the standard value 3.25 mV to the value causing spikes in the population output, then the population is called the hyperexcitable population. What we set here is that all the neural populations in the model are hyperexcited and the local excitatory gain values are completely consistent. The coupling strengths are set as $K^{12}=K^{21}=K^{23}=K^{32}=K^{31}=K^{13}=28$. Each element of the internal input vector p(t) is simulated by the Gaussian white noise with a mean of 101 and a standard deviation of 35. We set the time step to 0.001 s. The initial value of the state vector x(t) can be arbitrarily selected. David O. and Friston K. J. pointed out that neural mass models can be numerically solved using the second-order Runge–Kutta method, fourth-order Runge–Kutta method or second-order random Runge–Kutta method, and there are no essential differences between the results obtained using these methods [2]. In each simulation described below, the fourth-order Runge–Kutta method is used to solve Equation (3).

**Figure 5:**
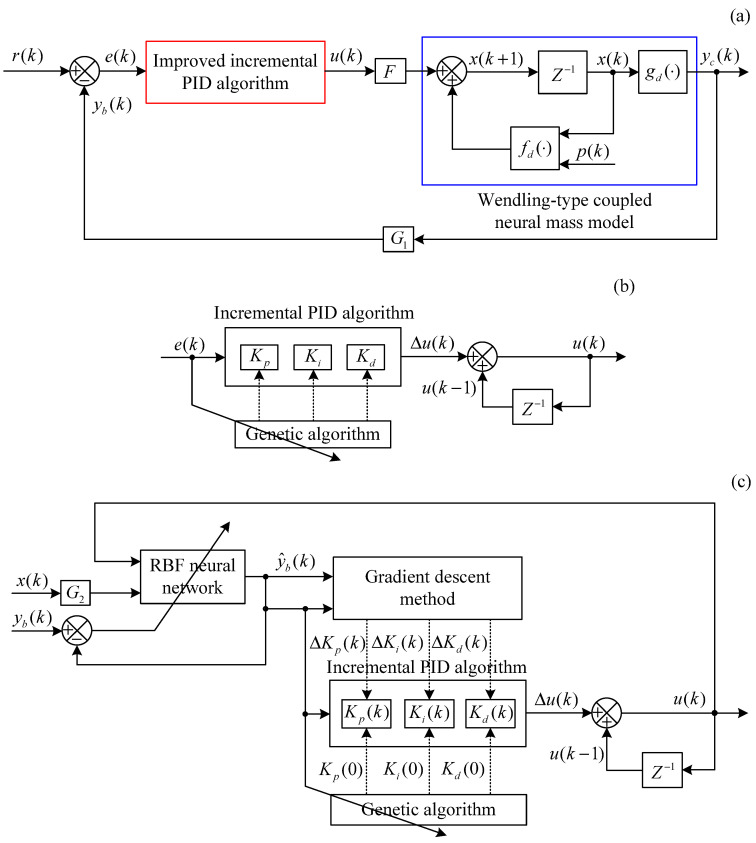
Simulation schematic diagram of the brain dynamic modulation system based on the improved incremental PID algorithms. (**a**) The overall framework of the system. (**b**) GA-PID algorithm. (**c**) GA-RBF-PID algorithm.

The simulation schematic diagram of the brain dynamic modulation system based on the improved incremental PID algorithms is shown in Figure 5. In order to highlight the differences between the two proposed control schemes, Figure 5b,c provide separate schematic diagrams of the algorithm parts. In Figure 5a, state vector is $x\left(k\right)\in R^{24}$; internal input vector is $p\left(k\right)\in R^{3}$; external control input is u(k)∈R; controlled output vector is $y\left(k\right)\in R^{3}$; let the matrix $G_{1}=\left[1\;0\;0\right]$, then feedback variable $y_{b}\left(k\right)\in R$ is the first component of the controlled output vector $y_{c}\left(k\right)$, namely $y_{b}\left(k\right)=y_{c}^{1}\left(k\right)$; r(k)∈R is the expected output; control error is $e\left(k\right)=y_{d}\left(k\right)-y_{b}\left(k\right)$; considering that all populations in the Wendling-type coupled neural mass model are hyperexcited, let the matrix $F={\left[O_{1\times 3}\;\;1\;\;O_{1\times 7}\;\;1\;\;O_{1\times 7}\;\;1\;\;O_{1\times 4}\right]}^{T}$, where $O_{m\times n}$ represents the zero matrix of m×n dimension. In Figure 5b, $K_{p}$, $K_{i}$, and $K_{d}$ are the control parameters to be determined in the GA-PID algorithm; $Z^{-1}$ represents the time delay. In Figure 5c, ${\hat{y}}_{b}\left(k\right)$ is the estimated feedback variable; $K_{p}\left(k\right)$, $K_{i}\left(k\right)$, and $K_{d}\left(k\right)$ are the control parameters to be determined in the GA-RBF-PID algorithm; $K_{p}\left(0\right)$, $K_{i}\left(0\right)$, and $K_{d}\left(0\right)$ represent the initial values of the PID parameters to be determined taking advantage of the GA; $\Delta K_{p}\left(0\right)$, $\Delta K_{i}\left(0\right)$, and $\Delta K_{d}\left(k\right)$ represent the PID parameter variations to be determined taking advantage of the RBF neural network; let $G_{2}$ = $[1\;\;O_{1\times 23};\;\;0\;\;1\;\;O_{1\times 22};\;\;O_{1\times 2}\;\;1\;\;O_{1\times 21};\;\;O_{1\times 3}\;\;1\;\;O_{1\times 20};\;\;O_{1\times 4}\;\;1\;\;O_{1\times 19};\;\;O_{1\times 5}\;\;1\;\;O_{1\times 18};$$O_{1\times 6}$$1\;\;O_{1\times 17};\;\;O_{1\times 7}\;\;1\;\;O_{1\times 16}]$, thus the *k*-th input sample data of the RBF neural network is composed of the external control input u(k) and the state variables $x_{1}^{1}\left(k\right),x_{2}^{1}\left(k\right),\cdots ,x_{8}^{1}\left(k\right)$ of the first population in the Wendling-type coupled neural mass model, namely $x_{r}\left(k\right)$ = $[x_{1}^{1}\left(k\right)$$x_{2}^{1}\left(k\right)\;\cdots \;x_{8}^{1}{\left(k\right)\;u\left(k\right)]}^{T}$. The controlled Wendling-type coupled neural mass model in Figure 5a is shown in Equation (4).

### 3.1. Results of GA Optimization Control Parameters

In this subsection, GA is used to determine the initial values $K_{p}\left(0\right)$, $K_{i}\left(0\right)$, and $K_{d}\left(0\right)$ of the control parameters for the GA-RBF-PID algorithm, as well as the control parameters $K_{p}$, $K_{i}$, and $K_{d}$ for the GA-PID algorithm, in order to ensure the control accuracy of the modulation schemes. The parameters of GA may affect the results of optimization in varying degrees, the values of which are listed as follows:

$$\begin{array}{c} \mathrm{Genetic}\;\mathrm{algebra}\;\mathrm{is}\;80,\\ \mathrm{Population}\;\mathrm{size}\;\mathrm{is}\;50,\\ \mathrm{Chromosome}\;\mathrm{coding}\;\mathrm{length}\;\mathrm{is}\;20,\\ \mathrm{Adaptive}\;\mathrm{generation}\;\mathrm{gap}\;\mathrm{is}\;0.95,\\ \mathrm{Crossover}\;\mathrm{rate}\;\mathrm{is}\;0.7,\\ \mathrm{Mutation}\;\mathrm{rate}\;\mathrm{is}\;0.01. \end{array}$$

**Figure 6:**
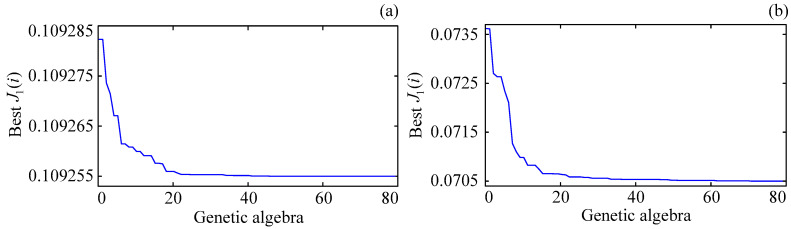
Optimal convergence curves of the GA objective function in different control schemes. (**a**) The result of the control scheme based on the GA-PID algorithm. (**b**) The result of the control scheme based on the GA-RBF-PID algorithm.

Figure 6 shows the optimal convergence curves of the GA objective function under different control schemes when the excitatory gains in the Wendling-type coupled neural mass model are all set to 3.5 mV. When the genetic algebra exceeds 40, for the brain dynamic modulation system based on the GA-PID algorithm, the optimal convergence curve of the GA objective function approximately converges to 0.11, as shown in Figure 6a; and for the brain dynamic modulation system based on the GA-RBF-PID algorithm, the optimal convergence curve of the GA objective function approximately converges to 0.07, as shown in Figure 6b. It is not difficult to see that when the genetic algebra exceeds 40, the control errors of these two schemes are already very close to the minimum values, and the potential for further optimization is reduced; in addition, the convergence value of the GA objective function is smaller in the GA-RBF-PID algorithm based control scheme, which can ensure the smaller control error.

**Figure 7:**
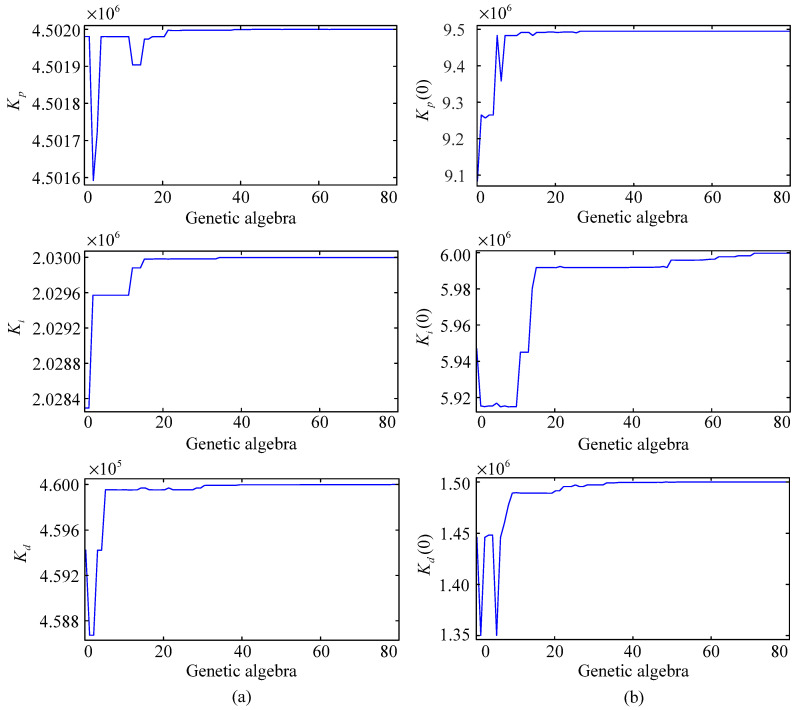
Optimization curves of the (initial) control parameters under the action of GA. (**a**) Optimization results of the control parameters in the GA-PID algorithm. (**b**) Optimization results of the initial control parameters in the GA-RBF-PID algorithm.

Under the action of GA, the optimization curves of the control parameters for the GA-PID algorithm and the initial control parameters for the GA-RBF-PID algorithm are shown in Figure 7, where Figure 7a shows the optimization results in the GA-PID algorithm, and Figure 7b shows the optimization results in the GA-RBF-PID algorithm. Combining Figure 6, it can be seen that selecting (initial) control parameters when the genetic algebra exceeds 40 can better ensure the control accuracy of the proposed schemes. In the following simulations, the control parameters for the GA-PID algorithm are taken as $K_{p}=4,501,999.99$, $K_{i}=2,029,999.77$, $K_{d}=459,999.99$; and the initial values of the control parameters for the GA-RBF-PID algorithm are taken as $K_{p}\left(0\right)=9,494,942.85$, $K_{i}\left(0\right)=5,999,712.96$, $K_{d}\left(0\right)=1,499,968.47$.

### 3.2. Analysis of Brain Dynamic Modulation Results

This subsection aims to verify whether two closed-loop control schemes designed based on the improved PID algorithms can achieve modulation target for abnormal brain dynamics simulated by the Wendling-type coupled neural mass model, and evaluate the performance of these two control schemes by comparing modulation effects and calculating the RMSEs of controlled outputs. Firstly, a set of simulation experiments with the fixed excitatory gains are used to evaluate the performance of the schemes in terms of control accuracy. We then verify whether the RBF neural network can approximate the outputs of the non-linear Wendling-type coupled neural mass model after finite iterations. Secondly, we evaluate the dynamic performance and stability of the schemes using two sets of simulation experiments with mutated excitatory gains. Only the simulation graphs of the first population in the Wendling-type coupled neural mass model are given below, because the results of populations 1, 2, and 3 are very similar.

#### 3.2.1. The Simulation Experiments with the Fixed Excitatory Gains

**Figure 8:**
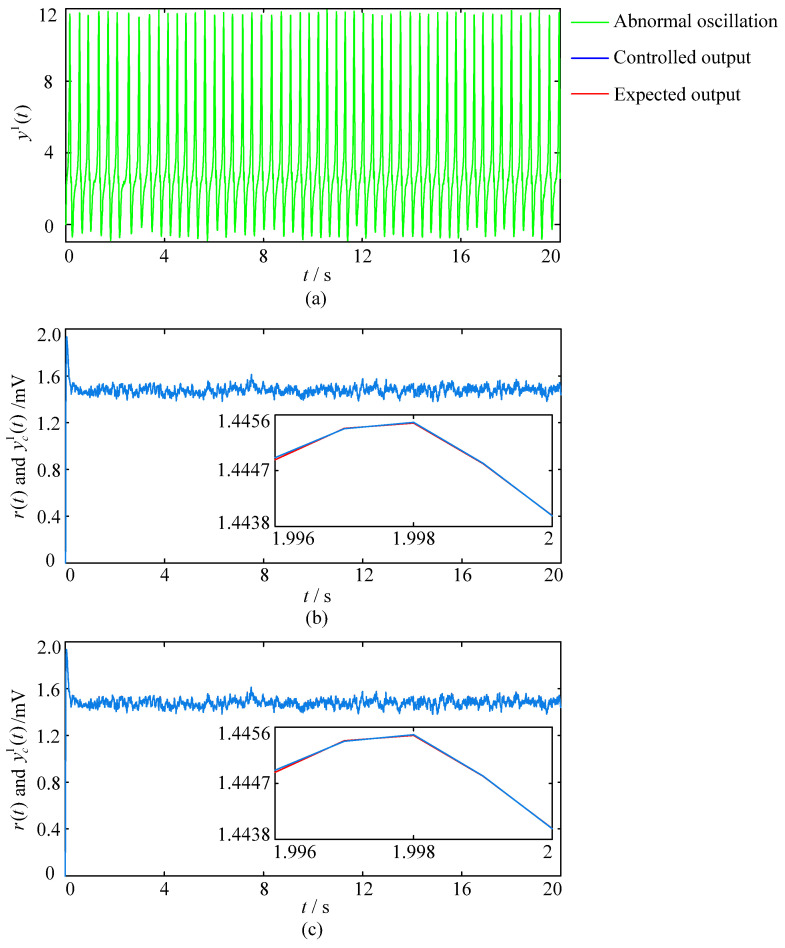
Modulation results of different control schemes with the fixed excitatory gains. (**a**) Output of the first neural population when the excitatory gain is 3.5 mV. (**b**) The modulation result of the control scheme based on the GA-PID algorithm. (**c**) The modulation result of the control scheme based on the GA-RBF-PID algorithm.

The sampling time for this set of simulation experiments is set to 20 s. Modulation results of different control schemes with the fixed excitatory gains are shown in Figure 8. Figure 8a shows the output of the population when the excitatory gains of the three hyperexcitatory neural populations in the model are all set to the constant of 3.5 mV. It can be seen that if control measures are not taken in time, the hyperexcitatory neural population 1 will generate continuous high amplitude spikes that reflect the brain dynamics under the background of onset of neuropsychiatric disorders. Figure 8b and Figure 8c respectively show the brain dynamic modulation results of control schemes based on the GA-PID and GA-RBF-PID algorithms, where the red line represents the expected output r(t), namely α-like oscillation, which reflects the brain dynamics under the background of normal brain activity; the blue line represents the controlled output $y_{c}^{1}\left(t\right)$ of the first neural population; illustrations are locally enlarged images drawn for easy observation. The blue lines in Figure 8b,c converge well to the red lines, indicating that the closed-loop control schemes based on the GA-PID and GA-RBF-PID algorithms can achieve the target of brain dynamic modulation, which demonstrate the effectiveness of these two schemes. Furthermore, it is not difficult to see from the locally enlarged images that both control schemes have high control accuracy. To more intuitively evaluate the performance of the above two control schemes in terms of control accuracy, we calculate the RMSE value of the output of population 1 within 20 s and derive $R{\left(y_{c}^{1}\right)}_{\mathrm{GA}-\mathrm{RBF}-\mathrm{PID}}$ = $2.0934\times 10^{-9}\;\mathrm{mV}$, $R{\left(y_{c}^{1}\right)}_{\mathrm{GA}-\mathrm{PID}}$ = $2.7780\times 10^{-5}\;\mathrm{mV}$. These results demonstrate the superiority of the closed-loop control scheme based on the GA-RBF-PID algorithm in terms of control accuracy when modulating brain dynamics simulated by the Wendling-type coupled neural mass model.

**Figure 9:**
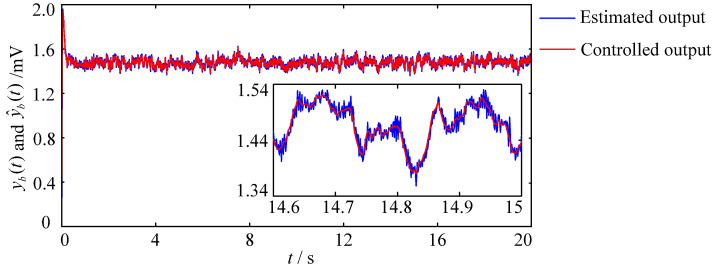
The estimation result of the controlled output based on the RBF neural network.

Figure 9 shows the result of the RBF neural network estimating the dynamics of the Wendling-type coupled neural mass model, where the red line represents the controlled output $y_{c}^{1}\left(t\right)$ of the first neural population, namely the feedback variable $y_{b}\left(t\right)$; the blue line represents the estimated feedback variable ${\hat{y}}_{b}\left(t\right)$. It can be seen that the blue line in Figure 9 converges well to the red line, proving that the RBF neural network can accurately estimate the output of the Wendling-type coupled neural mass model. Therefore, introducing the RBF neural network into the design of brain dynamic modulation schemes is beneficial in reducing the dependence of control schemes on accurate mathematical models.

#### 3.2.2. The Simulation Experiments with the Mutated Excitatory Gains

**Figure 10:**
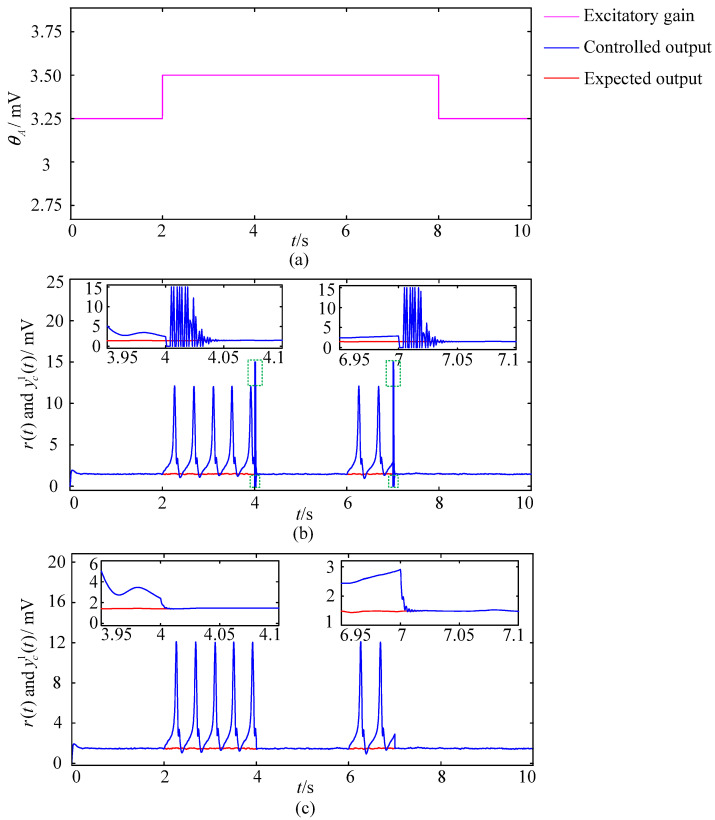
Modulation results of different control schemes with the mutated excitatory gains. (**a**) Changes in the excitatory gain. (**b**) The modulation result of the control scheme based on the GA-PID algorithm. (**c**) The modulation result of the control scheme based on the GA-RBF-PID algorithm.

The sampling time for this set of simulation experiments is set to 10 s. The excitatory gains of the three hyperexcitatory neural populations are all set to 3.25 mV during 0–2 s, and mutate to 3.5 mV at 2 s, and keep 3.5 mV invariant during 2–8 s, then return to the standard value of 3.25 mV at 8 s and maintain this value until the end of the simulation. The external control is applied during 4–6 s and 7–10 s, respectively. Modulation results of different control schemes with the mutated excitatory gains are shown in Figure 10, where the pink line reflects the changes in excitatory gains, the red line represents the expected output r(t); the blue line represents the controlled output $y_{c}^{1}\left(t\right)$ of the first neural population; illustrations are locally enlarged images drawn for easy observation. Figure 10b shows the brain dynamic modulation results of the closed-loop control scheme based on the GA-PID algorithm. The blue line in the figure produces significant overshoots (green dashed box circled part) at 4 s and 7 s when the sudden external control acts. Furthermore, from the locally enlarged image, it can be seen that there is a significant oscillation process before the blue line converges to the red line and the adjustment time is approximately 0.04 s. Figure 10c shows the modulation results obtained by applying the closed-loop control scheme based on the GA-RBF-PID algorithm. The blue line in the figure did not produce significant overshoots throughout the entire modulation process. Furthermore, from the local enlarged image, it can be seen that there are no significant oscillations at 4 s and 7 s when the external control suddenly acts, and the adjustment time is less than 0.01 s. The results show that, compared with the closed-loop control scheme based on the GA-PID algorithm, the scheme based on the GA-RBF-PID algorithm has the advantages of small overshoot and short adjustment time when modulating brain dynamics simulated by the Wendling-type coupled neural mass model, which proves the superiority of the scheme based on the GA-RBF-PID algorithm in terms of stability and dynamic performance. In view of the sudden and delayed nature of the neuropsychiatric disorders, the simulation experiments making the Wendling-type coupled neural mass model with mutated excitatory gains (which can more reasonably simulate the onset mechanism of the neuropsychiatric disorders) as the controlled object are more instructive for the optimization and innovation of brain dynamic modulation technologies.

**Figure 11:**
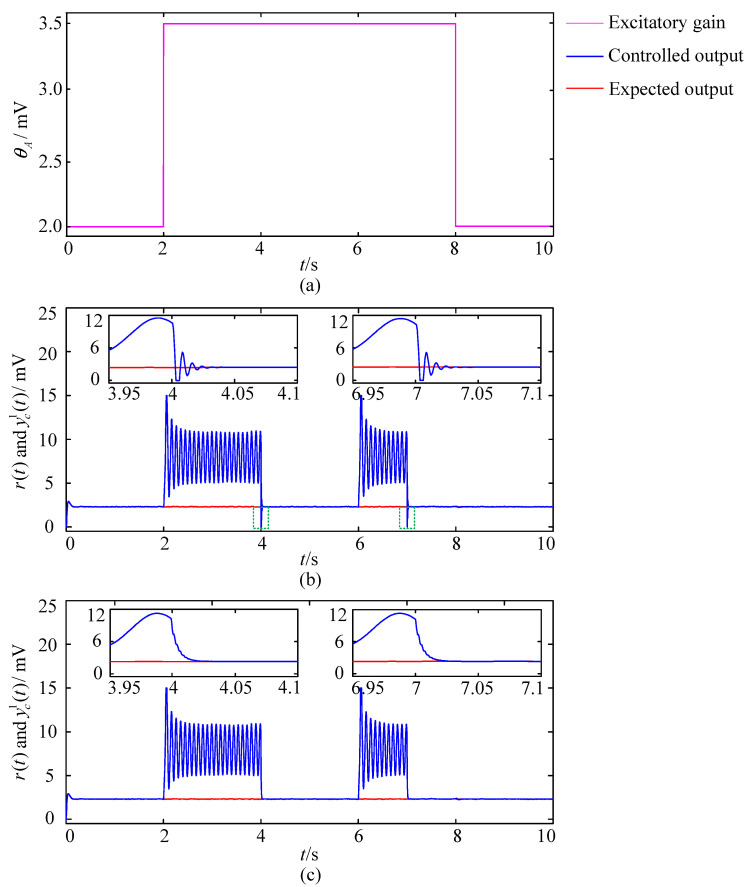
Modulation results of different control schemes with the mutated excitatory gains. (**a**) Changes in the excitatory gain. (**b**) The modulation result of the control scheme based on the GA-PID algorithm. (**c**) The modulation result of the control scheme based on the GA-RBF-PID algorithm.

In order to avoid accidental conclusions and make them more convincing, we add a set of experiments using the δ-like oscillation as the expected output. The delta-like oscillation is considered as the EEG signal when adults are in the sleep state. Liu et al. suggested that the Wendling-type coupled neural mass model can also produce the delta-like oscillation when the internal parameters take the following values [27]

$$\begin{array}{c} \theta_{A}=2\;\mathrm{mV},\;\theta_{B}=15\;\mathrm{mV},\;a=b=20\;s^{-1}\;,\;a_{d}=33\;s^{-1},\\ c_{1}=135,\;c_{2}=108,\;c_{3}=c_{4}=33.75,\;e_{0}=2.5\;s^{-1},\;v_{0}=6\;\mathrm{mV},\;r=0.56\;mV^{-1}, \end{array}$$

and each element of the internal input vector p(t) is simulated by the Gaussian white noise with a mean of 220 and a standard deviation of 22. The sampling time for this set of simulation experiments is set to 10 s. The number of populations is also N=3, and the coupling strength is still 28. The excitatory gains of the populations are all set to 2 mV during 0–2 s, mutate to 3.5 mV at 2 s, and keep 3.5 mV invariant during 2–8 s, then return to 2 mV at 8 s and maintain this value until the end of the simulation. The external control is also applied during 4–6 s and 7–10 s, respectively. Modulation results are shown in Figure 11, where the pink line in Figure 11a reflects the changes in excitatory gains, the red line represents the expected output r(t), and the blue line represents the controlled output $y_{c}^{1}\left(t\right)$ of the first neural population. The illustrations are locally enlarged images drawn for easy observation. Figure 11b shows the brain dynamic modulation results of the GA-PID algorithm based closed-loop control scheme. The blue line also generates overshoots (green dashed box circled part) at 4 s and 7 s. In addition, the locally enlarged images show the significant oscillation process before the blue line converges to the red line, and the adjustment time is approximately 0.035 s. Figure 11c shows the modulation results obtained by applying the GA-RBF-PID algorithm based closed-loop control scheme. It can be seen that the blue line did not produce significant overshoots. In addition, the locally enlarged images show that there are no significant oscillations at 4 s and 7 s when the external control suddenly acts, and the adjustment time is less than 0.025 s. That is to say, experiments using the δ-like oscillation and the α-like oscillation as expected outputs have similar results. This set of experiments further proves the superiority of the GA-RBF-PID algorithm based scheme in terms of stability and dynamic performance.

## 4. Discussion

Neural oscillations are physiological indicators that can reflect the characteristics of the brain functional states, and their generation is the core of brain dynamic behaviors [28]. More and more experimental and clinical data show that abnormal neural oscillations may be the root of the occurrence and development of a variety of neuropsychiatric disorders (also known as brain diseases) [29]. Appropriate neurostimulation can be used to modulate brain dynamics of patients with neuropsychiatric disorders to transfer abnormal neural oscillations to normal [30]. Neurostimulation is one of the most promising means to prevent and treat neuropsychiatric disorders in the medical field [31]. Given the significant individual differences and the rapid and complex process of brain dynamic changes in patients with neuropsychiatric disorders, it may be necessary to develop the optimal neurostimulation parameters (including stimulation intensity, frequency, etc.) through the vast amount of experiments. However, the limitations of understanding for the dynamic mechanisms of neural oscillations, as well as the immaturity of the medical technology and experimental conditions, may lead to irreversible brain damage and secondary injury in the direct animal and clinical trials. Computational neuroscience can analyze the general principles of brain operations from the computational perspective [32], which is beneficial for better understanding the physiological state of brain function as well as the generation and evolution mechanism of brain dysfunction. Moreover, the vast amount of experimental and clinical data is a result of the diverse nature of the brain neural system, underscoring the important role of computational neural research in the development of brain science. Previous studies have shown that control schemes developed on the basis of computational neural models can simulate changes in brain dynamics under external stimulation [33]. Making assumptions and predictions based on the obtained simulation results can help form new theoretical achievements and guide the future development direction of the “brain project”. We design brain dynamic modulation schemes on the basis of the neural mass model aiming to provide ideas and scientific basis for the optimization and innovation of the brain dynamic modulation technologies. The neural mass model with mutated excitatory gains considers the sudden and delayed nature of neuropsychiatric disorders, which can more reasonably simulate the pathogenesis of neuropsychiatric disorders. The simulation experiments making the model with mutated excitatory gains as the control object have more guiding significance for the development and improvement of early intervention and rehabilitation treatment technologies for neuropsychiatric disorders.

The mathematical model that can accurately describe the actual system serves as a bridge between clinical and fundamental theoretical research in biomedical fields [34]. In the past few decades, two kinds of complementary mathematical models have been developed to simulate brain dynamics [35,36]. Compared with the neuron model that describes the activity of brain neurons at the cellular level, the neural mass model that describes the activity of brain neuron populations at the cellular set level are both simple and more physiologically meaningful, balancing the tractability and realism, which is more conducive to a deeper insight into the generation and evolution mechanisms of neural oscillations. Like the vast majority of existing research results, this paper considers the neural mass model that contains a limited number of populations with regular connections between them. In fact, it is difficult for such models to fully describe the inherent complexity and high variability of the brain system in structure. However, the dynamics and emergence characteristics of the brain are closely related to the topological structure of the brain system [37]. Eguíluz et al. used the functional magnetic resonance imaging technology to extract functional connections in relevant brain regions when the subjects perform different tasks, and found that the connections were “scale-free” and had “small-world” characteristics [38]. The research by the Stam’s team has proven that the “small-world” is the optimal connection mode for the brain’s functional network, and the disruption of this mode is the foundation for the onset of schizophrenia [39]. They also pointed out that the general feature of Alzheimer’s disease patients is the loss of “small-world” features in the brain functional network [40]. Ponten et al. confirmed that the brain functional connectivity structure of epileptic patients exhibited significant randomization characteristics compared to normal individuals during onset [41]. Establishing network models of neural populations with the help of the tools from complex dynamic network theory or graph theory is more in line with the intricate characteristics of the brain system. Therefore, one of our future research directions is to use the proposed control schemes to modulate brain dynamics simulated by neural population network models with topological structures such as “nearest-neighbor”, “scale-free”, “small-world”, “random”, etc.

From the perspective of control theory, the design of control algorithms is another important aspect in solving the modulation problem of brain dynamics. The global optimization ability of the GA to determine the (initial) values of control parameters can ensure control accuracy, while the self-learning ability of the RBF neural network to adaptively adjust and modify control parameters online can improve the stability and dynamic performance of the control system. This paper proposes closed-loop control schemes based on the GA-PID and GA-RBF-PID algorithms to modulate the brain dynamics simulated by the Wendling-type coupled neural mass model. The introduction of the GA overcomes the difficulty of selecting control parameters for traditional incremental PID algorithm. Additionally, the introduction of the RBF neural network further enhances the adaptability of the algorithm. The simulation results demonstrate that both schemes can closely track the expected α-like and δ-like oscillations, and the closed-loop control scheme based on the GA-RBF-PID algorithm outperforms the scheme based on the GA-PID algorithm in terms of control accuracy, stability, and dynamic performance. In fact, it is possible to consider replacing GA algorithm with optimization algorithms such as simulated annealing (SA) and PSO. Similarly, it is possible to consider using algorithms that can achieve online adjustment, such as backpropagation (BP) algorithm, to replace RBF algorithm. However, so far, our understanding of similar algorithms is relatively limited. We will attempt to use other algorithms to further optimize the control scheme based on this work, striving to design a brain dynamics closed-loop modulation strategy with superior performance. The potential benefits of applying closed-loop control in brain dynamic modulation strategies include ensuring therapeutic effectiveness, improving response by providing intermittent or minimal stimulation, minimizing side effects, minimizing damage, minimizing power consumption, and extending the battery life of implanted devices [5]. The basic requirement for achieving brain dynamic modulation through the PID control is that the feedback signals used to construct the control law should accurately reflect the functional state of the brain tissue. However, the neural oscillations recorded by technologies such as EEG, magnetoencephalogram, functional magnetic resonance imaging, and local field potential may become inaccurate due to amplifier noise, as well as uncertain factors such as electrode resistance and capacitance in recording devices, leading to unexpected control behavior in the brain dynamic modulation system [42]. For the neural mass models whose dynamics are affected by measurement noise, if the demand for the control accuracy is not so high and the main modulation target is to suppress spikes, various non-linear Kalman filter (such as UKF, cubature Kalman filter, etc.) algorithms are the preferred algorithms for designing modulation schemes of brain dynamics [13,14]. Another important feature of non-linear Kalman filter algorithms is to identify unknown parameters of the model by combining it with the augmented state vector method. Previous studies have shown that certain brain regions become hyperexcitatory when their excitatory level exceed the self-regulation range of the brain system, leading to abnormal neural oscillations [43]. If not controlled in a timely manner, abnormal neural oscillations may spread to other regions through functional connections, which lead to the onset of neuropsychiatric disorders. That is to say, hyperexcitatory brain regions may be the source of lesions for neuropsychiatric disorders. The recognition of hyperexcitatory brain regions helps to locate the source of lesions, which is an important step in suppressing the onset of neuropsychiatric disorders and reducing brain damage. At present, the common method for simulating the onset mechanism of such neuropsychiatric disorders is to increase the excitatory gains of populations in neural mass models [43]. Identifying unknown excitatory gains in neural mass models is of great significance for the prevention and treatment of neuropsychiatric disorders. Moreover, existing research has confirmed that in some cases, the goal of brain dynamic modulation can be achieved by controlling only the hyperexcitatory neural populations [13]. From the perspective of reducing the cost of control and easy implementation of control, it is equally significant to find the hyperexcitatory neural populations in advance. Therefore, our another future research direction is to use the control scheme based on the improved non-linear Kalman filter algorithms to regulate the brain dynamics simulated by neural population network models with parameter uncertainty and output affected by measurement noise.

## 5. Conclusions

This paper proposes the closed-loop control frameworks based on the improved incremental PID algorithms to modulate brain dynamics simulated by the Wendling-type coupled neural mass model. The introduction of GA overcomes the disadvantage that the selection of control parameters for traditional incremental PID algorithm depends on the designer’s experience, and thus ensures the high control accuracy of the modulation schemes. RBF neural networks can approximate the dynamics of the non-linear neural mass models to improve the adaptability of the modulation schemes. The simulation results not only confirm the effectiveness of the proposed schemes, but also indicate that the closed-loop control scheme based on the GA-RBF-PID algorithm outperforms the scheme based on the GA-PID algorithm in terms of control accuracy—especially stability and dynamic performance. We hope to demonstrate the modulation schemes proposed in existing and future studies in real EEG data in the near future.

## Figures and Tables

**Table 1:** Physiological meanings and standard values of the model parameters.

Parameter	Physiological Meaning	Standard Value
$\theta_{A},\theta_{B}$	average gains of excitatory and inhibitory synaptic	$\theta_{A}=3.25\;\mathrm{mV},\theta_{B}=22\;\mathrm{mV}$
a,b	membrane transfer and dendritic tree average time delay	$a=100\;s^{-1},b=50\;s^{-1}$
$a_{d}$	average contact time between neural populations	$a_{d}$ = 33 $s^{-1}$
$c_{1},c_{2}$	average synaptic connections in the excitatory feedback loop	$c_{1}$ = 135, $c_{2}$ = 108
$c_{3},c_{4}$	average synaptic connections in the inhibitory feedback loop	$c_{3}=c_{4}$ = 33.75
	$e_{0}$ represents the maximum firing rate	$e_{0}$ = 2.5 $s^{-1}$
	*r* represents bending degree of the sigmoid function	*r* = 0.56 ${\mathrm{mV}}^{-1}$
S(·)	$v_{0}$ is the postsynaptic potential corresponding to firing rate	$v_{0}$ = 6 mV
	*v* represents the presynaptic average membrane potential	no standard value

## Data Availability

The authors confirm that the data supporting the findings of this study are available within the article and its Supplementary Materials.

## Supplementary Materials

The following supporting information can be downloaded at: https://www.mdpi.com/article/10.3390/e25111544/s1.
