# Supplementary material for: Design of Closed-Loop Control Schemes Based on the GA-PID and GA-RBF-PID Algorithms for Brain Dynamic Modulation
Source: Entropy (Basel). 2023 Nov 15;25(11):1544. doi: 10.3390/e25111544 (PMC10670460; doi:10.3390/e25111544)
Supplement: Supplementary file 1 [file entropy-25-01544-s001.zip › GA_RBF_PID/gatbx/DOC/GATBXA0.PDF]

Genetic Algorithm TOOLBOX

For Use with MATLAB®

Andrew Chipperfield
Peter Fleming
Hartmut Pohlheim
Carlos Fonseca

Department of
AUTOMATIC
CONTROL
and SYSTEMS
ENGINEERING
UNIVERSITY of SHEFFIELD

Version 1.2

User's Guide

Acknowledgements

The production of this Toolbox was made possible by a UK SERC grant on "Genetic Algorithms in Control Systems Engineering" (GR/J17920). Many thanks are due to Hartmut Pohlheim, a visiting researcher from the Technical University Ilmenau, Germany, for the support for real-valued genetic algorithms and his hard work in coding and revising many of the routines in this Toolbox. Thanks are also due to Carlos Fonseca for providing the initial prototype for this Toolbox.

Table of Contents

1 Tutorial 1-1

Installation 1-2

An Overview of Genetic Algorithms 1-3
- What are Genetic Algorithms 1-3
- GAs versus Traditional Methods 1-5

Major Elements of the Genetic Algorithm 1-6
- Population Representation and Initialisation 1-6
- The Objective and Fitness Functions 1-8
- Selection 1-9
  - Roulette Wheel Selection Methods 1-10
  - Stochastic Universal Sampling 1-12
- Crossover 1-12
  - Multi-point Crossover 1-12
  - Uniform Crossover 1-13
  - Other Crossover Operators 1-14
  - Intermediate Recombination 1-14
  - Line Recombination 1-15
  - Discussion 1-15
- Mutation 1-16
- Reinsertion 1-18
- Termination of the GA 1-18

Data Structures 1-20
- Chromosomes 1-20
- Phenotypes 1-20
- Objective Function Values 1-21
- Fitness Values 1-22

Support for Multiple Populations 1-23

Examples 1-26
- The Simple GA 1-26
- A Multi-population GA 1-30
- Demonstration Scripts 1-36

References 1-37

2 Reference 2-1
