# Supplementary material for: Design of Closed-Loop Control Schemes Based on the GA-PID and GA-RBF-PID Algorithms for Brain Dynamic Modulation
Source: Entropy (Basel). 2023 Nov 15;25(11):1544. doi: 10.3390/e25111544 (PMC10670460; doi:10.3390/e25111544)
Supplement: Supplementary file 1 [file entropy-25-01544-s001.zip › GA_RBF_PID/gatbx/DOC/GATBXA1.PDF]

1 Tutorial

MATLAB has a wide variety of functions useful to the genetic algorithm practitioner and those wishing to experiment with the genetic algorithm for the first time. Given the versatility of MATLAB's high-level language, problems can be coded in m-files in a fraction of the time that it would take to create C or Fortran programs for the same purpose. Couple this with MATLAB's advanced data analysis, visualisation tools and special purpose application domain toolboxes and the user is presented with a uniform environment with which to explore the potential of genetic algorithms.

The Genetic Algorithm Toolbox uses MATLAB matrix functions to build a set of versatile tools for implementing a wide range of genetic algorithm methods. The Genetic Algorithm Toolbox is a collection of routines, written mostly in m-files, which implement the most important functions in genetic algorithms.

Installation

Instructions for installing the Genetic Algorithm Toolbox can be found in the MATLAB installation instructions. It is recommended that the files for this toolbox are stored in a directory named genetic off the main matlab/toolbox directory.

A number of demonstrations are available. A single-population binary-coded genetic algorithm to solve a numerical optimization problem is implemented in the m-file `sga.m`. The demonstration m-file `mpga.m` implements a real-valued multi-population genetic algorithm to solve a dynamic control problem. Both of these demonstration m-files are discussed in detail in the *Examples* Section.

Additionally, a set of test functions, drawn from the genetic algorithm literature, are supplied in a separate directory, `test_fns`, from the Genetic Algorithm Toolbox functions. A brief description of these test functions is given at the end of the *Examples* Section. A further document describes the implementation and use of these functions.

An Overview of Genetic Algorithms

In this Section we give a tutorial introduction to the basic Genetic Algorithm (GA) and outline the procedures for solving problems using the GA.

What are Genetic Algorithms?

The GA is a stochastic global search method that mimics the metaphor of natural biological evolution. GAs operate on a population of potential solutions applying the principle of survival of the fittest to produce (hopefully) better and better approximations to a solution. At each generation, a new set of approximations is created by the process of selecting individuals according to their level of fitness in the problem domain and breeding them together using operators borrowed from natural genetics. This process leads to the evolution of populations of individuals that are better suited to their environment than the individuals that they were created from, just as in natural adaptation.

Individuals, or current approximations, are encoded as strings, *chromosomes*, composed over some alphabet(s), so that the *genotypes* (chromosome values) are uniquely mapped onto the decision variable (*phenotypic*) domain. The most commonly used representation in GAs is the binary alphabet {0, 1} although other representations can be used, e.g. ternary, integer, real-valued etc. For example, a problem with two variables, x_1 and x_2, may be mapped onto the chromosome structure in the following way:

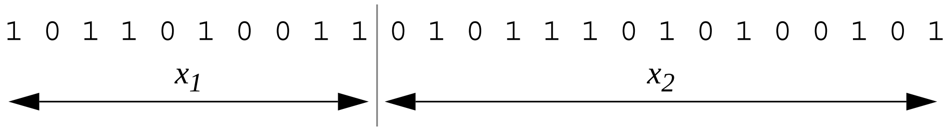

where x_1 is encoded with 10 bits and x_2 with 15 bits, possibly reflecting the level of accuracy or range of the individual decision variables. Examining the chromosome string in isolation yields no information about the problem we are trying to solve. It is only with the decoding of the chromosome into its phenotypic values that any meaning can be applied to the representation. However, as described below, the search process will operate on this encoding of the decision variables, rather than the decision variables themselves, except, of course, where real-valued genes are used.

Having decoded the chromosome representation into the decision variable domain, it is possible to assess the performance, or *fitness*, of individual members of a population. This is done through an objective function that characterises an individual's performance in the problem domain. In the natural world, this would be an individual's ability to survive in its present environment. Thus, the objective

function establishes the basis for selection of pairs of individuals that will be mated together during reproduction.

During the reproduction phase, each individual is assigned a fitness value derived from its raw performance measure given by the objective function. This value is used in the selection to bias towards more fit individuals. Highly fit individuals, relative to the whole population, have a high probability of being selected for mating whereas less fit individuals have a correspondingly low probability of being selected.

Once the individuals have been assigned a fitness value, they can be chosen from the population, with a probability according to their relative fitness, and recombined to produce the next generation. Genetic operators manipulate the characters (genes) of the chromosomes directly, using the assumption that certain individual's gene codes, on average, produce fitter individuals. The recombination operator is used to exchange genetic information between pairs, or larger groups, of individuals. The simplest recombination operator is that of single-point crossover.

Consider the two parent binary strings:

P_1 = 1 0 0 1 0 1 1 0, and

P_2 = 1 0 1 1 1 0 0 0.

If an integer position, *i*, is selected uniformly at random between 1 and the string length, *l*, minus one [1, *l*-1], and the genetic information exchanged between the individuals about this point, then two new offspring strings are produced. The two offspring below are produced when the crossover point $i = 5$ is selected,

O_1 = 1 0 0 1 0 0 0 0, and

O_2 = 1 0 1 1 1 1 1 0.

This crossover operation is not necessarily performed on all strings in the population. Instead, it is applied with a probability *Px* when the pairs are chosen for breeding. A further genetic operator, called mutation, is then applied to the new chromosomes, again with a set probability, *Pm*. Mutation causes the individual genetic representation to be changed according to some probabilistic rule. In the binary string representation, mutation will cause a single bit to change its state, $0 \Rightarrow 1$ or $1 \Rightarrow 0$. So, for example, mutating the fourth bit of O_1 leads to the new string,

O_{1m} = 1 0 0 0 0 0 0 0.

Mutation is generally considered to be a background operator that ensures that the probability of searching a particular subspace of the problem space is never zero.

This has the effect of tending to inhibit the possibility of converging to a local optimum, rather than the global optimum.

After recombination and mutation, the individual strings are then, if necessary, decoded, the objective function evaluated, a fitness value assigned to each individual and individuals selected for mating according to their fitness, and so the process continues through subsequent generations. In this way, the average performance of individuals in a population is expected to increase, as good individuals are preserved and bred with one another and the less fit individuals die out. The GA is terminated when some criteria are satisfied, e.g. a certain number of generations, a mean deviation in the population, or when a particular point in the search space is encountered.

GAs versus Traditional Methods

From the above discussion, it can be seen that the GA differs substantially from more traditional search and optimization methods. The four most significant differences are:

- GAs search a population of points in parallel, not a single point.
- GAs do not require derivative information or other auxiliary knowledge; only the objective function and corresponding fitness levels influence the directions of search.
- GAs use probabilistic transition rules, not deterministic ones.
- GAs work on an encoding of the parameter set rather than the parameter set itself (except in where real-valued individuals are used).

It is important to note that the GA provides a number of potential solutions to a given problem and the choice of final solution is left to the user. In cases where a particular problem does not have one individual solution, for example a family of Pareto-optimal solutions, as is the case in multiobjective optimization and scheduling problems, then the GA is potentially useful for identifying these alternative solutions simultaneously.

Major Elements of the Genetic Algorithm

The simple genetic algorithm (SGA) is described by Goldberg [1] and is used here to illustrate the basic components of the GA. A pseudo-code outline of the SGA is shown in Fig. 1. The population at time *t* is represented by the time-dependent variable *P*, with the initial population of random estimates being *P(0)*. Using this outline of a GA, the remainder of this Section describes the major elements of the GA.

```
procedure GA
begin
        t = 0;
        initialize P(t);
        evaluate P(t);
        while not finished do
        begin
                t = t + 1;
                select P(t) from P(t-1);
                reproduce pairs in P(t);
                evaluate P(t);
        end
end.
```

Figure 1: A Simple Genetic Algorithm

Population Representation and Initialisation

GAs operate on a number of potential solutions, called a population, consisting of some encoding of the parameter set simultaneously. Typically, a population is composed of between 30 and 100 individuals, although, a variant called the micro GA uses very small populations, ~10 individuals, with a restrictive reproduction and replacement strategy in an attempt to reach real-time execution [2].

The most commonly used representation of chromosomes in the GA is that of the single-level binary string. Here, each decision variable in the parameter set is encoded as a binary string and these are concatenated to form a chromosome. The use of Gray coding has been advocated as a method of overcoming the hidden representational bias in conventional binary representation as the Hamming distance between adjacent values is constant [3]. Empirical evidence of Caruana and Schaffer [4] suggests that large Hamming distances in the representational mapping between adjacent values, as is the case in the standard binary representation, can result in the search process being deceived or unable to

efficiently locate the global minimum. A further approach of Schmitendorgf *et-al* [5], is the use of logarithmic scaling in the conversion of binary-coded chromosomes to their real phenotypic values. Although the precision of the parameter values is possibly less consistent over the desired range, in problems where the spread of feasible parameters is unknown, a larger search space may be covered with the same number of bits than a linear mapping scheme allowing the computational burden of exploring unknown search spaces to be reduced to a more manageable level.

Whilst binary-coded GAs are most commonly used, there is an increasing interest in alternative encoding strategies, such as integer and real-valued representations. For some problem domains, it is argued that the binary representation is in fact deceptive in that it obscures the nature of the search [6]. In the subset selection problem [7], for example, the use of an integer representation and look-up tables provides a convenient and natural way of expressing the mapping from representation to problem domain.

The use of real-valued genes in GAs is claimed by Wright [8] to offer a number of advantages in numerical function optimization over binary encodings. Efficiency of the GA is increased as there is no need to convert chromosomes to phenotypes before each function evaluation; less memory is required as efficient floating-point internal computer representations can be used directly; there is no loss in precision by discretisation to binary or other values; and there is greater freedom to use different genetic operators. The use of real-valued encodings is described in detail by Michalewicz [9] and in the literature on Evolution Strategies (see, for example, [10]).

Having decided on the representation, the first step in the SGA is to create an initial population. This is usually achieved by generating the required number of individuals using a random number generator that uniformly distributes numbers in the desired range. For example, with a binary population of N_{ind} individuals whose chromosomes are L_{ind} bits long, $N_{ind} \times L_{ind}$ random numbers uniformly distributed from the set $\{0, 1\}$ would be produced.

A variation is the *extended random initialisation* procedure of Bramlette [6] whereby a number of random initialisations are tried for each individual and the one with the best performance is chosen for the initial population. Other users of GAs have seeded the initial population with some individuals that are known to be in the vicinity of the global minimum (see, for example, [11] and [12]). This approach is, of course, only applicable if the nature of the problem is well understood beforehand or if the GA is used in conjunction with a knowledge based system.

The GA Toolbox supports binary, integer and floating-point chromosome representations. Binary and integer populations may be initialised using the Toolbox function to create binary populations, `crtbp`. An additional function, `crtbase`, is provided that builds a vector describing the integer representation

used. Real-valued populations may be initialised with the function `crtrp`. Conversion between binary strings and real values is provided by the routine `bs2rv` that supports the use of Gray codes and logarithmic scaling.

The Objective and Fitness Functions

The objective function is used to provide a measure of how individuals have performed in the problem domain. In the case of a minimization problem, the most fit individuals will have the lowest numerical value of the associated objective function. This raw measure of fitness is usually only used as an intermediate stage in determining the relative performance of individuals in a GA. Another function, the *fitness function*, is normally used to transform the objective function value into a measure of relative fitness [13], thus:

$$F(x) = g(f(x))$$

where f is the objective function, g transforms the value of the objective function to a non-negative number and F is the resulting relative fitness. This mapping is always necessary when the objective function is to be minimized as the lower objective function values correspond to fitter individuals. In many cases, the fitness function value corresponds to the number of offspring that an individual can expect to produce in the next generation. A commonly used transformation is that of proportional fitness assignment (see, for example, [1]). The individual fitness, $F(x_i)$, of each individual is computed as the individual's raw performance, $f(x_i)$, relative to the whole population, i.e.,

$$F(x_i) = \frac{f(x_i)}{\sum_{i=1}^{N_{ind}} f(x_i)},$$

where N_{ind} is the population size and x_i is the phenotypic value of individual i. Whilst this fitness assignment ensures that each individual has a probability of reproducing according to its relative fitness, it fails to account for negative objective function values.

A linear transformation which offsets the objective function [1] is often used prior to fitness assignment, such that,

$$F(x) = af(x) + b$$

where a is a positive scaling factor if the optimization is maximizing and negative if we are minimizing. The offset b is used to ensure that the resulting fitness values are non-negative.

The linear scaling and offsetting outlined above is, however, susceptible to rapid convergence. The *selection* algorithm (see below) selects individuals for reproduction on the basis of their relative fitness. Using linear scaling, the expected number of offspring is approximately proportional to that individuals performance. As there is no constraint on an individual's performance in a given generation, highly fit individuals in early generations can dominate the reproduction causing rapid convergence to possibly sub-optimal solutions. Similarly, if there is little deviation in the population, then scaling provides only a small bias towards the most fit individuals.

Baker [14] suggests that by limiting the reproductive range, so that no individuals generate an excessive number of offspring, prevents premature convergence. Here, individuals are assigned a fitness according to their rank in the population rather than their raw performance. One variable, *MAX*, is used to determine the bias, or *selective pressure*, towards the most fit individuals and the fitness of the others is determined by the following rules:

- $MIN = 2.0 - MAX$
- $INC = 2.0 \times (MAX - 1.0) / N_{ind}$
- $LOW = INC / 2.0$

where *MIN* is the lower bound, *INC* is the difference between the fitness of adjacent individuals and *LOW* is the expected number of trials (number of times selected) of the least fit individual. *MAX* is typically chosen in the interval [1.1, 2.0]. Hence, for a population size of $N_{ind} = 40$ and $MAX = 1.1$, we obtain $MIN = 0.9$, $INC = 0.05$ and $LOW = 0.025$. The fitness of individuals in the population may also be calculated directly as,

$$F(x_i) = 2 - MAX + 2(MAX - 1)\frac{x_i - 1}{N_{ind} - 1},$$

where x_i is the position in the ordered population of individual *i*.

Objective functions must be created by the user, although a number of example m-files are supplied with the Toolbox that implement common test functions. These objective functions all have the filename prefix `obj`. The Toolbox supports both linear and non-linear ranking methods, `ranking`, and includes a simple linear scaling function, `scaling`, for completeness. It should be noted that the linear scaling function is not suitable for use with objective functions that return negative fitness values.

Selection

Selection is the process of determining the number of times, or *trials*, a particular individual is chosen for reproduction and, thus, the number of offspring that an

individual will produce. The selection of individuals can be viewed as two separate processes:

1) determination of the number of trials an individual can expect to receive, and

2) conversion of the expected number of trials into a discrete number of offspring.

The first part is concerned with the transformation of raw fitness values into a real-valued expectation of an individual's probability to reproduce and is dealt with in the previous subsection as fitness assignment. The second part is the probabilistic selection of individuals for reproduction based on the fitness of individuals relative to one another and is sometimes known as *sampling*. The remainder of this subsection will review some of the more popular selection methods in current usage.

Baker [15] presented three measures of performance for selection algorithms, *bias*, *spread* and *efficiency*. Bias is defined as the absolute difference between an individual's actual and expected selection probability. Optimal zero bias is therefore achieved when an individual's selection probability equals its expected number of trials.

Spread is the range in the possible number of trials that an individual may achieve. If *f(i)* is the actual number of trials that individual *i* receives, then the "minimum spread" is the smallest spread that theoretically permits zero bias, i.e.

$$f(i) \in \left\{ \lfloor et(i) \rfloor, \lceil et(i) \rceil \right\}$$

where *et(i)* is the expected number of trials of individual *i*, $\lfloor et(i) \rfloor$ is the floor of et(i) and $\lceil et(i) \rceil$ is the ceil. Thus, while bias is an indication of accuracy, the spread of a selection method measures its consistency.

The desire for efficient selection methods is motivated by the need to maintain a GAs overall time complexity. It has been shown in the literature that the other phases of a GA (excluding the actual objective function evaluations) are $O(L_{ind}.N_{ind})$ or better time complexity, where L_{ind} is the length of an individual and N_{ind} is the population size. The selection algorithm should thus achieve zero bias whilst maintaining a minimum spread and not contributing to an increased time complexity of the GA.

Roulette Wheel Selection Methods

Many selection techniques employ a "roulette wheel" mechanism to probabilistically select individuals based on some measure of their performance. A real-valued interval, *Sum*, is determined as either the sum of the individuals'

expected selection probabilities or the sum of the raw fitness values over all the individuals in the current population. Individuals are then mapped one-to-one into contiguous intervals in the range [0, *Sum*]. The size of each individual interval corresponds to the fitness value of the associated individual. For example, in Fig. 2 the circumference of the roulette wheel is the sum of all six individual's fitness values. Individual 5 is the most fit individual and occupies the largest interval, whereas individuals 6 and 4 are the least fit and have correspondingly smaller intervals within the roulette wheel. To select an individual, a random number is generated in the interval [0, *Sum*] and the individual whose segment spans the random number is selected. This process is repeated until the desired number of individuals have been selected.

The basic roulette wheel selection method is stochastic sampling with replacement (SSR). Here, the segment size and selection probability remain the same throughout the selection phase and individuals are selected according to the procedure outlined above. SSR gives zero bias but a potentially unlimited spread. Any individual with a segment size > 0 could entirely fill the next population.

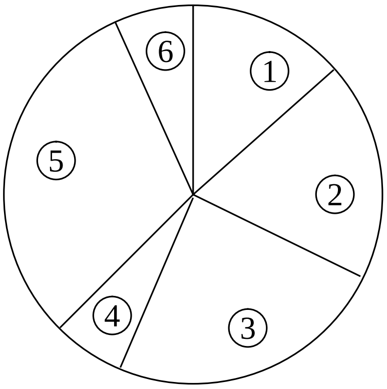

Figure 2: Roulette Wheel Selection

Stochastic sampling with partial replacement (SSPR) extends upon SSR by resizing an individual's segment if it is selected. Each time an individual is selected, the size of its segment is reduced by 1.0. If the segment size becomes negative, then it is set to 0.0. This provides an upper bound on the spread of $\lceil et(i) \rceil$. However, the lower bound is zero and the bias is higher than that of SSR.

Remainder sampling methods involve two distinct phases. In the integral phase, individuals are selected deterministically according to the integer part of their expected trials. The remaining individuals are then selected probabilistically from the fractional part of the individuals expected values. Remainder stochastic sampling with replacement (RSSR) uses roulette wheel selection to sample the individual not assigned deterministically. During the roulette wheel selection phase, individual's fractional parts remain unchanged and, thus, compete for

selection between “spins”. RSSR provides zero bias and the spread is lower bounded. The upper bound is limited only by the number of fractionally assigned samples and the size of the integral part of an individual. For example, any individual with a fractional part > 0 could win all the samples during the fractional phase. Remainder stochastic sampling without replacement (RSSWR) sets the fractional part of an individual’s expected values to zero if it is sampled during the fractional phase. This gives RSSWR minimum spread, although this selection method is biased in favour of smaller fractions.

Stochastic Universal Sampling

Stochastic universal sampling (SUS) is a single-phase sampling algorithm with minimum spread and zero bias. Instead of the single selection pointer employed in roulette wheel methods, SUS uses N equally spaced pointers, where N is the number of selections required. The population is shuffled randomly and a single random number in the range [0 *Sum*/*N*] is generated, *ptr*. The N individuals are then chosen by generating the N pointers spaced by 1, [*ptr*, *ptr*+1, ..., *ptr*+*N*-1], and selecting the individuals whose fitnesses span the positions of the pointers. An individual is thus guaranteed to be selected a minimum of $\lfloor et(i) \rfloor$ times and no more than $\lceil et(i) \rceil$, thus achieving minimum spread. In addition, as individuals are selected entirely on their position in the population, SUS has zero bias.

The roulette wheel selection methods can all be implemented as O(NlogN) although SUS is a simpler algorithm and has time complexity O(N). The Toolbox supplies a stochastic universal sampling function, `sus`, and the stochastic sampling with replacement algorithm, `rws`.

Crossover (Recombination)

The basic operator for producing new chromosomes in the GA is that of crossover. Like its counterpart in nature, crossover produces new individuals that have some parts of both parent’s genetic material. The simplest form of crossover is that of single-point crossover, described in the Overview of GAs. In this Section, a number of variations on crossover are described and discussed and the relative merits of each reviewed.

Multi-point Crossover

For multi-point crossover, m crossover positions, $k_i \in \{1, 2, \ldots, l-1\}$, where k_i are the crossover points and l is the length of the chromosome, are chosen at random with no duplicates and sorted into ascending order. Then, the bits between successive crossover points are exchanged between the two parents to produce two

new offspring. The section between the first allele position and the first crossover point is not exchanged between individuals. This process is illustrated in Fig. 3.

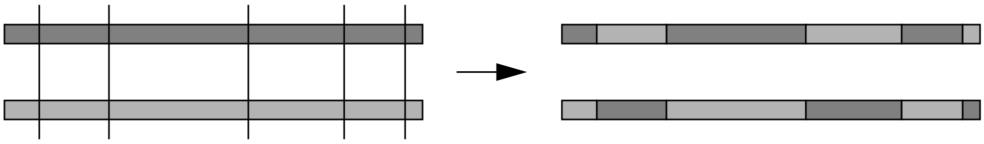

Figure 3: Multi-point Crossover (m=5)

The idea behind multi-point, and indeed many of the variations on the crossover operator, is that the parts of the chromosome representation that contribute to the most to the performance of a particular individual may not necessarily be contained in adjacent substrings [16]. Further, the disruptive nature of multi-point crossover appears to encourage the exploration of the search space, rather than favoring the convergence to highly fit individuals early in the search, thus making the search more robust [17].

Uniform Crossover

Single and multi-point crossover define cross points as places between loci where a chromosome can be split. Uniform crossover [18] generalises this scheme to make every locus a potential crossover point. A crossover mask, the same length as the chromosome structures is created at random and the parity of the bits in the mask indicates which parent will supply the offspring with which bits. Consider the following two parents, crossover mask and resulting offspring:

P_1 = 1 0 1 1 0 0 0 1 1 1

P_2 = 0 0 0 1 1 1 1 0 0 0

Mask = 0 0 1 1 0 0 1 1 0 0

O_1 = 0 0 1 1 1 1 0 1 0 0

O_2 = 1 0 0 1 0 0 1 0 1 1

Here, the first offspring, O_1, is produced by taking the bit from P_1 if the corresponding mask bit is 1 or the bit from P_2 if the corresponding mask bit is 0. Offspring O_2 is created using the inverse of the mask or, equivalently, swapping P_1 and P_2.

Uniform crossover, like multi-point crossover, has been claimed to reduce the bias associated with the length of the binary representation used and the particular coding for a given parameter set. This helps to overcome the bias in single-point crossover towards short substrings without requiring precise understanding of the

significance of individual bits in the chromosome representation. Spears and De Jong [19] have demonstrated how uniform crossover may be parameterised by applying a probability to the swapping of bits. This extra parameter can be used to control the amount of disruption during recombination without introducing a bias towards the length of the representation used. When uniform crossover is used with real-valued alleles, it is usually referred to as *discrete recombination*.

Other Crossover Operators

A related crossover operator is that of *shuffle* [20]. A single cross-point is selected, but before the bits are exchanged, they are randomly shuffled in both parents. After recombination, the bits in the offspring are unshuffled. This too removes positional bias as the bits are randomly reassigned each time crossover is performed.

The *reduced surrogate* operator [16] constrains crossover to always produce new individuals wherever possible. Usually, this is implemented by restricting the location of crossover points such that crossover points only occur where gene values differ.

Intermediate Recombination

Given a real-valued encoding of the chromosome structure, intermediate recombination is a method of producing new phenotypes around and between the values of the parents phenotypes [21]. Offspring are produced according to the rule,

$$O_1 = P_1 \times \alpha (P_2 - P_1),$$

where α is a scaling factor chosen uniformly at random over some interval, typically [-0.25, 1.25] and P_1 and P_2 are the parent chromosomes (see, for example, [21]). Each variable in the offspring is the result of combining the variables in the parents according to the above expression with a new α chosen for each pair of parent genes. In geometric terms, intermediate recombination is

capable of producing new variables within a slightly larger hypercube than that defined by the parents but constrained by the range of α. as shown in Fig 4.

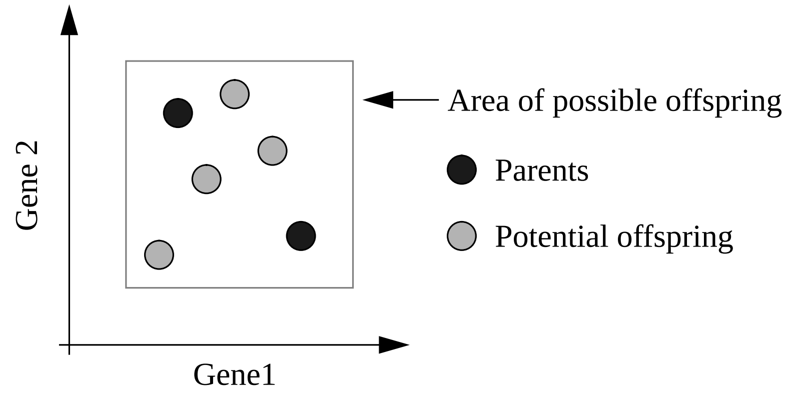

Figure 4: Geometric Effect of Intermediate Recombination

Line Recombination

Line recombination [21] is similar to intermediate recombination, except that only one value of α is used in the recombination. Fig. 5 shows how line recombination can generate any point on the line defined by the parents within the limits of the perturbation, α, for a recombination in two variables.

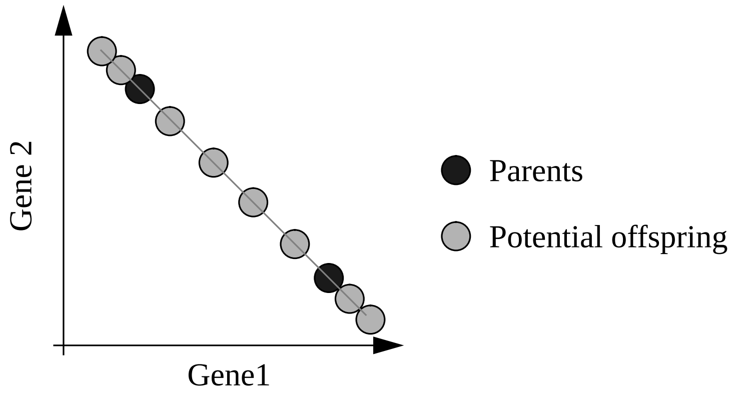

Figure 5: Geometric Effect of Line Recombination

Discussion

The binary operators discussed in this Section have all, to some extent, used disruption in the representation to help improve exploration during recombination. Whilst these operators may be used with real-valued populations, the resulting

changes in the genetic material after recombination would not extend to the actual values of the decision variables, although offspring may, of course, contain genes from either parent. The intermediate and line recombination operators overcome this limitation by acting on the decision variables themselves. Like uniform crossover, the real-valued operators may also be parameterised to provide a control over the level of disruption introduced into offspring. For discrete-valued representations, variations on the recombination operators may be used that ensure that only valid values are produced as a result of crossover [22].

The GA Toolbox provides a number of crossover routines incorporating most of the methods described above. Single-point, double-point and shuffle crossover are implemented in the Toolbox functions `xovsp`, `xovdp` and `xovsh`, respectively, and can operate on any chromosome representation. Reduced surrogate crossover is supported with both single-point, `xovsprs`, and double-point, `xovdprs`, crossover and with shuffle crossover, `xovshrs`. A further general multi-point crossover routine, `xovmp`, is also provided. To support real-valued chromosome representations, discrete, intermediate and line recombination operators are also included. The discrete recombination operator, `recdis`, performs crossover on real-valued individuals in a similar manner to the uniform crossover operators. Line and intermediate recombination are supported by the functions `reclin` and `recint` respectively. A high-level entry function to all of the crossover operators is provided by the function `recombin`.

Mutation

In natural evolution, mutation is a random process where one allele of a gene is replaced by another to produce a new genetic structure. In GAs, mutation is randomly applied with low probability, typically in the range 0.001 and 0.01, and modifies elements in the chromosomes. Usually considered as a background operator, the role of mutation is often seen as providing a guarantee that the probability of searching any given string will never be zero and acting as a safety net to recover good genetic material that may be lost through the action of selection and crossover [1].

The effect of mutation on a binary string is illustrated in Fig. 6 for a 10-bit chromosome representing a real value decoded over the interval [0, 10] using both standard and Gray coding and a mutation point of 3 in the binary string. Here, binary mutation flips the value of the bit at the loci selected to be the mutation point. Given that mutation is generally applied uniformly to an entire population of

strings, it is possible that a given binary string may be mutated at more than one point.

mutation point →		binary	Gray
Original string -	0 0 0 1 1 0 0 0 1 0	0.9659	0.6634
Mutated string -	0 0 1 1 1 0 0 0 1 0	2.2146	1.8439

Figure 6: Binary Mutation

With non-binary representations, mutation is achieved by either perturbing the gene values or random selection of new values within the allowed range. Wright [8] and Janikow and Michalewicz [23] demonstrate how real-coded GAs may take advantage of higher mutation rates than binary-coded GAs, increasing the level of possible exploration of the search space without adversely affecting the convergence characteristics. Indeed, Tate and Smith [24] argue that for codings more complex than binary, high mutation rates can be both desirable and necessary and show how, for a complex combinatorial optimization problem, high mutation rates and non-binary coding yielded significantly better solutions than the normal approach.

Many variations on the mutation operator have been proposed. For example, biasing the mutation towards individuals with lower fitness values to increase the exploration in the search without losing information from the fitter individuals [25] or parameterising the mutation such that the mutation rate decreases with the population convergence [26]. Mühlenbein [21] has introduced a mutation operator for the real-coded GA that uses a non-linear term for the distribution of the range of mutation applied to gene values. It is claimed that by biasing mutation towards smaller changes in gene values, mutation can be used in conjunction with recombination as a foreground search process. Other mutation operations include that of *trade mutation* [7], whereby the contribution of individual genes in a chromosome is used to direct mutation towards weaker terms, and *reorder mutation* [7], that swaps the positions of bits or genes to increase diversity in the decision variable space.

Binary and integer mutation are provided in the Toolbox by the function `mut`. Real-valued mutation is available using the function `mutbga`. A high-level entry function to the mutation operators is provided by the function `mutate`.

Reinsertion

Once a new population has been produced by selection and recombination of individuals from the old population, the fitness of the individuals in the new population may be determined. If fewer individuals are produced by recombination than the size of the original population, then the fractional difference between the new and old population sizes is termed a generation gap [27]. In the case where the number of new individuals produced at each generation is one or two, the GA is said to be steady-state [28] or incremental [29]. If one or more of the most fit individuals is deterministically allowed to propagate through successive generations then the GA is said to use an *elitist strategy.*

To maintain the size of the original population, the new individuals have to be reinserted into the old population. Similarly, if not all the new individuals are to be used at each generation or if more offspring are generated than the size of the old population then a reinsertion scheme must be used to determine which individuals are to exist in the new population. An important feature of not creating more offspring than the current population size at each generation is that the generational computational time is reduced, most dramatically in the case of the steady-state GA, and that the memory requirements are smaller as fewer new individuals need to be stored while offspring are produced.

When selecting which members of the old population should be replaced the most apparent strategy is to replace the least fit members deterministically. However, in studies, Fogarty [30] has shown that no significant difference in convergence characteristics was found when the individuals selected for replacement where chosen with inverse proportional selection or deterministically as the least fit. He further asserts that replacing the least fit members effectively implements an elitist strategy as the most fit will probabilistically survive through successive generations. Indeed, the most successful replacement scheme was one that selected the oldest members of a population for replacement. This is reported as being more in keeping with generational reproduction as every member of the population will, at some time, be replaced. Thus, for an individual to survive successive generations, it must be sufficiently fit to ensure propagation into future generations.

The GA Toolbox provides a function for reinserting individuals into the population after recombination, `reins`. Optional input parameters allow the use of either uniform random or fitness-based reinsertion. Additionally, this routine can also be selected to reinsert fewer offspring than those produced at recombination.

Termination of the GA

Because the GA is a stochastic search method, it is difficult to formally specify convergence criteria. As the fitness of a population may remain static for a number of generations before a superior individual is found, the application of

conventional termination criteria becomes problematic. A common practice is to terminate the GA after a prespecified number of generations and then test the quality of the best members of the population against the problem definition. If no acceptable solutions are found, the GA may be restarted or a fresh search initiated.

Data Structures

MATLAB essentially supports only one data type, a rectangular matrix of real or complex numeric elements. The main data structures in the Genetic Algorithm toolbox are:

- chromosomes
- phenotypes
- objective function values
- fitness values

These data structures are discussed in the following subsections.

Chromosomes

The chromosome data structure stores an entire population in a single matrix of size `Nind` × `Lind`, where `Nind` is the number of individuals in the population and `Lind` is the length of the genotypic representation of those individuals. Each row corresponds to an individual's genotype, consisting of base-*n*, typically binary, values.

An example of the chromosome data structure is shown below.

$$\texttt{Chrom} = \begin{bmatrix} g_{1,1} & g_{1,2} & g_{1,3} & \cdots & g_{1,\texttt{Lind}} \\ g_{2,1} & g_{2,2} & g_{2,3} & \cdots & g_{2,\texttt{Lind}} \\ g_{3,1} & g_{3,2} & g_{3,3} & \cdots & g_{3,\texttt{Lind}} \\ . & . & . & \cdots & . \\ g_{\text{Nind},1} & g_{\text{Nind},2} & g_{\texttt{Nind},3} & \cdots & g_{\texttt{Nind},\texttt{Lind}} \end{bmatrix} \begin{matrix} \text{individual 1} \\ \text{individual 2} \\ \text{individual 3} \\ . \\ \text{individual } \texttt{Nind} \end{matrix}$$

This data representation does not force a structure on the chromosome structure, only requiring that all chromosomes are of equal length. Thus, structured populations or populations with varying genotypic bases may be used in the Genetic Algorithm Toolbox provided that a suitable decoding function, mapping chromosomes onto phenotypes, is employed. The role of the decoding function is described below.

Phenotypes

The decision variables, or phenotypes, in the genetic algorithm are obtained by applying some mapping from the chromosome representation into the decision variable space. Here, each string contained in the chromosome structure decodes

to a row vector of order Nvar, according to the number of dimensions in the search space and corresponding to the decision variable vector value.

The decision variables are stored in a numerical matrix of size $\mathtt{Nind} \times \mathtt{Nvar}$. Again, each row corresponds to a particular individual's phenotype. An example of the phenotype data structure is given below, where DECODE is used to represent an arbitrary function, possibly from the GA Toolbox, mapping the genotypes onto the phenotypes.

```
Phen = DECODE(Chrom)  % Map Genotype to Phenotype
```

$$
= \begin{bmatrix} x_{1,1} & x_{1,2} & x_{1,3} & \cdots & x_{1,\mathtt{Nvar}} \\ x_{2,1} & x_{2,2} & x_{2,3} & \cdots & x_{2,\mathtt{Nvar}} \\ x_{3,1} & x_{3,2} & x_{3,3} & \cdots & x_{3,\mathtt{Nvar}} \\ . & . & . & \cdots & . \\ x_{\mathtt{Nind},1} & x_{\mathtt{Nind},2} & x_{\mathtt{Nind},3} & \cdots & x_{\mathtt{Nind},\mathtt{Nvar}} \end{bmatrix} \begin{matrix} \text{individual 1} \\ \text{individual 2} \\ \text{individual 3} \\ . \\ \text{individual } \mathtt{Nind} \end{matrix}
$$

The actual mapping between the chromosome representation and their phenotypic values depends upon the DECODE function used. It is perfectly feasible using this representation to have vectors of decision variables of different types. For example, it is possible to mix integer, real-valued and alphanumeric decision variables in the same Phen data structure.

Objective function values

An objective function is used to evaluate the performance of the phenotypes in the problem domain. Objective function values can be scalar or, in the case of multiobjective problems, vectorial. Note that objective function values are not necessarily the same as the fitness values.

Objective function values are stored in a numerical matrix of size $\mathtt{Nind} \times \mathtt{Nobj}$, where Nobj is the number of objectives. Each row corresponds to a particular individual's objective vector. An example of the objective function values data structure is shown below, with OBJFUN representing an arbitrary objective function.

```
ObjV = OBJFUN(Phen)  % Objective Function
```

$$= \begin{bmatrix} y_{1,1} & y_{1,2} & y_{1,3} & \cdots & y_{1,\mathtt{Nvar}} \\ y_{2,1} & y_{2,2} & y_{2,3} & \cdots & y_{2,\mathtt{Nvar}} \\ y_{3,1} & y_{3,2} & y_{3,3} & \cdots & y_{3,\mathtt{Nvar}} \\ . & . & . & \cdots & . \\ y_{\mathtt{Nind},1} & y_{\mathtt{Nind},2} & y_{\mathtt{Nind},3} & \cdots & y_{\mathtt{Nind},\mathtt{Nvar}} \end{bmatrix} \begin{matrix} \text{individual 1} \\ \text{individual 2} \\ \text{individual 3} \\ . \\ \text{individual } \mathtt{Nind} \end{matrix}$$

Fitness values

Fitness values are derived from objective function values through a scaling or ranking function. Fitnesses are non-negative scalars and are stored in column vectors of length `Nind`, an example of which is shown below. Again, FITNESS is an arbitrary fitness function.

```
Fitn = FITNESS(ObjV)  % Fitness Function
```

$$= \begin{bmatrix} f_1 \\ f_2 \\ f_3 \\ . \\ f_{\mathtt{Nind}} \end{bmatrix} \begin{matrix} \text{individual 1} \\ \text{individual 2} \\ \text{individual 3} \\ . \\ \text{individual } \mathtt{Nind} \end{matrix}$$

Note that for multiobjective functions, the fitness of a particular individual is a function of a vector of objective function values. Multiobjective problems are characterised by having no single unique solution, but a family of equally fit solutions with different values of decision variables. Care should therefore be taken to adopt some mechanism to ensure that the population is able to evolve the set of Pareto optimal solutions, for example by using fitness sharing [31] in the selection method. Although not supported in this version of the Genetic Algorithm Toolbox, it is planned that multiobjective search will be implemented in future versions.

Support for Multiple Populations

The GA Toolbox provides support for multiple subpopulations through the use of high-level genetic operator functions and a routine for exchanging individuals between subpopulations. In the literature, the use of multiple populations has been shown, in most cases, to improve the quality of the results obtained using GAs compared to the single population GA (see, for example, [32] and [33]).

The GA Toolbox supports the use of a single population divided into a number of subpopulations or *demes* by modifying the use of data structures such that subpopulations are stored in contiguous blocks within a single matrix. For example, the chromosome data structure, `Chrom`, composed of `SUBPOP` subpopulations each of length `N` individuals is stored as:

$$\mathtt{Chrom} = \begin{bmatrix} \mathtt{Ind}_1\,\mathtt{SubPop}_1 \\ \mathtt{Ind}_2\,\mathtt{SubPop}_1 \\ \ldots \\ \mathtt{Ind}_N\,\mathtt{SubPop}_1 \\ \mathtt{Ind}_1\,\mathtt{SubPop}_2 \\ \mathtt{Ind}_2\,\mathtt{SubPop}_2 \\ \ldots \\ \mathtt{Ind}_N\,\mathtt{SubPop}_2 \\ \ldots \\ \mathtt{Ind}_1\,\mathtt{SubPop}_{\mathtt{SUBPOP}} \\ \mathtt{Ind}_2\,\mathtt{SubPop}_{\mathtt{SUBPOP}} \\ \ldots \\ \mathtt{Ind}_N\,\mathtt{SubPop}_{\mathtt{SUBPOP}} \end{bmatrix}.$$

This is known as the *Migration*, or *Island*, model [34]. Each subpopulation is evolved over generations by a traditional GA and from time to time individuals migrate from one subpopulation to another. The amount of migration of individuals and the pattern of that migration determines how much genetic diversity can occur.

To allow the Toolbox routines to operate independently on subpopulations, a number of high-level entry functions are provided that accept an optional argument that determines the number of subpopulations contained in a data structure. The low-level routines are then called independently, in turn, with each subpopulation

to perform functions such as selection, crossover and reinsertion. These high-level functions are listed in the Table below.

SUBPOPULATION SUPPORT FUNCTIONS	
`mutate`	mutation operators
`recombin`	crossover and recombination operators
`reins`	uniform random and fitness-based reinsertion
`select`	independent subpopulation selection

Note: As currently implemented, all subpopulations must be of equal size.

The transfer of individuals between subpopulations is implemented in the Toolbox function `migrate`. A single scalar is used to determine the amount of migration of individuals from one subpopulation to another. Thus, given a population comprised of a number of subpopulations, the same number of individuals will always be transferred from a subpopulation as the number it will receive from another subpopulation. A second parameter to the function `migrate` controls the manner in which individuals are selected for migration, either uniformly or according to fitness. Uniform selection picks individuals for migration and replaces individuals in a subpopulation with immigrants in a random manner. Fitness-based migration selects individuals according to their fitness level, the most fit individuals being the ones selected for migration, and replaces individuals in a subpopulation uniformly at random.

A further parameter specifies the population topology over which migration will take place. Fig. 7 shows the most basic migration paths implemented in `migrate`, the ring topology. Here individuals are transferred between directionally adjacent subpopulations. For example, individuals from subpopulation 6 migrate only to subpopulation 1 and individuals from subpopulation 1 only migrate to subpopulation 2.

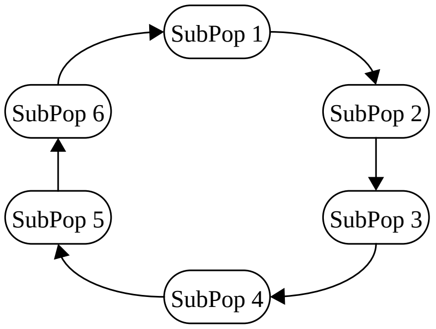

Figure 7: Ring Migration Topology

A similar strategy to the ring topology is the neighbourhood migration of Fig.8. Like the ring topology, migration is made only between nearest neighbours, however, migration may occur in either direction between subpopulations. For each subpopulation, the possible immigrants are determined, according to the desired selection method, from adjacent subpopulations and a final selection made from this pool of individuals. This ensures that individuals will not migrate from a subpopulation to the same subpopulation.

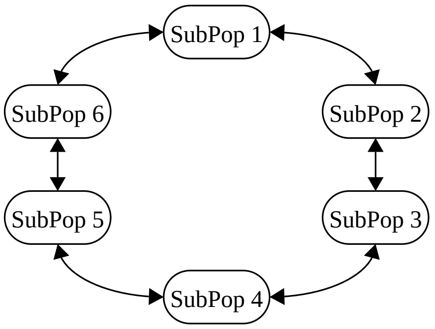

Figure 8: Neighbourhood Migration Topology

The most general migration strategy supported by `migrate` is that of unrestricted migration, Fig. 9. Here, individuals may migrate from any subpopulation to another. For each subpopulation, a pool of potential immigrants is constructed from the other subpopulations. The individual migrants are then determined according to the appropriate selection strategy.

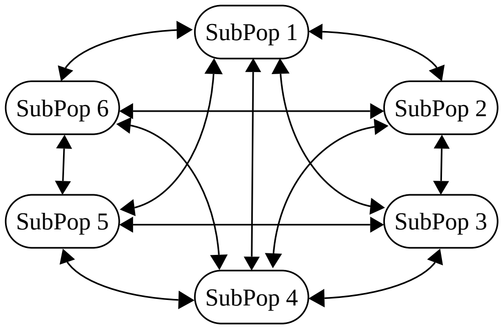

Figure 9: Unrestricted Migration Topology

An example of a GA with multiple subpopulations is considered in the *Examples* Section.

Examples

This Section contains two detailed examples using the GA Toolbox to solve optimization problems:

- A simple binary GA to solve De Jong's first test function [13].
- A real-valued multi-population GA to solve the Harvest problem [9].

The Simple GA

This example demonstrates how a simple GA can be constructed using routines from the GA Toolbox to solve an optimization problem. The objective function to be minimized is an extended version of De Jong's first test function [13]:

$$f_1(x) = \sum_{i=1}^{n} x_i^2, \qquad -512 \le x_i \le 512$$

where n defines the number of dimensions of the problem. For this example, we choose $n = 20$. The minimum of this function is, of course, located at $x_i = 0$.

The computational element of the MATLAB objective function is encapsulated in the code segment below.

```
function ObjVal = objfun1( Phen )
ObjVal = sum((Phen .* Phen)')';
```

An m-file implementing this objective function, `objfun1`, is included with the GA Toolbox software.

Having written an m-file for the objective function, the GA code may now be constructed. This can be done directly from the MATLAB command line, in a script file or as a MATLAB function. Fig. 10 shows an outline of the script file `sga` supplied with the toolbox that implements a simple GA to solve this problem.

The first five lines describe the major variables of the GA. The number of individuals is set to `NIND = 40` and the number of generations `MAXGEN = 300`. The number of variables used is `NVAR = 20` and each variable uses a 20 bit representation, `PRECI = 20`. This example uses a generation gap, `GGAP = 0.9`, and fitness-based reinsertion to implement an elitist strategy whereby the four most fit individuals always propagate through to successive generations. Thus, 36 (`NIND` × `GGAP`) new individuals are produced at each generation.

```
NIND = 40;                  % Number of individuals
MAXGEN = 300;               % Maximum no. of generations
NVAR = 20;                  % No. of variables
PRECI = 20;                 % Precision of variables
GGAP = 0.9;                 % Generation gap

% Build field descriptor
FieldD = [rep([PRECI],[1,NVAR]);...
  rep([-512;512],[1,NVAR]); rep([1;0;1;1],[1,NVAR])];

% Initialise population
Chrom = crtbp(NIND, NVAR*PRECI);

gen = 0;                    % Counter

% Evaluate initial population
ObjV = objfun1(bs2rv(Chrom,FieldD));

% Generational loop
while gen < MAXGEN,

    % Assign fitness values to entire population
    FitnV = ranking(ObjV);

    % Select individuals for breeding
    SelCh = select('sus', Chrom, FitnV, GGAP);

    % Recombine individuals (crossover)
    SelCh = recombin('xovsp',SelCh,0.7);

    % Apply mutation
    SelCh = mut(SelCh);

    % Evaluate offspring, call objective function
    ObjVSel = objfun1(bs2rv(SelCh,FieldD));

    % Reinsert offspring into population
    [Chrom ObjV]=reins(Chrom,SelCh,1,1,ObjV,ObjVSel);

    % Increment counter
    gen = gen+1;

end
```

Figure 10: The Simple GA in MATLAB

The field descriptor is constructed using the matrix replication function, `rep`, to build the matrix, `FieldD`, describing the chromosomes' representation and interpretation. In this case, `FieldD` describes 20 variables, each Gray coded using 20 bits over the interval [-512, 512]. An initial population is then created with the function `crtbp` thus,

```
Chrom = crtbp(NIND, NVAR*PRECI);
```

producing a matrix, `Chrom`, of `NIND` uniformly distributed random binary strings of length `NVAR` × `PRECI`.

The generation counter, `gen`, is set to zero. The following line then converts the binary strings to real-values using the function `bs2rv` and evaluates the objective function, `objfun1`, for all of the individuals in the initial population as shown below.

```
ObjV = objfun1(bs2rv(Chrom, FieldD));
```

The function `bs2rv` converts the binary strings in the matrix `Chrom` to real-values according to the field descriptor, `FieldD`, and returns a matrix of real-valued phenotypes. The return value matrix of `bs2rv` is then passed directly as the input argument to the objective function, `objfun1`, and the resulting objective function values are returned in the matrix `ObjV`. The GA then enters the generational `while` loop.

The first step in the generational loop is the assignment of fitness values to the individuals. In this example, rank-based fitness assignment is used as shown below,

```
FitnV = ranking(ObjV);
```

Here, the objective function values, ObjV, are passed to the Toolbox function `ranking` with no other arguments. The default setting for the ranking algorithm assume a selective pressure of 2 and linear ranking, giving the most fit individual a fitness value of 2 and the least fit individual a fitness value of 0. Note that the ranking algorithm assumes that the objective function is to be **minimised**. The resulting fitness values are returned in the vector `FitnV`.

The selection stage uses the high-level function `select` to call the low-level stochastic universal sampling routine, `sus`, as follows,

```
SelCh = select('sus', Chrom, FitnV, GGAP);
```

After selection, `SelCh` contains `GGAP` × `NIND` individuals from the original population `Chrom`. These individuals are now recombined using the high-level function `recombin` as shown below.

```
SelCh = recombin('xovsp', SelCh, 0.7);
```

`recombin` takes the individuals selected for reproduction, `SelCh`, and uses the single-point crossover routine, `xovsp`, to perform crossover with probability, *Px* = 0.7. The individuals in the input matrix `SelCh` are ordered such that individuals in odd numbered positions are crossed with the individual in the adjacent even numbered position. If the number of individuals in `SelCh` is odd then the last

individual is always returned uncrossed. The offspring produced by this crossover are returned in the same matrix, `SelCh`. The actual crossover routine used may be changed by supplying a different function name in the string passed to `recombin`.

Having produced a set of offspring, mutation may now be applied using the mutation function `mut`:

```
SelCh = mut(SelCh);
```

Again, the offspring are returned in the matrix `SelCh`. As no probability of mutation has been specified in the function call, the default value of *Pm* = 0.7/*Lind* = 0.0017, where *Lind* is the length of an individual, is assumed.

The objective function values for the offspring, `ObjVSel`, may now be calculated thus:

```
ObjVSel = objfun1(bs2rv(SelCh, FieldD));
```

Because we have used a generation gap, the number of offspring is less than the size of the population. Therefore, we must reinsert the offspring into the current population. This is achieved using the reinsertion function, `reins`, as follows:

```
[Chrom,ObjV]=reins(Chrom, SelCh,1,1,ObjV,ObjVSel);
```

Here, `Chrom` and `SelCh` are matrices containing the original population and the resulting offspring. The two occurrences of the numeral `1` indicate that a single population is used and that fitness-based reinsertion be applied. Fitness-based reinsertion replaces the least fit members of `Chrom` with the individuals in `SelCh`. The objective function values of the original population, `ObjV`, are thus required as a parameter to `reins`. In addition, so that the objective function values of the new population can be returned without having to re-evaluate the objective function for the entire population, the objective values of the offspring, `ObjVSel`, are also supplied. `reins` returns the new population with the offspring inserted, `Chrom`, and the objective function values for this population, `ObjV`.

Finally, the generational counter, `gen`, is incremented. The GA iterates around the loop until `gen = MAXGEN`, in this case 300, and then terminates. The results of the genetic optimization are contained in the matrix ObjV and the values of the decision variables may be obtained by:

```
Phen = bs2rv(Chrom, FieldD);
```

A Multi-population GA

This example shows how functions from the GA Toolbox may be used to implement a real-valued, multi-population GA. A MATLAB m-file script `mpga`, supplied with the Toolbox, implements the code described in this subsection. The objective function chosen is that of the harvest problem [9] which is a one-dimensional equation of growth:

$$x_{k+1} = a \cdot x_k - u_k,$$

with one equality constraint,

$$x_0 = x_N,$$

where x_0 is the initial condition of the state, a is a scalar constant, and $x_k \in R$ and $u_k \in R^+$ are the state and nonnegative control respectively. The objective function is defined as:

$$J = \max \sum_{k=0}^{N-1} \sqrt{u_k},$$

where N is the number of control steps over which the problem is to be solved. An m-file implementing this objective function, `objharv`, is supplied with the GA Toolbox software. Note that as this is a maximisation problem and the Toolbox routines are implemented to minimise, the objective function, `objharv`, multiplies J by -1 to produce a minimisation problem. The initial condition is set to $x_0 = 100$ and the scalar is chosen as $a = 1.1$. Additionally, the exact optimal solution for this problem can be determined analytically as:

$$J^* = \sqrt{\frac{x_0 (a^N - 1)^2}{a^{N-1}(a-1)}}.$$

The number of control steps for this problem is $N = 20$, thus, `NVAR = 20` decision variables will be used, one for each control input, u_k. The decision variables are bounded in the range `RANGE = [0, 200]`, limiting the maximum control input, at any time-step, to 200. The field descriptor, `FieldD`, describing the decision variables may be constructed using the matrix replication function, `rep`, thus:

```
NVAR = 20;
RANGE = [0; 200];
FieldD = rep(RANGE,[1,NVAR]);
```

The parameters for the GA may be specified using MATLAB variables. For this example the following parameters are defined:

```
% Define GA Parameters
GGAP = 0.8;          % Generation gap
XOVR = 1;            % Crossover rate
MUTR = 1/NVAR;       % Mutation rate
MAXGEN = 1200;       % Maximum no. of generations
INSR = 0.9;          % Insertion rate
SUBPOP = 8;          % No. of subpopulations
MIGR = 0.2;          % Migration rate
MIGGEN = 20;         % No. of gens / migration
NIND = 20;           % No. of individuals / subpop
```

As well as the conventional GA parameters, such as generation gap (`GGAP`) and crossover rate (`XOVR`), a number of other parameters associated with multi-population GAs are defined. Here, `INSR = 0.9` specifies that only 90% of the individuals produced at each generation are reinserted into the population, `SUBPOP = 8` subpopulations are to be used with a migration rate of `MIGR = 0.2`, or 20%, between subpopulations and migration occurring at every `MIGGEN = 20` generations. Each subpopulation contains `NIND = 20` individuals.

The functions used by the script-file are specified using MATLAB strings:

```
% Specify other functions as strings
SEL_F = 'sus';       % Name of selection function
XOV_F = 'recdis';    % Name of recombination fun.
MUT_F = 'mutbga';    % Name of mutation function
OBJ_F = 'objharv';   % Name of objective function
```

Because we are using discrete recombination, `recdis`, for the breeding of offspring, the crossover rate is not used and, hence `XOVR = 1` above.

The initial population is created using the function `crtrp` and the generation counter, `gen`, set to zero:

```
Chrom = crtrp(SUBPOP*NIND,FieldD);
gen = 0;
```

This will consist of `SUBPOP` × `NIND` individuals with individual decision variables chosen uniformly at random in the range specified by `FieldD`. The `Chrom` matrix contains all of the subpopulations and the objective function values for all the individuals in all the subpopulations may be calculated directly,

```
ObjV = feval(OBJ_F, Chrom);
```

using the MATLAB `feval` command. `feval` performs function evaluation taking the first input argument, in this case the name of our objective function, `objharv`, contained in `OBJ_F`, as the function to be evaluated and calls that function with all the remaining parameters as its input arguments. In this case, the function call is:

```
ObjV = objharv(Chrom);
```

As a real-valued coding is used, there is no need to convert the chromosomes into a phenotypic representation. Like the previous example, the GA now enters a generational `while` loop.

The MATLAB code for the generational loop of the multi-population GA is shown in Fig. 11 below.

```
% Generational loop
while gen < MAXGEN,

    % Fitness assignment to whole population
    FitnV = ranking(ObjV,2,SUBPOP);

    % Select individuals from population
    SelCh = select(SEL_F, Chrom, FitnV, GGAP, SUBPOP);

    % Recombine selected individuals
    SelCh=recombin(XOV_F, SelCh, XOVR, SUBPOP);

    % Mutate offspring
    SelCh = mutate(MUT_F,SelCh,FieldD,[MUTR],SUBPOP);

    % Calculate objective function for offsprings
    ObjVOff = feval(OBJ_F,SelCh);

    % Insert best offspring replacing worst parents
    [Chrom, ObjV] = reins(Chrom, SelCh, SUBPOP, ...
          [1 INSR], ObjV, ObjVOff);

    % Increment counter
    gen=gen+1;

    % Migrate individuals between subpopulations
    if (rem(gen,MIGGEN) == 0)
       [Chrom, ObjV] = ...
           migrate(Chrom, SUBPOP, [MIGR, 1, 1], ObjV);
    end

end
```

Figure 11: Generational Loop of a Multipopulation GA

The first step of the generational loop is the assignment of fitness values to individuals:

```
FitnV = ranking(ObjV, 2, SUBPOP);
```

Because we are using multiple subpopulations, `ranking` requires us to specify the selective pressure required, here we use a selective pressure of 2, and the number of subpopulations, `SUBPOP`. Each subpopulation's individuals' objective values in `ObjV` are ranked separately and the resulting sets of fitness values returned in the vector `FitnV`.

Within each subpopulation, individuals are selected for breeding independently using the high-level selection function, `select`:

```
SelCh = select(SEL_F, Chrom, FitnV, GGAP, SUBPOP);
```

`select` calls the low-level selection function, `SEL_F = 'sus'` for each subpopulation and builds the matrix `SelCh` containing all the pairs of individuals to be recombined. Like the previous example, the generation gap, `GGAP = 0.8`, means that 0.8 × 20 = 16, `GGAP` × `NIND`, individuals are selected from each subpopulation. Thus, `SelCh` contains a total of `GGAP` × `NIND` × `SUBPOP = 128` individuals.

In a similar manner, the high-level recombination function, `recombin`, is used to recombine the pairs of individuals within each subpopulation of `SelCh`:

```
SelCh = recombin(XOV_F, SelCh, XOVR, SUBPOP);
```

The recombination function, `XOV_F = 'recdis'`, performs discrete recombination between pairs of individuals for each subpopulation. As discrete recombination does not require the specification of a conventional crossover rate, the variable `XOVR = 1.0` is used only for compatibility.

The offspring are now mutated:

```
SelCh = mutate(MUT_F,SelCh,FieldD,MUTR,SUBPOP);
```

Here, the breeder genetic algorithm mutation function, `MUT_F = 'mutbga'`, is called using the high-level mutation routine, `mutate`, with a mutation rate of `MUTR = 1/NIND = 0.05`. The breeder genetic algorithm mutation function requires the field descriptor, `FieldD`, so that the result of mutation will not produce values outside the bounds of the decision variables.

The objective values of all the offspring, `ObjVOff` may now be calculated, again using `feval`:

```
ObjVOff = feval(OBJ_F, SelCh);
```

Offspring may now be reinserted into the appropriate subpopulations:

```
[Chrom, ObjV] = reins(Chrom, SelCh, SUBPOP,...
                      [1, INSR], ObjV, ObjVOff);
```

Fitness-based reinsertion is used, but the addition of the extra parameter to the fourth argument of `reins` specifies an insertion rate of `INSR = 0.9`. This means that for each subpopulation the least-fit 10% of the offspring are not reinserted.

Individuals in Multi-population GAs migrate between populations at some interval. The Toolbox routine `migrate` is used to swap individuals between subpopulations according to some migration strategy. In this example, at every `MIGGEN = 20` generations, migration takes place between subpopulations.

```
% Migration between subpopulations
if(rem(gen, MIGGEN) == 0)
    [Chrom, ObjV] = migrate(Chrom, SUBPOP, ...
                            [MIGR, 1, 1], ObjV);
end
```

Here, the most fit 20%, `MIGR = 0.2`, of each subpopulation is selected for migration. Nearest neighbour subpopulations then exchange these individuals amongst their subpopulations, uniformly reinserting the immigrant individuals (see the *Support for Multiple Populations* Section). The return matrix `Chrom` and vector `ObjV` reflect the changes of individuals in the subpopulations as a result of migration.

The GA iterates around the generational loop until `gen = MAXGEN` and then terminates. Fig. 12 shows a typical solution of the harvest problem obtained by `mpga`.

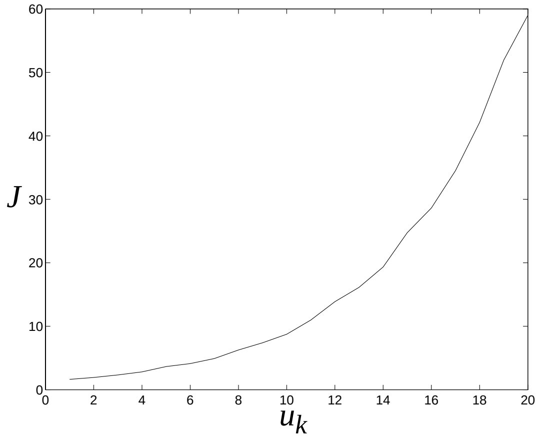

***Figure 12: Optimal Solution Obtained by* `mpga`**

Again, like the previous example, the results of the GA search are contained in the matrix `ObjV`. The objective value and index of the best individual are found using the function `min`, for example:

```
[Y, I] = min(ObjV)
Y =
    -73.2370
I =
    50
```

Remembering that the sign of the objective function has been changed to form a minimisation problem, these results correspond to an objective function value of 73.2370. The exact solution is given as 73.2376. The GA optimal solution is therefore accurate within a 10^{-5} error bound on the exact optimal solution. The chromosome values are displayed in Fig. 12 using:

```
plot(Chrom(I,:))
```

Demonstration Scripts

A number of test functions have been implemented for use with the GA script files supplied with the Toolbox. These test functions are supplied in a separate directory, `test_fns`, from the main demonstrations and Toolbox routines and are accompanied by a postscript file, `test_fns.ps`, giving full details of the problems implemented. The Table below summarises the test functions supplied with the Toolbox.

No.	m-file name	Description
1	`objfun1`	De Jong's function 1
2	`objfun1a`	axis parallel hyper-ellipsoid
3	`objfun1b`	rotated hyper-ellipsoid
4	`objfun2`	Rosenbrock's valley (banana function)
5	`objfun6`	Rastrigin's function
6	`objfun7`	Schwefel's function
7	`objfun8`	Griewangk's function
8	`objfun9`	sum of different powers
9	`objdopi`	double integrator
10	`objharv`	harvest problem
11	`objlinq`	discrete linear-quadratic problem
12	`objlinq2`	continuous linear-quadratic problem
13	`objpush`	push-cart problem

References

[1] D. E. Goldberg, *Genetic Algorithms in Search, Optimization and Machine Learning*, Addison Wesley Publishing Company, January 1989.

[2] C. L. Karr, “Design of an Adaptive Fuzzy Logic Controller Using a Genetic Algorithm”, *Proc. ICGA 4*, pp. 450-457, 1991.

[3] R. B. Holstien, *Artificial Genetic Adaptation in Computer Control Systems*, PhD Thesis, Department of Computer and Communication Sciences, University of Michigan, Ann Arbor, 1971.

[4] R. A. Caruana and J. D. Schaffer, “Representation and Hidden Bias: Gray vs. Binary Coding”, *Proc. 6th Int. Conf. Machine Learning*, pp153-161, 1988.

[5] W. E. Schmitendorgf, O. Shaw, R. Benson and S. Forrest, “Using Genetic Algorithms for Controller Design: Simultaneous Stabilization and Eigenvalue Placement in a Region”, *Technical Report No. CS92-9*, Dept. Computer Science, College of Engineering, University of New Mexico, 1992.

[6] M. F. Bramlette, “Initialization, Mutation and Selection Methods in Genetic Algorithms for Function Optimization”, *Proc ICGA 4*, pp. 100-107, 1991.

[7] C. B. Lucasius and G. Kateman, “Towards Solving Subset Selection Problems with the Aid of the Genetic Algorithm”, In *Parallel Problem Solving from Nature 2*, R. Männer and B. Manderick, (Eds.), pp. 239-247, Amsterdam: North-Holland, 1992.

[8] A. H. Wright, “Genetic Algorithms for Real Parameter Optimization”, In *Foundations of Genetic Algorithms*, J. E. Rawlins (Ed.), Morgan Kaufmann, pp. 205-218, 1991.

[9] Z. Michalewicz, *Genetic Algorithms + Data Structures = Evolution Programs*, Springer Verlag, 1992.

[10] T. Bäck, F. Hoffmeister and H.-P. Schwefel, “A Survey of Evolution Strategies”, *Proc. ICGA 4*, pp. 2-10, 1991.

[11] J. J. Grefenstette, “Incorporating Problem Specific Knowledge into Genetic Algorithms”, In *Genetic Algorithms and Simulated Annealing*, pp. 42-60, L. Davis (Ed.), Morgan Kaufmann, 1987.

[12] D. Whitley, K. Mathias and P. Fitzhorn, “Delta Coding: An Iterative Search Strategy for Genetic Algorithms”, *Proc. ICGA 4*, pp. 77-84, 1991.

[13] K. A. De Jong, *Analysis of the Behaviour of a Class of Genetic Adaptive Systems*, PhD Thesis, Dept. of Computer and Communication Sciences, University of Michigan, Ann Arbor, 1975.

[14] J. E. Baker, “Adaptive Selection Methods for Genetic Algorithms”, *Proc. ICGA 1*, pp. 101-111, 1985.

[15] J. E. Baker, “Reducing bias and inefficiency in the selection algorithm”, *Proc. ICGA 2*, pp. 14-21, 1987.

[16] L. Booker, “Improving search in genetic algorithms,” In *Genetic Algorithms and Simulated Annealing*, L. Davis (Ed.), pp. 61-73, Morgan Kaufmann Publishers, 1987.

[17] W. M. Spears and K. A. De Jong, “An Analysis of Multi-Point Crossover”, In *Foundations of Genetic Algorithms*, J. E. Rawlins (Ed.), pp. 301-315, 1991.

[18] G. Syswerda, “Uniform crossover in genetic algorithms”, Proc. ICGA 3, pp. 2-9, 1989.

[19] W. M. Spears and K. A. De Jong, “On the Virtues of Parameterised Uniform Crossover”, *Proc. ICGA 4*, pp.230-236, 1991.

[20] R. A. Caruana, L. A. Eshelman, J. D. Schaffer, “Representation and hidden bias II: Eliminating defining length bias in genetic search via shuffle crossover”, In *Eleventh International Joint Conference on Artificial Intelligence*, N. S. Sridharan (Ed.), Vol. 1, pp. 750-755, Morgan Kaufmann Publishers, 1989.

[21] H. Mühlenbein and D. Schlierkamp-Voosen, “Predictive Models for the Breeder Genetic Algorithm”, *Evolutionary Computation*, Vol. 1, No. 1, pp. 25-49, 1993.

[22] H. Furuya and R. T. Haftka, “Genetic Algorithms for Placing Actuators on Space Structures”, *Proc. ICGA 5*, pp. 536-542, 1993.

[23] C. Z. Janikow and Z. Michalewicz, “An Experimental Comparison of Binary and Floating Point Representations in Genetic Algorithms”, *Proc. ICGA 4*, pp. 31-36, 1991.

[24] D. M. Tate and A. E. Smith, “Expected Allele Convergence and the Role of Mutation in Genetic Algorithms”, *Proc. ICGA* 5, pp.31-37, 1993.

[25] L. Davis, “Adapting Operator Probabilities in Genetic Algorithms”, *Proc. ICGA 3*, pp. 61-69, 1989.

[26] T. C. Fogarty, “Varying the Probability of Mutation in the Genetic Algorithm”, *Proc. ICGA 3*, pp. 104-109, 1989.

[27] K. A. De Jong and J. Sarma, “Generation Gaps Revisited”, In *Foundations of Genetic Algorithms 2*, L. D. Whitley (Ed.), Morgan Kaufmann Publishers, 1993.

[28] D. Whitley, “The GENITOR algorithm and selection pressure: why rank-based allocations of reproductive trials is best”, *Proc. ICGA 3*, pp. 116-121, 1989.

[29] R. Huang and T. C. Fogarty, “Adaptive Classification and Control-Rule Optimization Via a Learning Algorithm for Controlling a Dynamic System”, *Proc. 30th Conf. Decision and Control*, Brighton, England, pp. 867-868, 1991.

[30] T. C. Fogarty, "An Incremental Genetic Algorithm for Real-Time Learning", *Proc. 6th Int. Workshop on Machine Learning*, pp. 416-419, 1989.

[31] D. E. Goldberg and J. Richardson, "Genetic Algorithms with Sharing for Multimodal Function Optimization", *Proc. ICGA 2*, pp.41-49, 1987.

[32] H. Mühlenbein, M. Schomisch and J. Born, "The Parallel Genetic Algorithm as a Function Optimizer", *Parallel Computing*, No. 17, pp. 619-632, 1991.

[33] T. Starkweather, D. Whitley and K. Mathias, "Optimization Using Distributed Genetic Algorithms", *Proc. Parallel Problem Solving From Nature 1*, Lecture Notes in Computer Science No. 496, pp. 176-185, Springer-Verlag, 1990.

[34] M. Georges-Schleuter, "Comparison of Local Mating Strategies in Massively Parallel Genetic Algorithms", In *Parallel Problem Solving from Nature 2*, R. Männer and B. Manderick, (Eds.), pp. 553-562, Amsterdam: North-Holland, 1992.
