# Supplementary material for: Design of Closed-Loop Control Schemes Based on the GA-PID and GA-RBF-PID Algorithms for Brain Dynamic Modulation
Source: Entropy (Basel). 2023 Nov 15;25(11):1544. doi: 10.3390/e25111544 (PMC10670460; doi:10.3390/e25111544)
Supplement: Supplementary file 1 [file entropy-25-01544-s001.zip › GA_RBF_PID/gatbx/DOC/GATBXA2.PDF]

2 Reference

`bs2rv` 2-5

`crtbase` 2-7

`crtbp` 2-8

`crtrp` 2-10

`migrate` 2-11

`mut` 2-14

`mutate` 2-16

`mutbga` 2-17

`ranking` 2-20

`recdis` 2-24

`recint` 2-26

`reclin` 2-28

`recmut` 2-30

`recombin` 2-33

`reins` 2-34

`rep` 2-37

`rws` 2-38

`scaling` 2-40

`select` 2-41

`sus` 2-44

`xovdp` 2-46

`xovdprs` 2-47

`xovmp` 2-48

`xovsh` 2-49

`xovshrs` 2-50

`xovsp` 2-51

`xovsprs` 2-52

2 Reference

This Section contains detailed descriptions of all of the functions in the Genetic Algorithm Toolbox. It begins with a list of functions grouped by subject area and continues with *Reference* entries in alphabetical order. Information about individual functions is also available through the on-line `Help` facility.

CREATING POPULATIONS	
`crtbase`	create a base vector
`crtbp`	create arbitrary discrete random populations
`crtrp`	create real-valued initial population

FITNESS ASSIGNMENT	
`ranking`	generalised rank-based fitness assignment
`scaling`	proportional fitness scaling

SELECTION FUNCTIONS	
`reins`	uniform random and fitness-based reinsertion
`rws`	roulette wheel selection
`select`	high-level selection routine
`sus`	stochastic universal sampling

MUTATION OPERATORS	
`mut`	discrete mutation
`mutate`	high-level mutation function
`mutbga`	real-value mutation

CROSSOVER OPERATORS	
`recdis`	discrete recombination
`recint`	intermediate recombination
`reclin`	line recombination
`recmut`	line recombination with mutation features
`recombin`	high-level recombination operator
`xovdp`	double-point crossover
`xovdprs`	double-point reduced surrogate crossover
`xovmp`	general multi-point crossover
`xovsh`	shuffle crossover
`xovshrs`	shuffle reduced surrogate crossover
`xovsp`	single-point crossover
`xovsprs`	single-point reduced surrogate crossover

SUBPOPULATION SUPPORT	
`migrate`	exchange individuals between subpopulations

UTILITY FUNCTIONS	
`bs2rv`	binary string to real-value conversion
`rep`	matrix replication

bs2rv

Purpose

binary string to real value conversion

Synopsis

`Phen = bs2rv(Chrom, FieldD)`

Description

`Phen = bs2rv(Chrom, FieldD)` decodes the binary representation of the population, `Chrom`, into vectors of reals. The chromosomes are seen as concatenated binary strings of given length, and decoded into real numbers over a specified interval using either standard binary or Gray coding according to the decoding matrix, `FieldD`. The resulting matrix, `Phen`, contains the corresponding population phenotypes.

The use of Gray coding for binary chromosome representation is recommended as the regular Hamming distance between quantization intervals reportedly makes the genetic search less deceptive (see, for example, [1, 2]). An option to set the scaling between quantization points can be used to select either linear or logarithmic decoding to real values from binary strings. Logarithmic scaling is useful when the range of decision variable is unknown at the outset as a wider range of parametric values can be searched with fewer bits [3], thus reducing the memory and computational requirements of the GA.

The matrix `FieldD` has the following structure:

$$\begin{bmatrix} \texttt{len} \\ \texttt{lb} \\ \texttt{ub} \\ \texttt{code} \\ \texttt{scale} \\ \texttt{lbin} \\ \texttt{ubin} \end{bmatrix},$$

where the rows of the matrix are composed as follows:

`len`, a row vector containing the length of each substring in `Chrom`. Note that `sum(len)` should equal `length(Chrom)`.

`lb` and `ub` are row vectors containing the lower and upper bounds respectively for each variable used.

`code` is a binary row vector indicating how each substring is decoded. Select `code(i) = 0` for standard binary and `code(i) = 1` for Gray coding.

`scale` is a binary row vector indicating whether to use arithmetic and/or logarithmic scaling for each substring. Select `scale(i) = 0` for arithmetic scaling and `scale(i) = 1` for logarithmic scaling.

`lbin` and `ubin` are binary row vectors indicating whether or not to include each bound in the representation range. Select `{l|u}bin(i) = 0` to exclude `{l|u}b(i)` from the representation range and `{l|u}bin(i) = 1` to include `{l|u}b(i)` in the representation range.

Example

Consider the following binary population, created using the `crtbp` function, representing a set of single decision variables in the range [-1, 10]. The code extract shows how the function `bs2rv` may be used to convert the Gray code binary representation to real-valued phenotypes using arithmetic scaling.

```
Chrom = crtbp(4,8) % create random chromosomes

Chrom =
   0  0  0  0  0  1  1  1
   1  0  0  0  1  0  0  1
   0  0  1  0  1  0  0  0
   1  1  0  1  1  0  1  1

FieldD = [8; -1; 10; 1; 0; 1; 1]; % representation

Phen = bs2rv(Chrom,FieldD) % convert binary to real

Phen =
   -0.7843
    9.3961
    1.0706
    5.2980
```

Algorithm

`bs2rv` is implemented as an m-file in the GA Toolbox. If logarithmic scaling is used then the range must not include zero.

Reference

[1] R. B. Holstien, *Artificial Genetic Adaptation in Computer Control Systems*, Ph.D. Thesis, Department of Computer and Communication Sciences, University of Michigan, Ann Arbor, 1971.

[2] R. A. Caruana and J. D. Schaffer, “Representation and Hidden Bias: Gray vs. Binary Coding”, *Proc. 6th Int. Conf. Machine Learning*, pp153-161, 1988.

[3] W. E. Schmitendorgf, O. Shaw, R. Benson and S. Forrest, “Using Genetic Algorithms for Controller Design: Simultaneous Stabilization and Eigenvalue Placement in a Region”, *Technical Report No. CS92-9*, Dept. Computer Science, College of Engineering, University of New Mexico, 1992.

crtbase

Purpose

Create a base vector.

Synopsis

`BaseVec = crtbase(Lind, Base)`

Description

`crtbase` produces a vector whose elements correspond to the base of the loci of a chromosome structure. This function can be used in conjunction with `crtbp` when creating populations using representations in different bases.

`BaseVec = crtbase(Lind, Base)` creates a vector of length `Lind` whose individual elements are of base `Base`. If `Lind` is a vector, then `length(BaseVec) = sum(Lind)`. If `Base` is also a vector of `length(Lind)`, then `BaseVec` is composed of groups of bases of length determined by the elements of `Lind` and base `Base`. This last option is useful when describing populations with structure.

Example

To create a basevector for a population containing four elements in base 8 and five elements in base four:

```
BaseV = crtbase([4 5], [8 4])
```

```
BaseV =
   8  8  8  8  4  4  4  4  4
```

See Also

`crtbp`, `bs2rv`

crtbp

Purpose

Create an initial population.

Synopsis

```
[Chrom, Lind, BaseV] = crtbp(Nind, Lind)
[Chrom, Lind, BaseV] = crtbp(Nind, Lind, Base)
[Chrom, Lind, BaseV] = crtbp(Nind, BaseV)
```

Description

The first step in a genetic algorithm is to create an initial population consisting of random chromosomes. `crtbp` produces a matrix, `Chrom`, containing random values in its elements.

`Chrom = crtbp(Nind, Lind)` creates a random binary matrix of size `Nind` × `Lind`, where `Nind` specifies the number of individuals in the population and `Lind` the length of the individuals. Additionally, `Chrom = crtbp([Nind, Lind])` can be used to specify the dimensions of the chromosome matrix.

`Chrom = crtbp(Nind, Lind, Base)` produces a chromosome matrix of base `Base`. If `Base` is a vector, then the value of the elements of `Base` specify the base of the loci of the chromosomes. In this case, the second right hand side argument may be omitted, `Chrom = crtbp(Nind, BaseV)`.

`[Chrom, Lind, BaseV] = crtbp(Nind, BaseV)` also returns the length of the chromosome structure, `Lind`, and the base of the chromosome loci in the vector `BaseV`.

Example

To create a random population of 6 individuals of length 8 where the first four loci are base eight and the last five loci are base four:

```
BaseV = crtbase([4 5], [8 4])
Chrom = crtbp(6, BaseV)
```

or

```
Chrom = crtbp([6,8],[8 8 8 8 4 4 4 4 4])
```

```
Chrom =
   4  3  1  1  2  0  2  0  3
   1  4  7  5  2  1  1  1  0
   1  3  0  1  0  0  0  0  2
   1  5  5  7  2  0  2  3  1
   4  5  7  7  0  1  3  0  3
   4  2  4  0  3  3  1  1  0
```

Algorithm

`crtbp` is an m-file in the GA Toolbox that uses the MATLAB function `rand`.

See Also

`crtbase`, `crtrp`

crtrp

Purpose

Create a real-valued initial population

Synopsis

```
Chrom = crtrp(Nind, FieldDR)
```

Description

The first step in a genetic algorithm is to create an initial population consisting of random individuals. `crtrp` produces a matrix, `Chrom`, containing uniformly distributed random values in its elements.

`Chrom = crtrp(Nind, FiedDR)` creates a random real-valued matrix of size `Nind` × `Nvar`, where `Nind` specifies the number of individuals in the population and `Nvar` the number of variables of each individual. `Nvar` is derived from `FieldDR` with `Nvar = size(FieldDR,2)`.

`FieldDR` (FieldDescriptionRealvalue) is a matrix of size 2 × `Nvar` and contains the boundaries of each variable of an individual. The first row contains the lower bounds, the second row the upper bounds.

`FieldDR` is used in other functions (mutation).

Example

To create a random population of 6 individuals with 4 variables each:

Define boundaries on the variables,

```
FieldDR = [
   -100 -50 -30 -20;  % lower bound
    100  50  30  20]; % upper bound
```

Create initial population

```
Chrom = crtrp(6, FieldDR)
```

```
Chrom =
     40.23 -17.17  28.95  15.38
     82.06  13.26  13.35  -9.09
     52.43  25.64  15.20  -2.54
    -47.50  49.10   9.09  10.65
    -90.50 -13.46 -25.63  -0.89
     47.21 -25.29   7.89 -10.48
```

See Also

`mutbga`, `recdis`, `recint`, `reclin`

migrate

Purpose

Migrate individuals between subpopulations

Synopsis

```
Chrom = migrate(Chrom, SUBPOP)

Chrom = migrate(Chrom, SUBPOP, MigOpt)

Chrom = migrate(Chrom, SUBPOP, MigOpt, ObjV)

[Chrom, ObjV] = migrate(Chrom, SUBPOP, MigOpt, ObjV)
```

Description

`migrate` performs migration of individuals between subpopulations in the current population, `Chrom`, and returns the population after migration, `Chrom`. Each row of `Chrom` corresponds to one individual. The number of subpopulations is indicated by `SUBPOP`. The subpopulations in `Chrom` are ordered according to the following scheme:

$$\mathtt{Chrom} = \begin{bmatrix} \mathtt{Ind}_1\,\mathtt{SubPop}_1 \\ \mathtt{Ind}_2\,\mathtt{SubPop}_1 \\ \ldots \\ \mathtt{Ind}_N\,\mathtt{SubPop}_1 \\ \mathtt{Ind}_1\,\mathtt{SubPop}_2 \\ \mathtt{Ind}_2\,\mathtt{SubPop}_2 \\ \ldots \\ \mathtt{Ind}_N\,\mathtt{SubPop}_2 \\ \ldots \\ \mathtt{Ind}_1\,\mathtt{SubPop}_{\mathtt{SUBPOP}} \\ \mathtt{Ind}_2\,\mathtt{SubPop}_{\mathtt{SUBPOP}} \\ \ldots \\ \mathtt{Ind}_N\,\mathtt{SubPop}_{\mathtt{SUBPOP}} \end{bmatrix}$$

All subpopulations must have the same number of individuals.

`MigOpt` is an optional vector with a maximum of 3 parameters:

MigOpt(1):

scalar containing the rate of migration of individuals between subpopulations in the range [0, 1]
If omitted or NaN, MigOpt(1) = 0.2 (20%) is assumed.
If the migration rate is greater than 0 at least one individual per subpopulation will migrate.

MigOpt(2):

scalar specifying the migration selection method
0 - uniform migration
1 - fitness-based migration
If omitted or NaN, MigOpt(2) = 0 is assumed.

MigOpt(3):

scalar indicating the structure of the subpopulations for migration
0 - complete net structure
1 - neighbourhood structure
2 - ring structure
If omitted or NaN, MigOpt(3) = 0 is assumed

If MigOpt is omitted or NaN, then the default values are assumed.

ObjV is an optional column vector with as many rows as Chrom and contains the corresponding objective values for all individuals in Chrom. For fitness-based selection of individuals (MigOpt(2) = 1) ObjV is necessary. If ObjV is an input and output parameter, the objective values are copied according to the migration of the individuals. This saves the recomputation of the objective values for the whole population.

Example

Chrom = migrate(Chrom, SUBPOP) chooses 20% of the individuals of one subpopulation and replaces these individuals with uniformly chosen individuals from all other subpopulations. This process is done for each subpopulation. (MigOpt = [0.2, 0, 0])

Chrom = migrate(Chrom, SUBPOP, [NaN 1 NaN], ObjV]) chooses 20% of the individuals of one subpopulation and replaces these individuals with a selection of the fittest individuals (smaller ObjV) from all other subpopulations. (net structure) This process is repeated for each subpopulation.

[Chrom,ObjV] = migrate(Chrom,SUBPOP,[0.3 1 2],ObjV]) chooses 30% of the individuals of one subpopulation and replaces these individuals with the fittest individuals (smaller ObjV) from an adjacent subpopulation in a unidirectional ring structure. This process is repeated for each subpopulation. The first subpopulation receives its new individuals from the last subpopulation (SUBPOP). ObjV is returned according to the migration of individuals.

The migration scheme employed:
subpop1-->subpop2-->subpop3-->...-->subpopSUBPOP--> subpop1

`[Chrom,ObjV] = migrate(Chrom,SUBPOP,[NaN NaN 1],ObjV])` chooses 20% of the individuals of one subpopulation and replaces these individuals with uniformly chosen individuals from both adjacent subpopulations in an one dimensional neighborhood structure. This process is repeated for each subpopulation. The first subpopulation receives its new individuals from the last (`SUBPOP`) and second subpopulation the last subpopulation from the first and `SUBPOP-1` subpopulation. `ObjV` is returned according to the migration of individuals.
The migration scheme employed:
subpopSUBPOP-->subpop1<-->subpop2<-->...<-->subpopSUBPOP<--subpop1

See Also

`select`, `recombin`, `mutate`, `reins`

Reference

[1] H. Mühlenbein, M. Schomisch and J. Born, “The Parallel Genetic Algorithm as a Function Optimizer”, *Parallel Computing*, No. 17, pp.619-632, 1991.

[2] T. Starkweather, D. Whitley and K. Mathias, “Optimization using Distributed Genetic Algorithms”, In *Parallel Problems Solving from Nature*, Lecture Notes in Computer Science, Vol. 496, pp. 176-185, Springer, 1991.

[3] R. Tanese, “Distributed Genetic Algorithms”, *Proc. ICGA 3*, pp. 434-439, Morgan Kaufmann Publishers, 1989.

[4] H.-M. Voigt, J. Born and I. Santibanez-Koref, “Modelling and Simulation of Distributed Evolutionary Search Processes for Function Optimization”, Parallel Problems Solving from Nature, Lecture Notes in Computer Science, Vol. 496, pp. 373-380, Springer Verlag, 1991.

mut

Purpose

Discrete mutation operator

Synopsis

`NewChrom = mut(OldChrom, Pm, BaseV)`

Description

`mut` takes the representation of the current population and mutates each element with a given probability. To allow for varying bases in the chromosome and structured populations, `mut` allows an additional argument `BaseV` that specifies the base of the individual elements of a chromosome.

`NewChrom = mut(OldChrom, Pm)` takes the current population, `OldChrom`, with each row corresponding to an individuals, and mutates each element with probability `Pm`. If the mutation probability, `Pm`, is omitted, `Pm=0.7/Lind` is assumed, where `Lind` is the length of the chromosome structure. This value is selected as it implies that the probability of any one element of a chromosome being mutated is approximately 0.5 (see [1]). Without a third input argument, `mut` assumes that the population is binary coded.

`NewChrom = (OldChrom, Pm, BaseV)` uses a third argument to specify the base of the mutation of the individual elements of the chromosomes. In this case, `length(BaseV) = Lind`, where `Lind` is the length of the chromosome structure.

`mut` is a low-level mutation function normally called by `mutate`.

Example

Consider a binary population `OldChrom` with 4 individuals each of length 8:

```
OldChrom =[
   0 0 0 0 0 1 1 1;
   1 0 0 0 1 0 0 1;
   0 0 1 0 1 0 0 0;
   1 1 0 1 1 0 1 1]
```

Mutate `OldChrom` with default probability:

```
NewChrom = mut(OldChrom)
```

Thus, `NewChrom` can become:

```
NewChrom =
   0  0  1  0  0  1  1  1
   1  1  0  0  0  0  0  1
   0  0  0  0  1  0  0  0
   1  1  0  1  1  0  1  1
```

The complement of a binary string is obtained by applying mutation with probability 1.

```
mut([1 0 1 0 1 1 1 0], 1)
```

```
ans =
   0  1  0  1  0  0  0  1
```

See Also

`mutate`, `mutbga`

Reference

[1] Jürgen Hesser and Reinhard Männer, "Towards an Optimal Mutation Rate Probability for Genetic Algorithms", In *Parallel Problem Solving from Nature*, Lecture Notes in Computer Science, Vol. 496, pp23-32, 1990.

mutate

Purpose

Mutation of individuals (high-level function).

Synopsis

```
NewChrom = mutate(MUT_F, OldChrom, FieldDR)
NewChrom = mutate(MUT_F, OldChrom, FieldDR, MutOpt)
NewChrom = mutate(MUT_F, OldChrom, FieldDR, MutOpt,
   SUBPOP)
```

Description

`mutate` performs mutation of individuals from a population, `OldChrom`, and returns the mutated individuals in a new population, `NewChrom`. Each row of `OldChrom` and `NewChrom` corresponds to one individual.

`MUT_F` is a string that contains the name of the low-level mutation function, e.g. `mutbga` or `mut`.

`FieldDR` is a matrix of size $2 \times \mathtt{Nvar}$ and contains the bounds of each variable of an individual (real-valued variables) or a matrix of size $1 \times \mathtt{Nvar}$ and contains the base of each variable (discrete-valued variables). If `FieldDR` is omitted, empty or `NaN`, a binary representation of the variables is assumed.

`MutOpt` is an optional parameter containing the mutation rate, the probability of mutating a variable of an individual. If `MutOpt` is omitted, a default mutation rate is assumed. For real-value mutation `MutOpt` can contain a second parameter specifying a scalar for shrinking the mutation range (see `mutbga`).

`SUBPOP` is an optional parameter and determines the number of subpopulations in `OldChrom`. If `SUBPOP` is omitted or `NaN`, `SUBPOP = 1` is assumed. All subpopulations in `OldChrom` must have the same size.

Example

For examples, see `mutbga` (real-value mutation) and `mut` (discrete-value mutation).

Algorithm

`mutate` checks the consistency of the input parameters and calls the low-level mutation function. If `mutate` is called with more than one subpopulation then the low-level mutation function is called separately for each subpopulation.

See Also

`mutbga`, `mut`, `recombin`, `select`

mutbga

Purpose

Mutation of real-valued population (mutation operator of the breeder genetic algorithm).

Synopsis

```
NewChrom = mutbga(OldChrom, FieldDR)
NewChrom = mutbga(OldChrom, FieldDR, MutOpt)
```

Description

`mutbga` takes the real-valued population, `OldChrom`, mutates each variable with given probability and returns the population after mutation, `NewChrom`.

`NewChrom = mutbga(OldChrom, FieldDR, MutOpt)` takes the current population, stored in the matrix `OldChrom` and mutates each variable with probability `MutOpt(1)` by addition of small random values (size of the mutation step). The mutation step can be shrunk with `MutOpt(2)`.

`FieldDR` is a matrix containing the boundaries of each variable of an individual (see `crtrp`).

`MutOpt` is an optional vector with a maximum of two parameters:

`MutOpt(1)`:

scalar containing the mutation rate in the range `[0, 1]`.
If omitted or `NaN`, `MutOpt(1) = 1/Nvar` is assumed, where `Nvar` is the number of variables per individual defined by `size(FieldDR,2)`. This value is selected as it implies that the number of variables per individual mutated is approximately 1.

`MutOpt(2)`:

scalar containing a value in the range [0, 1] for shrinking the mutation range.
If omitted or `NaN`, `MutOpt(2) = 1` is assumed (no shrinking).

`mutbga` is a low-level mutation function normally called by `mutate`.

Example

Consider the following population with three real-valued individuals:

```
OldChrom = [
   40.2381 -17.1766  28.9530  15.3883;
   82.0642  13.2639  13.3596  -9.0916;
   52.4396  25.6410  15.2014  -2.5435]
```

The bounds are defined as:

```
FieldDR = [
   -100 -50 -30 -20;
    100  50  30  20]
```

To mutate `OldChrom` with mutation probability `1/4` and no shrinking of the mutation range:

```
NewChrom = mutbga(OldChrom, FieldDR, [1/4 1.0])
```

`mutbga` produces an internal mask table, `MutMx`, determining which variable to mutate and the sign for adding `delta` (see Algorithm), e.g.

```
MutMx = [
   0  0  0  1;
   0  0 -1  0;
   0  0 -1 -1]
```

An second internal table, `delta`, specifies the normalized mutation step size, e.g.

```
delta = [
   0.2500 0.2500 0.2500 0.2500;
   0.0001 0.0001 0.0001 0.0001;
   0.2505 0.2505 0.2505 0.2505]
```

Thus, after mutation `NewChrom` becomes:

```
NewChrom =
   40.2381 -17.1766 28.9530 20.0000
   82.0642  13.2638 13.3559 -9.0916
   52.4396  25.6410 -7.6858 -7.5539
```

`NewChrom - OldChrom` shows the mutation steps

```
NewChrom - OldChrom =
         0       0       0  4.6117
         0       0 -0.0037       0
         0       0 -7.5156 -5.0104
```

Algorithm

The mutation of a variable is computed as follows:

mutated variable = variable + `MutMx` × `range` × `MutOpt(2)` × `delta`

`MutMx` = ±1 with probability `MutOpt(1)`, (+ or - with equal probability)
else 0

`range` = 0.5 × domain of variable (search interval defined by `FieldDR`).

$$\texttt{delta} = \sum_{i=0}^{m-1} \alpha_i 2^{-i},\ \alpha_i = 1 \text{ with probability 1/m, else 0, m = 20.}$$

With m = 20, the mutation operator is able to locate the optimum up to a precision of `range` × `MutOpt(2)` × 2^{-19}.

The mutation operator `mutbga` is able to generate most points in the hypercube defined by the variables of the individual and the range of the mutation. However, it tests more often near the variable, that is, the probability of small step sizes is greater than that of larger step sizes.

See Also

`mutate`, `recdis`, `recint`, `reclin`

Reference

[1] H. Mühlenbein and D. Schlierkamp-Voosen, “Predictive Models for the Breeder Genetic Algorithm: I. Continuous Parameter Optimization”, *Evolutionary Computation*, Vol. 1, No. 1, pp.25-49, 1993.

ranking

Purpose

Rank-based fitness assignment

Synopsis

```
FitnV = ranking(ObjV)
FitnV = ranking(ObjV, RFun)
FitnV = ranking(ObjV, RFun, SUBPOP)
```

Description

`ranking` ranks individuals according to their objective values, `ObjV`, and returns a column vector containing the corresponding individual fitness values, `FitnV`. This function ranks individuals for **minimisation**.

`RFun` is an optional vector with 1, 2 or `length(ObjV)` parameters:

If `RFun` is a scalar in `[1, 2]`, linear ranking is assumed and the scalar indicates the selective pressure.

If `RFun` is a vector with 2 parameters:

`RFun(1)`:
    scalar indicating the selective pressure
    for linear ranking `RFun(1)` must be in `[1, 2]`
    for non-linear ranking `RFun(1)` must be in `[1, length(ObjV)-2]`
    If `NaN`, `RFun(1) = 2` is assumed.

`RFun(2)`:
    ranking method
    0 - linear ranking
    1 - non-linear ranking

If `RFun` is a vector of `length(ObjV)`, it should contain the fitness values to be assigned to each rank.

If `RFun` is omitted or `NaN`, linear ranking and a selective pressure of 2 are assumed.

`SUBPOP` is an optional parameter and indicates the number of subpopulations in `ObjV`. If `SUBPOP` is omitted or `NaN`, `SUBPOP = 1` is assumed. All subpopulations in `ObjV` must have the same size.

If `ranking` is called with more than one subpopulation then the ranking is performed separately for each subpopulation.

Example

Consider a population with 10 individuals. The current objective values are:

```
ObjV = [1; 2; 3; 4; 5; 10; 9; 8; 7; 6]
```

Evaluate the fitness with linear ranking and selective pressure 2:

```
FitnV = ranking(ObjV)

FitnV =
    2.00
    1.77
    1.55
    1.33
    1.11
    0
    0.22
    0.44
    0.66
    0.88
```

Evaluate the fitness with non-linear ranking and selective pressure 2:

```
FitnV = ranking(ObjV, [2 1])

FitnV =
    2.00
    1.66
    1.38
    1.15
    0.95
    0.38
    0.45
    0.55
    0.66
    0.79
```

Evaluate the fitness with the values in RFun:

```
RFun = [3; 5; 7; 10; 14; 18; 25; 30; 40; 50]

FitnV = ranking(ObjV, RFun)
```

```
FitnV =
   50
   40
   30
   25
   18
    3
    5
    7
   10
   14
```

Evaluate the fitness with non-linear ranking and selective pressure 2 for 2 subpopulations in `ObjV`:

```
FitnV = ranking(ObjV, [2 1], 2)
```

```
FitnV =
   2.00
   1.28
   0.83
   0.53
   0.34
   0.34
   0.53
   0.83
   1.28
   2.00
```

Algorithm

The algorithms for both linear and non-linear ranking first sorts the objective function values into descending order. The least fit individual is placed in position 1 in the sorted list of objective values and the most fit individual position `Nind` where `Nind` is the number of individuals in the population. A fitness value is then assigned to each individual depending on its position, `Pos`, in the sorted population.

For linear ranking individuals are assigned fitness values according to:

$\mathtt{FitnV(Pos)} = 2 - \mathtt{SP} + 2 \times (\mathtt{SP} - 1) \times (\mathtt{Pos} - 1) / (\mathtt{Nind} - 1)$, and

for non-linear ranking according to:

$$\mathtt{FitnV(Pos)} = \frac{\mathtt{Nind} \times X^{Pos - 1}}{\sum_{\mathtt{i} = 1}^{\mathtt{Nind}} X(\mathtt{i})},$$

where *X* is computed as the root of the polynomial:

$$0 = (SP-1) \times X^{Nind-1} + SP \times X^{Nind-2} + \ldots + SP \times X + SP.$$

The vector `FitnV` is then unsorted to reflect the order of the original input vector, `ObjV`.

See Also

`select`, `rws`, `sus`

Reference

[1] D. Whitley, “The GENITOR Algorithm and Selection Pressure: Why Rank-Based Allocation of Reproductive Trials is Best”, *Proc. ICGA 3*, pp. 116-121, Morgan Kaufmann Publishers, 1989.

recdis

Purpose

Discrete recombination

Synopsis

```
NewChrom = recdis(OldChrom)
```

Description

`recdis` performs discrete recombination between pairs of individuals in the current population, `OldChrom`, and returns a new population after mating, `NewChrom`. Each row of `OldChrom` corresponds to one individual.

The pairs are mated in order, odd row with the next even row. If the number of rows in the matrix `OldChrom` is odd then the last row is not mated and added at the end of `NewChrom`. The population should therefore be organised into contiguous pairs that require mating. This can be achieved by using the function `ranking` to assign a fitness level to each individual and a selection function (e.g. `select`) to select individuals with a probability related to their fitness in the current population.

`recdis` is a low-level recombination function normally called by `recombin`.

Example

Consider the following population with five real-value individuals:

```
OldChrom = [
    40.23 -17.17  28.95  15.38; % parent1
    82.06  13.26  13.35  -9.09; % parent2
    52.43  25.64  15.20  -2.54; % parent3
   -47.50  49.10   9.09  10.65; % parent4
   -90.50 -13.46 -25.63  -0.89] % parent5
```

To perform discrete recombination:

```
NewChrom = recdis(OldChrom)
```

`recdis` produces an internal mask table determining which parents contribute which variables to the offspring, e.g.

```
Mask = [
    1 2 1 2; % for producing offspring1
    2 2 1 1; % for producing offspring2
    2 1 2 1; % for producing offspring3
    1 1 2 2] % for producing offspring4
```

Thus, after recombination `NewChrom` would become:

```
NewChrom = [
    40.23  13.26  28.95  -9.09; % Mask(1,:) parent1&2
    82.06  13.26  28.95  15.38; % Mask(2,:) parent1&2
   -47.50  25.64   9.09  -2.54; % Mask(3,:) parent3&4
    52.43  25.64   9.09  10.65] % Mask(4,:) parent3&4
```

As the number of individuals in the parent population, `OldChrom`, was odd, the last individual is appended without recombination to `NewChrom` and the offspring returned to the users workspace, thus

```
NewChrom =
    40.23  13.26  28.95  -9.09
    82.06  13.26  28.95  15.38
   -47.50  25.64   9.09  -2.54
    52.43  25.64   9.09  10.65
   -90.50 -13.46 -25.63  -0.89
```

Algorithm

Discrete recombination exchanges variable values between the individuals. For each variable the parent who contributes its variable value to the offspring is chosen randomly with equal probability.

Discrete recombination can generate the corners of the hypercube defined by the parents.

See Also

`recombin`, `recint`, `reclin`, `ranking`, `sus`, `rws`

Reference

[1] H. Mühlenbein and D. Schlierkamp-Voosen, "Predictive Models for the Breeder Genetic Algorithm: I. Continuous Parameter Optimization", *Evolutionary Computation*, Vol. 1, No. 1, pp.25-49, 1993.

recint

Purpose

Intermediate recombination

Synopsis

`NewChrom = recint(OldChrom)`

Description

`recint` performs intermediate recombination between pairs of individuals in the current population, `OldChrom`, and returns a new population after mating, `NewChrom`. Each row of `OldChrom` corresponds to one individual.

`recint` is a function only applicable to populations of real-value variables (and not binary or integer).

The pairs are mated in order, odd row with the next even row. If the number of rows in the matrix `OldChrom` is odd then the last row is not mated and added at the end of `NewChrom`. The population should therefore be organised into contiguous pairs that require mating. This can be achieved by using the function `ranking` to assign a fitness level to each individual and a selection function (e.g. `select`) to select individuals with a probability related to their fitness in the current population.

`recint` is a low-level recombination function normally called by `recombin`.

Example

Consider the following population with three real-value individuals:

```
OldChrom = [
   40.23 -17.17  28.95  15.38; % parent1
   82.06  13.26  13.35  -9.09; % parent2
   52.43  25.64  15.20  -2.54] % parent3
```

To perform intermediate recombination:

```
NewChrom = recint(OldChrom)
```

New values are produced by adding the scaled difference between the parent values to the first parent (see *Algorithm* subsection). An internal table of scaling factors, `Alpha`, is produced, e.g.

```
Alpha = [
   -0.13 0.50 0.32 0.16; % for offspring1
    1.12 0.54 0.44 1.16] % for offspring2
```

Thus, after recombination `NewChrom` would become:

```
NewChrom = [
   34.40  -1.92  23.86  11.33; % Alpha(1,:) parent1&2
   87.11  -0.59  21.98 -13.04] % Alpha(2,:) parent1&2
```

As the number of individuals in the parent population, `OldChrom`, was odd, the last individual is appended without recombination to `NewChrom` and the offspring returned to the users workspace, thus:

```
NewChrom =
   34.40  -1.92  23.86  11.33
   87.11  -0.59  21.98 -13.04
   52.43  25.64  15.20  -2.54
```

Algorithm

Intermediate recombination combines parent values using the following rule:

$$\texttt{offspring} = \texttt{parent1} + \texttt{Alpha} \times (\texttt{parent2} - \texttt{parent1})$$

where `Alpha` is a scaling factor chosen uniformly at random in the interval `[-0.25, 1.25]`. `recint` produces a new Alpha for each pair of values to be combined.

Intermediate recombination can generate any point within a hypercube slightly larger than that defined by the parents.

Intermediate recombination is similar to line recombination `reclin`. Whereas `recint` uses a new Alpha factor for each pair of values combined together, `reclin` uses one `Alpha` factor for each pair of parents.

See Also

`recombin`, `recdis`, `reclin`, `ranking`, `sus`, `rws`

Reference

[1] H. Mühlenbein and D. Schlierkamp-Voosen, “Predictive Models for the Breeder Genetic Algorithm: I. Continuous Parameter Optimization”, *Evolutionary Computation*, Vol. 1, No. 1, pp.25-49, 1993.

reclin

Purpose

Line recombination

Synopsis

```
NewChrom = reclin(OldChrom)
```

Description

`reclin` performs line recombination between pairs of individuals in the current population, `OldChrom`, and returns a new population after mating, `NewChrom`. Each row of `OldChrom` corresponds to one individual.

`reclin` is a function only applicable to populations of real-value variables (**not** binary or integer).

The pairs are mated in order, odd row with the next even row. If the number of rows in the matrix `OldChrom` is odd then the last row is not mated and added at the end of `NewChrom`. The population should therefore be organised into contiguous pairs that require mating. This can be achieved by using the function `ranking` to assign a fitness level to each individual and a selection function (e.g. `select`) to select individuals with a probability related to their fitness in the current population.

`reclin` is a low-level recombination function normally called by `recombin`.

Example

Consider the following population with three real-value individuals:

```
OldChrom = [
    40.23 -17.17  28.95  15.38; % parent1
    82.06  13.26  13.35  -9.09; % parent2
    52.43  25.64  15.20  -2.54] % parent3
```

To perform line recombination:

```
NewChrom = reclin(OldChrom)
```

New values are produced by adding the scaled difference between the parent values to the first parent (see Algorithm). An internal table of scaling factors, `Alpha`, is produced, e.g.

```
Alpha = [
   0.78; % for producing offspring1
   1.05] % for producing offspring2
```

Thus, after recombination NewChrom would become:

```
NewChrom = [
   72.97  6.64 16.74  -3.77; % Alpha(1) parent1&2
   84.25 14.85 12.54 -10.37] % Alpha(2) parent1&2
```

As the number of individuals in the parent population, `OldChrom`, was odd, the last individual is appended without recombination to `NewChrom` and the offspring returned to the users workspace, thus:

```
NewChrom =
   72.97  6.64 16.74  -3.77
   84.25 14.85 12.54 -10.37
   52.43 25.64 15.20  -2.54
```

Algorithm

Line recombination combines parent values using the following rule:

$$\texttt{offspring = parent1 + Alpha} \times (\texttt{parent2} - \texttt{parent1})$$

where `Alpha` is a scaling factor chosen uniformly at random in the interval `[-0.25, 1.25]`. `reclin` produces a new Alpha for each pair of parents to be combined.

Line recombination can generate any point on a slightly longer line than that defined by the parents.

Line recombination is similar to intermediate recombination `recint`. Whereas `reclin` uses one Alpha factor for each pair of parents combined together, `recint` uses a new `Alpha` factor for each pair of values.

See Also

`recombin`, `recdis`, `recint`, `ranking`, `sus`, `rws`

Reference

[1] H. Mühlenbein and D. Schlierkamp-Voosen, “Predictive Models for the Breeder Genetic Algorithm: I. Continuous Parameter Optimization”, *Evolutionary Computation*, Vol. 1, No. 1, pp.25-49, 1993.

recmut

Purpose

Line recombination with mutation features

Synopsis

```
NewChrom = recmut(OldChrom, FieldDR)
NewChrom = recmut(OldChrom, FieldDR, MutOpt)
```

Description

`recmut` performs line recombination with mutation features between pairs of individuals in the current population, `OldChrom`, and returns a new population after mating, `NewChrom`. Each row of `OldChrom` corresponds to one individual.

`FieldDR` is a matrix containing the boundaries of each variable of an individual (see `crtrp`).

`MutOpt` is an optional vector with a maximum of 2 parameters:

`MutOpt(1)`:
: scalar containing the recombination rate in the range `[0, 1]`.
  If omitted or `NaN`, `MutOpt(1) = 1` is assumed.

`MutOpt(2)`:
: scalar containing a value in the range [0, 1] for shrinking the recombination range.
  If omitted or `NaN`, `MutOpt(2) = 1` is assumed (no shrinking).

`recmut` is a function only applicable to populations of real-value variables (and not binary or integer).

The pairs are mated in order, odd row with the next even row. If the number of rows in the matrix `OldChrom` is odd then the last row is not mated and added at the end of `NewChrom`. The population should therefore be organised into contiguous pairs that require mating. This can be achieved by using the function `ranking` to assign a fitness level to each individual and a selection function (`sus` or `rws`) to select individuals with a probability related to their fitness in the current population.

`recmut` uses features of the mutation operator of the Breeder Genetic Algorithm (see `mutbga`). Therefore, the calling syntax of this recombination function is identical to this of the mutation function `mutbga`.

`recmut` is a low-level recombination function normally called by `mutate`.

Example

Consider the following population with four real-valued individuals:

```
OldChrom = [
    40.2381 -17.1766  28.9530  15.3883;  % parent1
    82.0642  13.2639  13.3596  -9.0916;  % parent2
    52.4396  25.6410  15.2014  -2.5435;  % parent3
   -47.5381  49.1963   9.0954  10.6521]  % parent4
```

The boundaries are defined as:

```
FieldDR = [
   -100 -50 -30 -20;
    100  50  30  20]
```

To perform line recombination with mutation features:

```
NewChrom = recmut(OldChrom, FieldDR)
```

`recmut` produces an internal mask table, `RecMx`, determining which pairs of parents to recombine (here recombine all pairs) and the sign for adding `the` recombination step (see Algorithm), e.g.

```
RecMx = [
    1 -1 -1 -1; % for producing offspring1 & 2
   -1 -1 -1 -1] % for producing offspring3 & 4
```

Two further internal tables, `delta` and `Diff`, specify the normalized recombination step size, e.g.

```
delta = [
   0.1250 0.1250 0.1250 0.1250; % for offspring1 & 2
   0.0005 0.0005 0.0005 0.0005] % for offspring3 & 4
```

```
Diff = [
     1.3937 1.0143 -0.5196 -0.8157; % for offspring1 & 2
   -10.5712 2.4906 -0.6456  1.3952] % for offspring3 & 4
```

Thus, after recombination NewChrom becomes:

```
NewChrom =
    57.6637 -23.5177  30.0000  17.4281
    64.6386  19.6050  11.4106 -11.1314
    52.9719  25.5783  15.2112  -2.5576
   -48.0704  49.2590   9.0856  10.6662
```

Algorithm

The offsprings of a pair of two parents are computed as follows:

offspring1=parent1 + `RecMx` × `range` × `MutOpt(2)` × `delta` × `Diff`

offspring2=parent2 + `RecMx` × `range` × `MutOpt(2)` × `delta` × (−`Diff`)

$\texttt{RecMx} = \pm 1$ with probability `MutOpt(1)`, (- with probability 0.9)
else 0

$\texttt{range} = 0.5 \times$ domain of variable (search interval defined by `FieldDR`).

$$\texttt{delta} = \sum_{i=0}^{m-1} \alpha_i 2^{-i},\ \alpha_i = 1 \text{ with probability 1/m, else 0, m = 20.}$$

$$\texttt{Diff} = \frac{\texttt{parent2} - \texttt{parent1}}{\|\texttt{parent1} - \texttt{parent2}\|}$$

The recombination operator `recmut` generates offspring in a direction defined by the parents (line recombination). It tests more often outside the area defined by the parents and in the direction of parent1. The point for the offspring is defined by features of the mutation operator. The probability of small step sizes is greater than that of bigger steps (see `mutbga`).

See Also

`mutate`, `mutbga`, `reclin`

Reference

[1] H. Mühlenbein, “The Breeder Genetic Algorithm - a provable optimal search algorithm and its application”, *IEE Colloquium, Applications of Genetic Algorithms*, Digest No. 94/067, London, 15th March 1994.

[2] H. Mühlenbein and D. Schlierkamp-Voosen, “Predictive Models for the Breeder Genetic Algorithm: I. Continuous Parameter Optimization”, *Evolutionary Computation*, Vol. 1, No. 1, pp.25-49, 1993.

recombin

Purpose

Recombination of individuals (high-level function).

Synopsis

```
NewChrom = recombin(REC_F, Chrom)
NewChrom = recombin(REC_F, Chrom, RecOpt)
NewChrom = recombin(REC_F, Chrom, RecOpt, SUBPOP)
```

Description

`recombin` performs recombination of individuals from a population, `Chrom`, and returns the recombined individuals in a new population, `NewChrom`. Each row of `Chrom` and `NewChrom` corresponds to one individual.

`REC_F` is a string that contains the name of the low-level recombination function, e.g. `recdis` or `xovsp`.

`RecOpt` is an optional parameter specifying the crossover rate. If `RecOpt` is omitted or `NaN`, a default value is assumed.

`SUBPOP` is an optional parameter and determines the number of subpopulations in `Chrom`. If `SUBPOP` is omitted or `NaN`, `SUBPOP = 1` is assumed. All subpopulations in `Chrom` must have the same size.

Example

For examples see `recdis`, `recint`, `reclin`, `xovsp`, `xovdp` and `xovmp`.

Algorithm

`recombin` checks the consistency of the input parameters and calls the low-level recombination function. If `recombin` is called with more than one subpopulation then the low-level recombination function is called separately for each subpopulation.

See Also

`recdis`, `recint`, `reclin`, `xovsp`, `xovdp`, `xovsh`, `mutate`, `select`

reins

Purpose

Reinsertion of offspring in the population.

Synopsis

```
Chrom = reins(Chrom, SelCh)

Chrom = reins(Chrom, SelCh, SUBPOP)

Chrom = reins(Chrom, SelCh, SUBPOP, InsOpt, ObjVCh)

[Chrom, ObjVCh]= reins(Chrom, SelCh, SUBPOP, InsOpt,
   ObjVCh, ObjVSel)
```

Description

`reins` performs insertion of offspring into the current population, replacing parents with offspring and returning the resulting population. The offspring are contained in the matrix `SelCh` and the parents in the matrix `Chrom`. Each row in `Chrom` and `Selch` corresponds to one individual.

`SUBPOP` is an optional parameter and indicates the number of subpopulations in `Chrom` and `SelCh`. If `SUBPOP` is omitted or `NaN`, `SUBPOP = 1` is assumed. All subpopulations in `Chrom` and `SelCh` each must have the same size.

`InsOpt` is an optional vector with a maximum of 2 parameters:

`InsOpt(1)`:

scalar indicating the selection method for replacing parents with offspring:
0 - uniform selection, offspring replace parents uniformly at random
1 - fitness-based selection, offspring replace least fit parents
If omitted or `NaN`, `InsOpt(1) = 0` is assumed

`InsOpt(2)`:

scalar containing the rate of reinsertion of offspring per subpopulation as a fraction of subpopulation size in the range `[0, 1]`.
If omitted or `NaN`, `InsOpt(2) = 1.0` is assumed.
If `INSR = 0` no insertion takes place.
If `INSR` is not 1.0 `ObjVSel` is needed for selecting the best offspring for insertion (truncation selection between offspring).

If `InsOpt` is omitted or `NaN`, then the default values are assumed.

`ObjVCh` is an optional column vector containing the objective values of the individuals in `Chrom`. `ObjVCh` is needed for fitness-based reinsertion.

`ObjVSel` is an optional column vector containing the objective values of the individuals in `SelCh`. `ObjVSel` is required if the number of offspring is greater

than the number of offspring to be reinserted into the population. In this case, offspring are selected for reinsertion according to their fitness.

If `ObjVCh` is output parameter, `ObjVCh` and `ObjVSel` are needed as input parameters. The objective values are then copied, according to the insertion of the offspring, saving the recomputation of the objective values for the whole population.

Example

Consider a population of 8 parents, `Chrom`, and a population of 6 offspring, `SelCh`:

```
Chrom = [1; 2; 3; 4; 5; 6; 7; 8]
SelCh = [11; 12; 13; 14; 15; 16]
```

Insert all offspring in the population:

```
Chrom = reins(Chrom, SelCh)
```

Thus, a new population `Chrom` is produced, e.g.:

```
Chrom =
   12
   11
   15
   16
    5
   13
   14
    8
```

Consider the following `ObjVCh` vector for the parent population `Chrom` and `ObjVSel` for the offspring, `SelCh`:

```
ObjVCh = [21; 22; 23; 24; 25; 26; 27; 28];
ObjVSel= [31; 32; 33; 34; 35; 36]
```

Insert all offspring fitness-based, i.e. replace least fit parents:

```
Chrom = reins(Chrom, SelCh, 1, 1, ObjVCh)
```

```
Chrom =
    1
    2
   16
   15
   14
   13
   12
   11
```

Insert 50% of the offspring fitness-based and copy the objective values according the insertion of offspring:

```
[Chrom, ObjVCh] = reins(Chrom, SelCh, 1, [1 0.5],...
  ObjVCh, ObjVSel)
```

```
Chrom =
    1
    2
    3
    4
    5
   13
   12
   11
```

```
ObjVCh =
   21
   22
   23
   24
   25
   33
   32
   31
```

Consider Chrom and SelCh consist of 2 subpopulations. Insert all offspring in the appropriate subpopulations:

```
Chrom = reins(Chrom, SelCh, 2)
```

```
Chrom =
   12
    2
   13
   11
   14
    6
   15
   16
```

See Also

`select`

rep

Purpose

Matrix replication.

Synopsis

```
MatOut = rep(MatIn, REPN)
```

Description

`rep` is a low-level replication function. Not normally used directly, `rep` is called by a number of functions in the GA-Toolbox.

`rep` performs replication of a matrix, `MatIn`, specified by the numbers in `REPN` and returns the replicated matrix, `MatOut`.

`REPN` contains the number of replications in every direction. `REPN(1)` specifies the number of vertical replications, `REPN(2)` the number of horizontal replications.

Example

Consider the following matrix `MatIn`:

```
MatIn = [
   1 2 3 4;
   5 6 7 8]
```

To perform matrix replication:

```
MatOut = rep(MatIn, [1 2])

MatOut =
   1 2 3 4 1 2 3 4
   5 6 7 8 5 6 7 8

MatOut = rep(MatIn, [2 1])

MatOut =
   1 2 3 4
   5 6 7 8
   1 2 3 4
   5 6 7 8

MatOut = rep(MatIn, [2 3])

MatOut =
   1 2 3 4 1 2 3 4 1 2 3 4
   5 6 7 8 5 6 7 8 5 6 7 8
   1 2 3 4 1 2 3 4 1 2 3 4
   5 6 7 8 5 6 7 8 5 6 7 8
```

Purpose

Roulette wheel selection

Synopsis

```
NewChrIx = rws(FitnV, Nsel)
```

Description

`rws` probabilistically selects `Nsel` individuals for reproduction according to their fitness, `FitnV`, in the current population.

`NewChrIx = rws(FitnV, Nsel)` selects `Nsel` individuals from a population using roulette wheel selection. `FitnV` is a column vector containing a performance measure for each individual in the population. This can be achieved by using the function `ranking` or `scaling` to assign a fitness level to each individual. The return value, `NewChrIx`, is the index of the individuals selected for breeding, in the order that they were selected. The selected individuals can be recovered by evaluating `Chrom(NewChrIx,:)`.

`rws` is a low-level selection function normally called by `select`.

Example

Consider a population of 8 individuals with the assigned fitness values, `FitnV`:

```
FitnV = [1.50; 1.35; 1.21; 1.07; 0.92; 0.78; 0.64; 0.5]
```

Select the indices of 6 individuals:

```
NewChrIx = rws(FitnV, 6)
```

Thus, `NewChrIx` can become:

```
NewChrIx =
   2
   5
   1
   1
   3
   7
```

Algorithm

A form of roulette wheel selection is implemented by obtaining a cumulative sum of the fitness vector, `FitnV`, and generating `Nsel` uniformly at random distributed numbers between `0` and `sum(FitnV)`. The index of the individuals selected is determined by comparing the generated numbers with the cumulative sum vector. The probability of an individual being selected is then given by:

$$F(x_i) = \frac{f(x_i)}{\sum_{i=1}^{N_{ind}} f(x_i)},$$

where $f(x_i)$ is the fitness of individual x_i and $F(x_i)$ is the probability of that individual being selected.

See Also

`select`, `sus`, `reins`, `ranking`, `scaling`

Reference

[1] J. E. Baker, “Reducing bias and inefficiency in the selection algorithm”, *Proc ICGA 2*, pp. 14-21, Lawrence Erlbaum Associates, Publishers, 1987.

[2] David E. Goldberg, *Genetic Algorithms in Search, Optimization and Machine Learning*, Addison Wesley, 1989.

scaling

Purpose

Linear fitness scaling

Synopsis

```
FitnV = scaling(ObjV, Smul)
```

Description

`scaling` converts the objective values, `ObjV`, of a population into a fitness measure with a known upper bound, determined by the value of `Smul`, such that,

$$F(x_i) = af(x_i) + b,$$

where $f(x_i)$ is the objective value of individual x_i, a is a scaling coefficient, b is an offset and $F(x_i)$ is the resulting fitness value of individual x_i. If f_{ave} is the average objective value in the current generation, then the maximum fitness of the scaled population is upper bounded at $f_{ave} \times$ `Smul`. If `Smul` is omitted then the default value of `Smult = 2` is assumed. The average fitness of the scaled population is also set to f_{ave}.

In the case of some of the objective values being negative, scaling attempts to provide an offset, b, such that the scaled fitness values are greater than zero.

Algorithm

`scaling` uses the linear scaling method described by Goldberg [1].

Note: linear scaling is not suitable for use with objective functions that return negative fitness values and is included here only for completeness.

See Also

`ranking`, `reins`, `rws`, `select`, `sus`

Reference

[1] D. E. Goldberg, *Genetic Algorithms in Search, Optimization and Machine Learning*, Addison Wesley Publishing Company, January 1989.

select

Purpose

Selection of individuals from population (high-level function).

Synopsis

```
SelCh = select(SEL_F, Chrom, FitnV)
SelCh = select(SEL_F, Chrom, FitnV, GGAP)
SelCh = select(SEL_F, Chrom, FitnV, GGAP, SUBPOP)
```

Description

`select` performs selection of individuals from a population, `Chrom`, and returns the selected individuals in a new population, `SelCh`. Each row of `Chrom` and `SelCh` corresponds to one individual.

`SEL_F` is a string and contains the name of the low-level selection function, for example `rws` or `sus`.

`FitnV` is a column vector containing the fitness values of the individuals in `Chrom`. The fitness value indicates the expected probability of selection of each individual.

`GGAP` is an optional parameter specifying the generation gap, the fraction of the population to be reproduced. If `GGAP` is omitted or `NaN, GGAP = 1.0` (100%) is assumed. `GGAP` may also be greater than 1, allowing more offspring to be produced then the number of parents. If `Chrom` consists of more than one subpopulation, `GGAP` specifies the number of individuals to be selected per subpopulation relative to the size of the subpopulation.

`SUBPOP` is an optional parameter and determines the number of subpopulations in `Chrom`. If `SUBPOP` is omitted or `NaN, SUBPOP = 1` is assumed. All subpopulations in `Chrom` must have the same size.

Example

Consider a population of 8 individuals, `Chrom`, with the assigned fitness values, `FitnV`:

```
Chrom = [
   1 11 21;
   2 12 22;
   3 13 23;
   4 14 24;
   5 15 25;
   6 16 26;
   7 17 27;
   8 18 28]
```

```
FitnV = [1.50; 1.35; 1.21; 1.07; 0.92; 0.78; 0.64; 0.5]
```

Select 8 individuals by stochastic universal sampling, `sus`:

```
SelCh = select('sus', Chrom, FitnV)
```

Thus, `SelCh` can become:

```
SelCh =
   7 17 27
   1 11 21
   6 16 26
   1 11 21
   5 15 25
   2 12 22
   3 13 23
   4 14 24
```

Consider `Chrom` consists of 2 subpopulations. Select 150% individuals per subpopulation by roulette wheel selection, `rws`:

```
FitnV = [1.50; 1.16; 0.83; 0.50; 1.50; 1.16; 0.83; 0.5]
```

```
SelCh = select('sus', Chrom, FitnV, 1.5, 2)
```

Thus, `SelCh` can become:

```
SelCh =
   3 13 23
   2 12 22
   1 11 21
   2 12 22
   2 12 22
   1 11 21
   6 16 26
   7 17 27
   7 17 27
   6 16 26
   7 17 27
   5 15 25
```

Algorithm

`select` checks the consistency of the input parameter and calls the low-level selection function. If `select` is called with more than one subpopulation then the low-level selection function is called separately for each subpopulation.

See Also

`rws`, `sus`, `ranking`, `scaling`, `recombin`, `mutate`

Purpose

Stochastic universal sampling

Synopsis

```
NewChrIx = sus(FitnV, Nsel)
```

Description

`sus` probabilistically selects `Nsel` individuals for reproduction according to their fitness, `FitnV`, in the current population.

`NewChrIx = rws(FitnV, Nsel)` selects `Nsel` individuals from a population using stochastic universal sampling [1]. `FitnV` is a column vector containing a performance measure for each individual in the population. This can be achieved by using the function `ranking` or `scaling` to assign a fitness level to each individual. The return value, `NewChrIx`, is the index of the individuals selected for breeding, in the order that they were selected. The selected individuals can be recovered by evaluating `Chrom(NewChrIx,:)`.

`sus` is a low-level selection function normally called by `select`.

Example

Consider a population of 8 individuals with the assigned fitness values, `FitnV`:

```
FitnV = [1.50; 1.35; 1.21; 1.07; 0.92; 0.78; 0.64; 0.5]
```

Select the indices of 6 individuals:

```
NewChrIx = sus(FitnV, 6)
```

Thus, `NewChrIx` can become:

```
NewChrIx =
   5
   6
   3
   1
   1
   2
```

Algorithm

A form of stochastic universal sampling is implemented by obtaining a cumulative sum of the fitness vector, `FitnV`, and generating `Nsel` equally spaced numbers between `0` and `sum(FitnV)`. Thus, only one random number is generated, all the others used being equally spaced from that point. The index of the individuals

selected is determined by comparing the generated numbers with the cumulative sum vector. The probability of an individual being selected is then given by

$$F(x_i) = \frac{f(x_i)}{\sum_{i=1}^{N_{ind}} f(x_i)},$$

where $f(x_i)$ is the fitness of individual x_i and $F(x_i)$ is the probability of that individual being selected.

See Also

`select`, `rws`, `reins`, `ranking`, `scaling`

Reference

[1] J. E. Baker, "Reducing bias and inefficiency in the selection algorithm", *Proc. ICGA 2*, pp. 14-21, Lawrence Erlbaum Associates, Publishers, 1987.

xovdp

Purpose

Double-point crossover

Synopsis

```
NewChrom = xovdp(OldChrom, XOVR)
```

Description

`xovdp` performs double-point crossover between pairs of individuals contained in the current population, `OldChrom`, according to the crossover probability, `XOVR`, and returns a new population after mating, `NewChrom`. Each row of `OldChrom` and `NewChrom` corresponds to one individual. For the chromosomes any representation can be used.

`XOVR` is an optional parameter specifying the crossover rate. If `XOVR` is omitted, empty or `NaN, XOVR = 0.7` is assumed.

The pairs are mated in order, odd row with the next even row. If the number of rows in the matrix `OldChrom` is odd then the last row is not mated. The population should therefore be organised into contiguous pairs that require mating. This can be achieved by using the function `ranking` to assign a fitness level to each chromosome and a selection function (`select`, `sus` or `rws`) to select individuals with a probability related to their fitness in the current population.

`xovdp` is a low-level crossover function normally called by `recombin`.

Algorithm

Consider the following two binary strings of the same length:

```
A1 = [1 1 0 1 0 1]
A2 = [1 0 1 0 1 0]
```

Double point crossover involves selecting uniformly at random two integer positions, *k1* and *k2*, between 1 and `length(A1)`, and swapping the variables in positions *k1+1* to *k2* between `A1` and `A2`. Thus if the crossover positions *k1* = 3 and *k2* = 5, then `A1` and `A2` would become:

```
A1' = [1 1 0 0 1 1]
A2' = [1 0 1 1 0 0]
```

`xovdp` calls `xovmp` with the appropriate parameters.

See Also

`xovdprs`, `xovsp`, `xovsh`, `xovmp`, `recombin`, `select`

xovdprs

Purpose

Double-point reduced surrogate crossover

Synopsis

`NewChrom = xovdprs(OldChrom, XOVR)`

Description

`xovdprs` performs double-point reduced surrogate crossover between pairs of individuals contained in the current population, `OldChrom`, according to the crossover probability, `XOVR`, and returns a new population after mating, `NewChrom`. Each row of `OldChrom` and `NewChrom` corresponds to one individual. For the chromosomes any representation can be used.

`XOVR` is an optional parameter specifying the crossover rate. If `XOVR` is omitted, empty or `NaN, XOVR = 0.7` is assumed.

The pairs are mated in order, odd row with the next even row. If the number of rows in the matrix `OldChrom` is odd then the last row is not mated. The population should therefore be organised into contiguous pairs that require mating. This can be achieved by using the function `ranking` to assign a fitness level to each chromosome and a selection function (`select`, `sus` or `rws`) to select individuals with a probability related to their fitness in the current population.

`xovdprs` is a low-level crossover function normally called by `recombin`.

Algorithm

For double point crossover see `xovdp`.

The *reduced surrogate* operator constrains crossover to always produce new individuals wherever possible. This is implemented by restricting the location of crossover points such that crossover points only occur where gene values differ [1].

`xovdprs` calls `xovmp` with the appropriate parameters.

See Also

`xovdp`, `xovsprs`, `xovshrs`, `xovmp`, `recombin`, `select`

Reference

[1] L. Booker, “Improving search in genetic algorithms,” In *Genetic Algorithms and Simulated Annealing*, L. Davis (Ed.), pp. 61-73, Morgan Kaufmann Publishers, 1987 .

xovmp

Purpose

Multi-point crossover

Synopsis

`NewChrom = xovmp(OldChrom, XOVR, Npt, Rs)`

Description

`xovmp` performs multi-point crossover between pairs of individuals contained in the current population, `OldChrom`, and returns a new population after mating, `NewChrom`. Each row of `OldChrom` and `NewChrom` corresponds to one individual. For the chromosomes any representation can be used.

`XOVR` is an optional parameter specifying the crossover rate. If `XOVR` is omitted, empty or `NaN`, `XOVR = 0.7` is assumed.

`Npt` is an optional parameter specifying the number of crosspoints:

0 - shuffle crossover.
1 - single point crossover.
2 - double point crossover.
If `Npt` is omitted, empty or `NaN`, `Npt = 0` is assumed.

`Rs` is an optional parameter specifying the use of reduced surrogate:

0 - no reduced surrogate.
1 - use reduced surrogate.
If `Rs` is omitted, empty or `NaN`, `Rs = 0` is assumed.

The pairs are mated in order, odd row with the next even row. If the number of rows in the matrix `OldChrom` is odd then the last row is not mated. The population should therefore be organised into contiguous pairs that require mating. This can be achieved by using the function `ranking` to assign a fitness level to each chromosome and a selection function (`select`, `sus` or `rws`) to select individuals with a probability related to their fitness in the current population.

`xovmp` is a low-level crossover function called by all other crossover functions. If called by `recombine xovmp` performs shuffle crossover without reduced surrogate identical to `xovsh`.

Algorithm

The algorithms used in single-point, double-point and shuffle crossover are described in the `xovsp`, `xovdp and xovsh` *Reference* entries respectively. The algorithms used in single-point, double-point and shuffle crossover with reduced surrogates are described in the `xovsprs`, `xovdprs and xovshrs` *Reference* entries respectively.

See Also

`xovsp`, `xovdp`, `xovsh`, `xovsprs`, `xovdprs`, `xovshrs`, `recombin`

xovsh

Purpose

Shuffle crossover

Synopsis

```
NewChrom = xovsh(OldChrom, XOVR)
```

Description

`xovsh` performs shuffle crossover between pairs of individuals contained in the current population, `OldChrom`, according to the crossover probability, `XOVR`, and returns a new population after mating, `NewChrom`. Each row of `OldChrom` and `NewChrom` corresponds to one individual. For the chromosomes any representation can be used.

`XOVR` is an optional parameter specifying the crossover rate. If `XOVR` is omitted, empty or `NaN, XOVR = 0.7` is assumed.

The pairs are mated in order, odd row with the next even row. If the number of rows in the matrix `OldChrom` is odd then the last row is not mated. The population should therefore be organised into contiguous pairs that require mating. This can be achieved by using the function `ranking` to assign a fitness level to each chromosome and a selection function (`select`, `sus` or `rws`) to select individuals with a probability related to their fitness in the current population.

`xovsh` is a low-level crossover function normally called by `recombin`.

Algorithm

Shuffle crossover is single-point crossover (see `xovsp`), but before the bits are exchanged, they are randomly shuffled in both parents. After recombination, the bits in the offspring are unshuffled. This removes positional bias as the bits are randomly reassigned each time crossover is performed [1].

`xovsh` calls `xovmp` with the appropriate parameters.

See Also

`xovshrs`, `xovsp`, `xovdp`, `xovmp`, `recombin`, `select`

Reference

[1] R. A. Caruana, L. A. Eshelman, J. D. Schaffer, “Representation and hidden bias II: Eliminating defining length bias in genetic search via shuffle crossover”, In *Eleventh International Joint Conference on Artificial Intelligence*, N. S. Sridharan (Ed.), Vol. 1, pp. 750-755, Morgan Kaufmann Publishers, 1989.

xovshrs

Purpose

Shuffle crossover with reduced surrogate

Synopsis

`NewChrom = xovshrs(OldChrom, XOVR)`

Description

`xovshrs` performs shuffle crossover with reduced surrogates between pairs of individuals contained in the current population, `OldChrom`, according to the crossover probability, `XOVR`, and returns a new population after mating, `NewChrom`. Each row of `OldChrom` and `NewChrom` corresponds to one individual. For the chromosomes any representation can be used.

`XOVR` is an optional parameter specifying the crossover rate. If `XOVR` is omitted, empty or `NaN`, `XOVR = 0.7` is assumed.

The pairs are mated in order, odd row with the next even row. If the number of rows in the matrix `OldChrom` is odd then the last row is not mated. The population should therefore be organised into contiguous pairs that require mating. This can be achieved by using the function `ranking` to assign a fitness level to each chromosome and a selection function (`select`, `sus` or `rws`) to select individuals with a probability related to their fitness in the current population.

`xovshrs` is a low-level crossover function normally called by `recombin`.

Algorithm

For shuffle crossover algorithm see `xovsh`.

The *reduced surrogate* operator constrains crossover to always produce new individuals wherever possible. This is implemented by restricting the location of crossover points such that crossover points only occur where gene values differ [1].

`xovshrs` calls `xovmp` with the appropriate parameters.

See Also

`xovsh`, `xovsprs`, `xovdprs`, `xovmp`, `recombin`, `select`

Reference

[1] L. Booker, “Improving search in genetic algorithms,” In *Genetic Algorithms and Simulated Annealing*, L. Davis (Ed.), pp. 61-73, Morgan Kaufmann Publishers, 1987.

xovsp

Purpose

Single-point crossover

Synopsis

```
NewChrom = xovsp(OldChrom, XOVR)
```

Description

`xovsp` performs single-point crossover between pairs of individuals contained in the current population, `OldChrom`, according to the crossover probability, `XOVR`, and returns a new population after mating, `NewChrom`. `OldChrom` contains the chromosomes of the current population, each row corresponds to one individual. For the chromosomes any representation can be used.

`XOVR` is an optional parameter specifying the crossover rate. If `XOVR` is omitted, empty or `NaN, XOVR = 0.7` is assumed.

The pairs are mated in order, odd row with the next even row. If the number of rows in the matrix `OldChrom` is odd then the last row is not mated. The population should therefore be organised into contiguous pairs that require mating. This can be achieved by using the function `ranking` to assign a fitness level to each chromosome and a selection function (`select`, `sus` or `rws`) to select individuals with a probability related to their fitness in the current population.

`xovsp` is a low-level crossover function normally called by `recombin`.

Algorithm

Consider the following two binary strings of the same length:

```
A1 = [1 1 0 1 0 1]
A2 = [1 0 1 0 1 0]
```

Single-point crossover involves selecting uniformly at random an integer position, *k*, between 1 and `(length(A1)-1)`, and swapping the variables in positions *k+1* to `length(A1)` between `A1` and `A2`. Thus if the crossover position $k = 3$, then `A1` and `A2` would become:

```
A1′ = [1 1 0 0 1 0]
A2′ = [1 0 1 1 0 1]
```

`xovsp` calls `xovmp` with the appropriate parameters.

See Also

`xovsprs`, `xovdp`, `xovsh`, `xovmp`, `recombin`, `select`

xovsprs

Purpose

Single-point reduced surrogate crossover

Synopsis

`NewChrom = xovsprs(OldChrom, XOVR)`

Description

`xovsprs` performs single-point reduced surrogate crossover between pairs of individuals contained in the current population, `OldChrom`, according to the crossover probability, `XOVR`, and returns a new population after mating, `NewChrom`. `OldChrom` contains the chromosomes of the current population, each row corresponds to one individual. For the chromosomes any representation can be used.

`XOVR` is an optional parameter specifying the crossover rate. If `XOVR` is omitted, empty or `NaN, XOVR = 0.7` is assumed.

The pairs are mated in order, odd row with the next even row. If the number of rows in the matrix `OldChrom` is odd then the last row is not mated. The population should therefore be organised into contiguous pairs that require mating. This can be achieved by using the function `ranking` to assign a fitness level to each chromosome and a selection function (`select`, `sus` or `rws`) to select individuals with a probability related to their fitness in the current population.

`xovsprs` is a low-level crossover function normally called by `recombin`.

Algorithm

For single-point crossover see `xovsp`.

The *reduced surrogate* operator constrains crossover to always produce new individuals wherever possible. This is implemented by restricting the location of crossover points such that crossover points only occur where gene values differ [1].

`xovsprs` calls `xovmp` with the appropriate parameters.

See Also

`xovsp`, `xovdp`, `xovdprs`, `xovsh`, `xovshrs`, `xovmp`, `recombin`, `select`

Reference

[1] L. Booker, “Improving search in genetic algorithms,” In *Genetic Algorithms and Simulated Annealing*, L. Davis (Ed.), pp. 61-73, Morgan Kaufmann Publishers, 1987 .
