# Supplementary figures and images for: Design of Closed-Loop Control Schemes Based on the GA-PID and GA-RBF-PID Algorithms for Brain Dynamic Modulation
Source: Entropy (Basel). 2023 Nov 15;25(11):1544. doi: 10.3390/e25111544 (PMC10670460; doi:10.3390/e25111544)

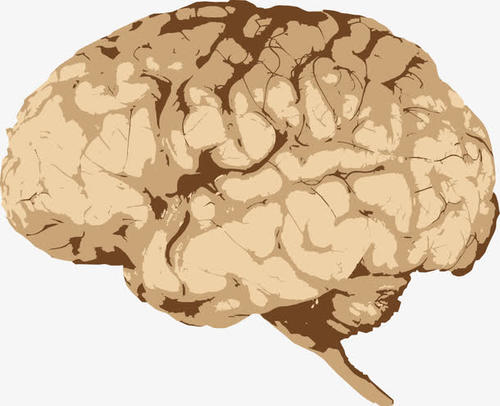

Supplement: Supplementary file 1 [file entropy-25-01544-s001.zip › GA_RBF_PID/mass/brain.jpg]
